# Supplementary material for: A Complete Assessment of Dopamine Receptor- Ligand Interactions through Computational Methods
Source: Molecules. 2019 Mar 27;24(7):1196. doi: 10.3390/molecules24071196 (PMC6479630; doi:10.3390/molecules24071196)
Supplement: Supplementary file 1 [file molecules-24-01196-s001.pdf]

molecules

MDPI

Article

A complete assessment of dopamine receptor-ligand interactions through computational methods

Beatriz Bueschbell[1], Carlos A. V. Barreto[2], António J. Preto[2] Anke C. Schiedel[1] and Irina S. Moreira [2,3]*

1 PharmaCenter Bonn, Pharmaceutical Institute, Pharmaceutical Chemistry I, University of Bonn, D-53121 Bonn, Germany; bueschbell@uni-bonn.de; schiedel@uni-bonn.de

2 Center for Neuroscience and Cell Biology, UC- Biotech Parque Tecnológico de Cantanhede, Núcleo 04, Lote B, 3060-197 Cantanhede, Portugal; cbarreto@cnc.uc.pt; martinsgomes.jose@gmail.com; irina.moreira@cnc.uc.pt.

3 Institute for Interdisciplinary Research, University of Coimbra.

*Correspondence: irina.moreira@cnc.uc.pt; Tel.: +351 231 249 730

SUPPLEMENTARY INFORMATION

FIGURES

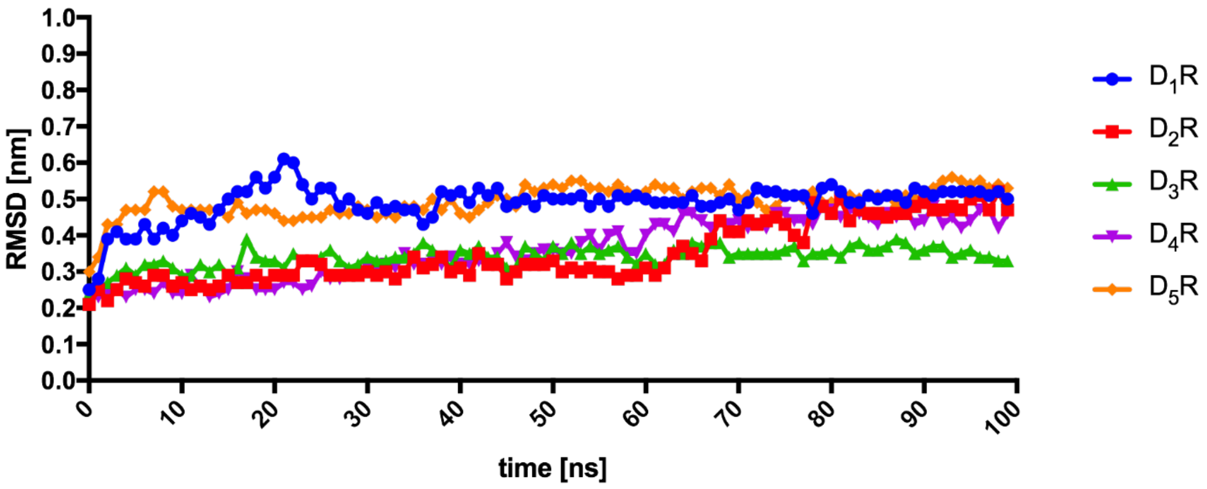

Figure S1 – RMSD throughout the 100 ns of simulation for all DR models.

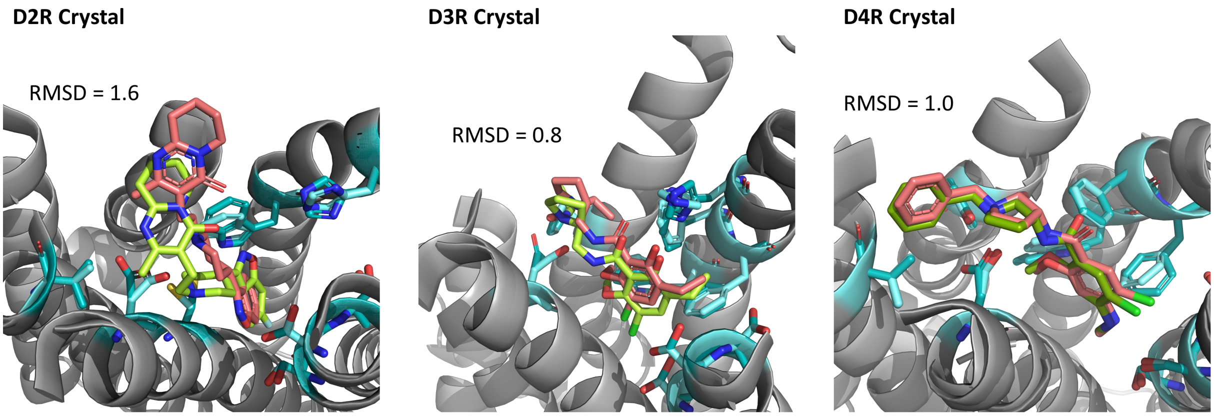

Figure S2 – Redocking of ligands with their respective DR and bound ligand (D2R, D3R and D4R PDB-ids are respectively: 6CM4 [1], 3PBL [2], 5WIU [3]**).** RMSD values between ligand pose in crystal (pink) and docking output (green) are displayed. Interacintg residues were chosen according to Figure 2.

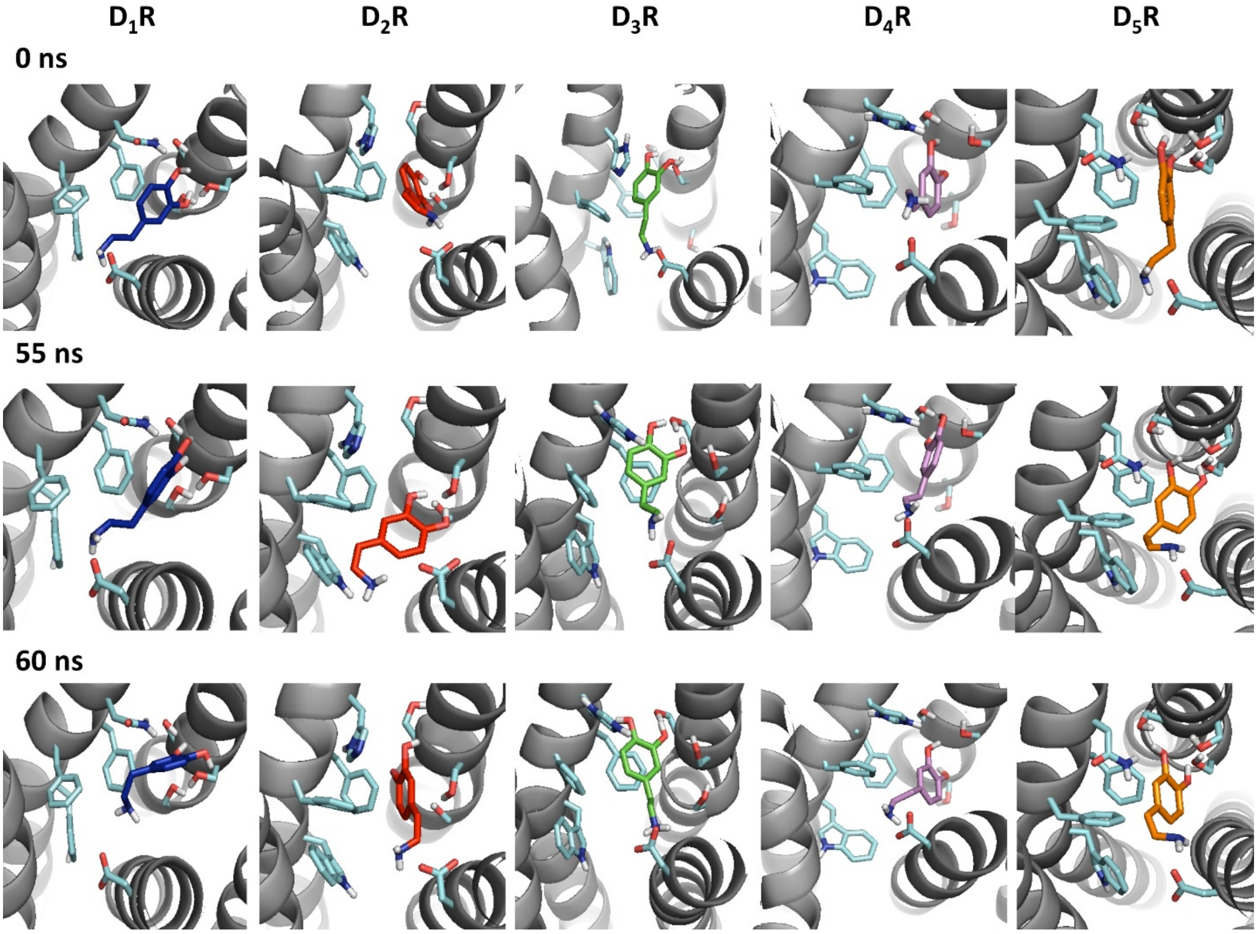

Figure S3 - Molecular docking of Dopamine at the $D_{1\text{-}5}R$ during 0 – 60 ns. The images correspond to the cluster with the lowest binding energy [kcal/mol] and highest number of conformations. D_1R, D_2R, D_3R, D_4R and D_5R were colored blue, red, green, violet and orange, respectively. Interacting residues are colored cyan.

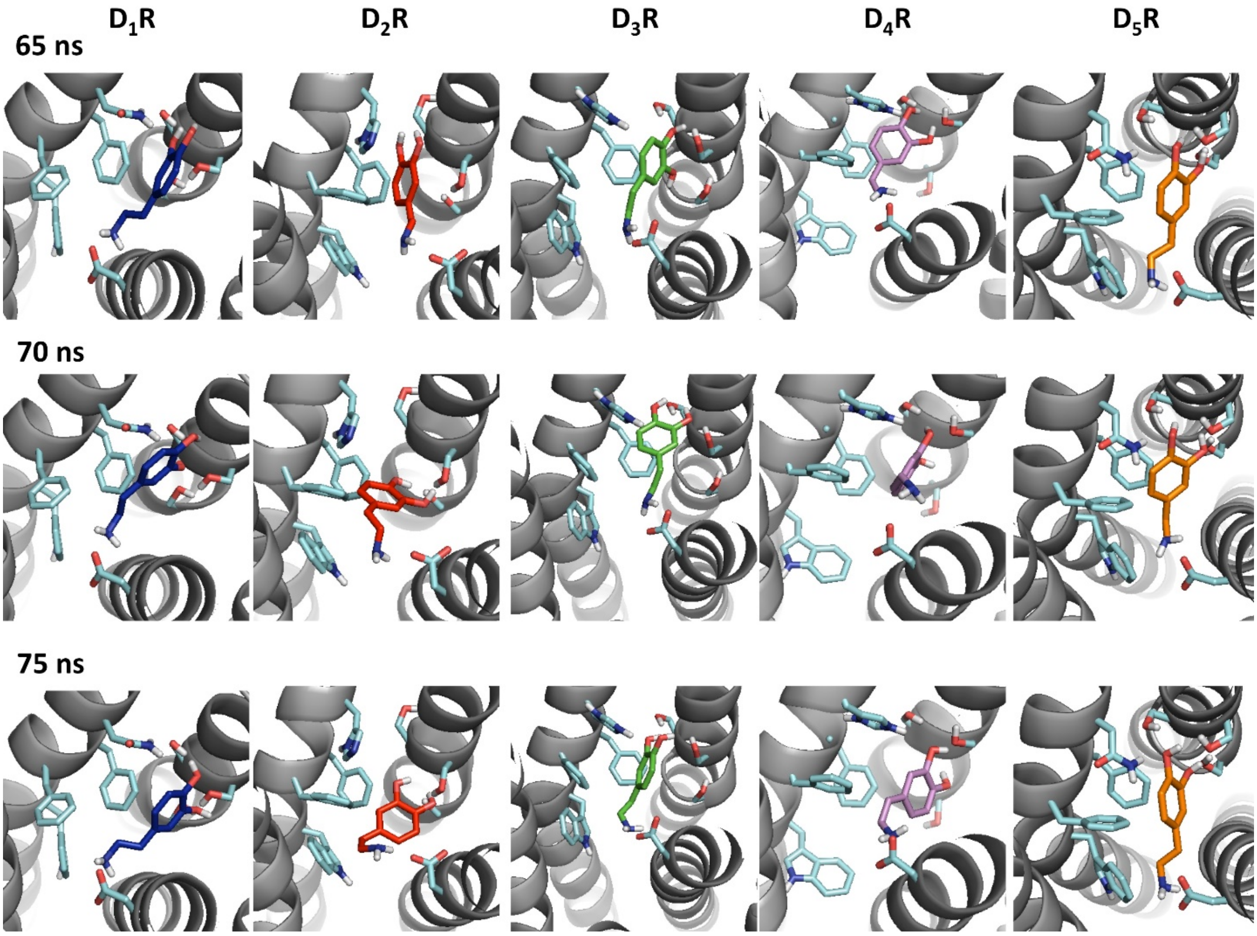

Figure S4 - Molecular docking of Dopamine at the $D_{1-5}R$ during 65 – 75 ns. The images correspond to the cluster with the lowest binding energy [kcal/mol] and highest number of conformations. D_1R, D_2R, D_3R, D_4R and D_5R were colored blue, red, green, violet and orange, respectively. Interacting residues are colored cyan.

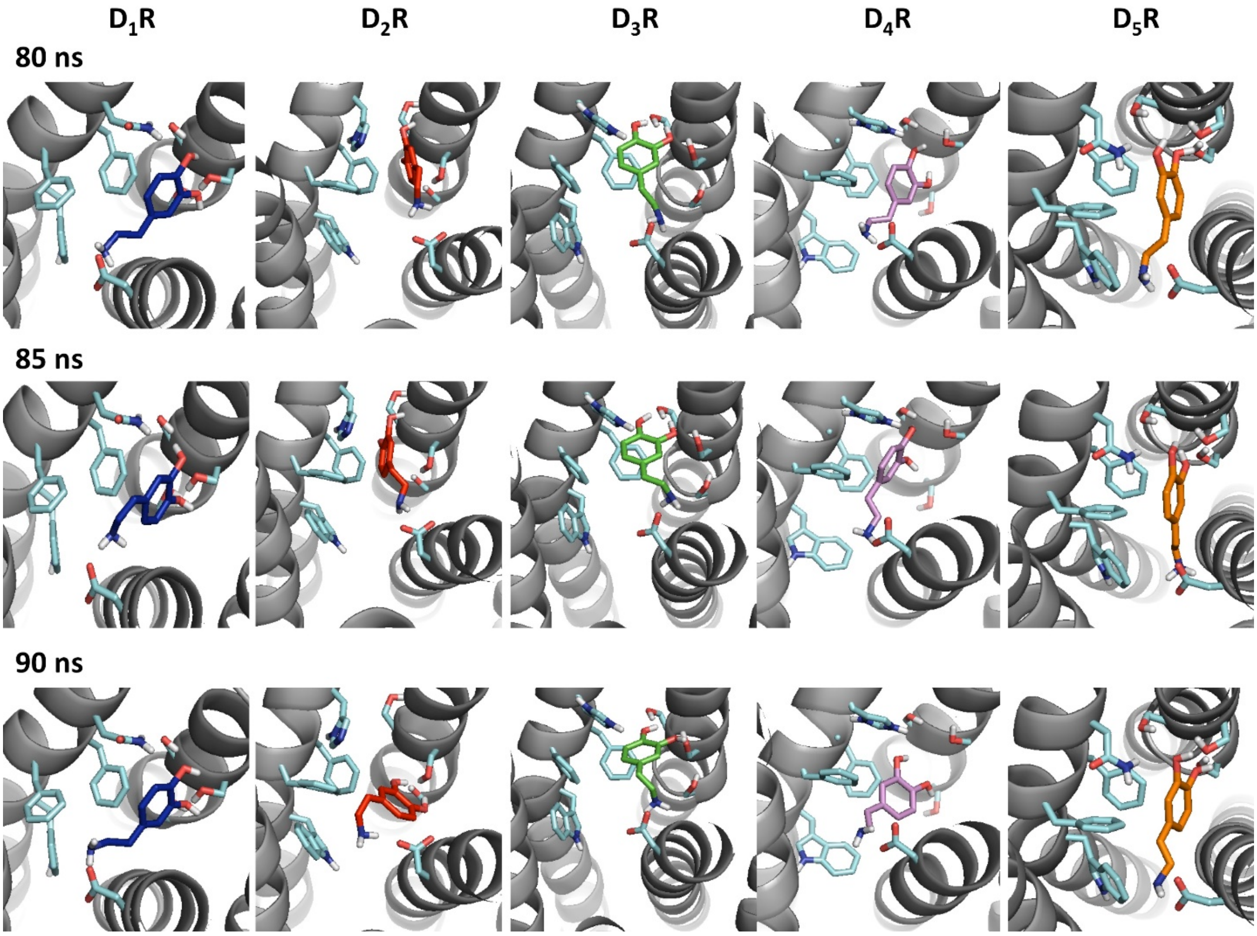

Figure S5 - Molecular docking of Dopamine at the D1-5R during 80 –90 ns. The images correspond to the cluster with the lowest binding energy [kcal/mol] and highest number of conformations. D1R, D2R, D3R, D4R and D5R were colored blue, red, green, violet and orange, respectively. Interacting residues are colored cyan.

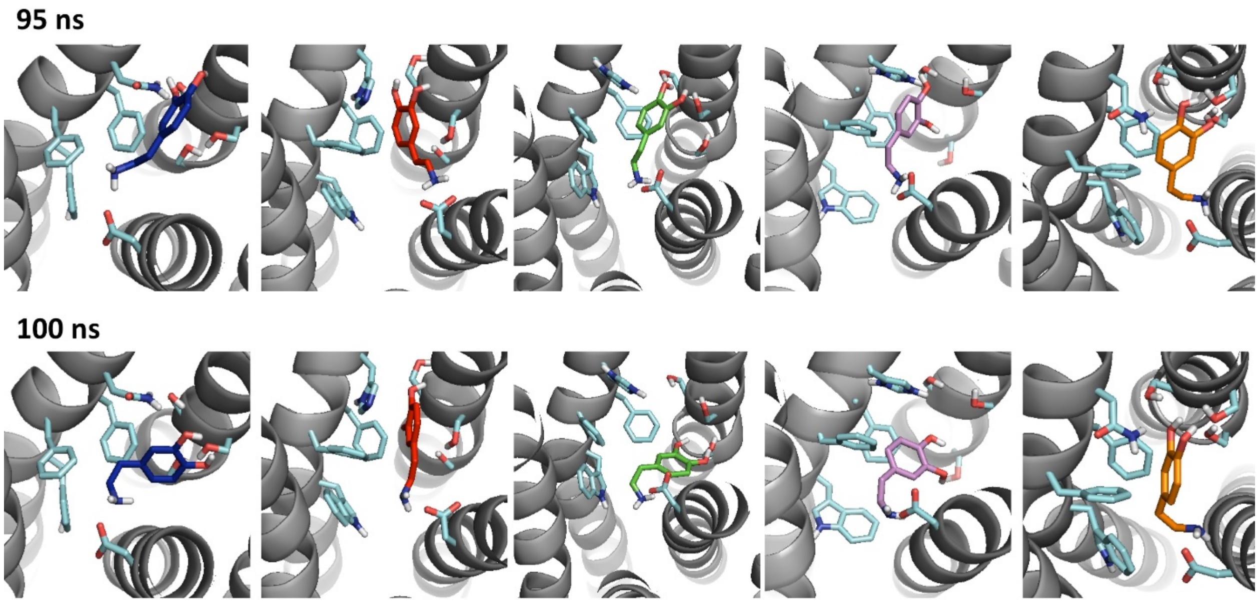

Figure S6 - Molecular docking of Dopamine at the $D_{1\text{-}5}R$ during 95 and 100 ns. The images correspond to the cluster with the lowest binding energy [kcal/mol] and highest number of conformations. D_1R, D_2R, D_3R, D_4R and D_5R were colored blue, red, green, violet and orange, respectively. Interacting residues are colored cyan.

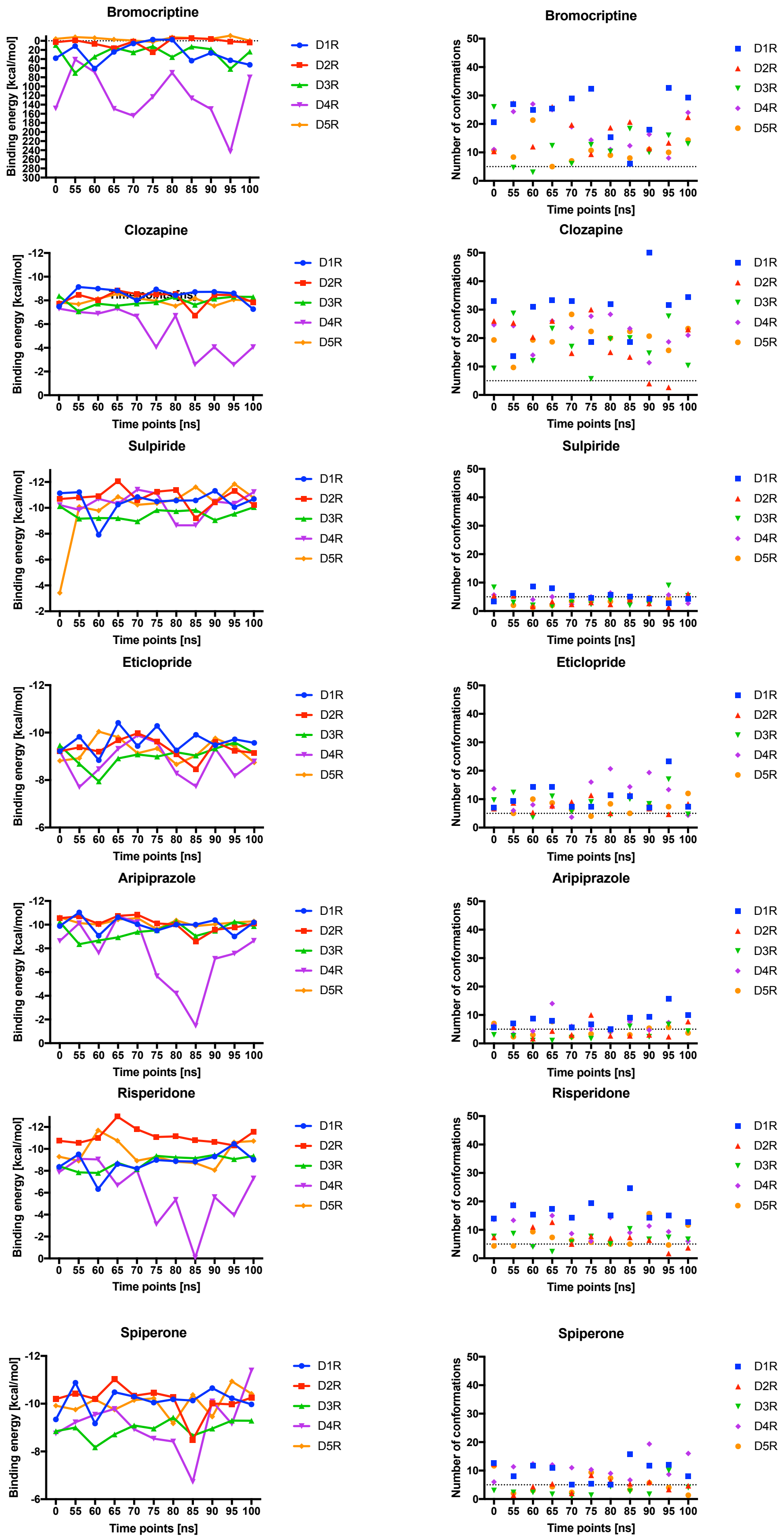
Bromocriptine
Binding energy [kcal/mol]
0 20 40 60 80 100 120 140 160 180 200 220 240 260 280 300
0 55 60 65 70 75 80 85 90 95 100
D1R
D2R
D3R
D4R
D5R
Bromocriptine
Number of conformations
Time points [ns]
Clozapine
Binding energy [kcal/mol]
Time points [ns]
Clozapine
Number of conformations
Time points [ns]
Sulpiride
Binding energy [kcal/mol]
Time points [ns]
Sulpiride
Number of conformations
Time points [ns]
Eticlopride
Binding energy [kcal/mol]
Time points [ns]
Eticlopride
Number of conformations
Time points [ns]
Aripiprazole
Binding energy [kcal/mol]
Time points [ns]
Aripiprazole
Number of conformations
Time points [ns]
Risperidone
Binding energy [kcal/mol]
Time points [ns]
Risperidone
Number of conformations
Time points [ns]
Spiperone
Binding energy [kcal/mol]
Time points [ns]
Spiperone
Number of conformations
Time points [ns]

Figure S7 - Results of the molecular docking of bromocriptine, clozapine, sulpiride, eticlopride, aripiprazole, risperidone and spiperone for all DR subtypes at different receptor conformations in various time points [ns]. The average of the 3 lowest binding energies of dopamine were calculated in the left plots. The number of conformations of the three clusters with the lowest binding energies were plotted for each time point and receptor (right plot).

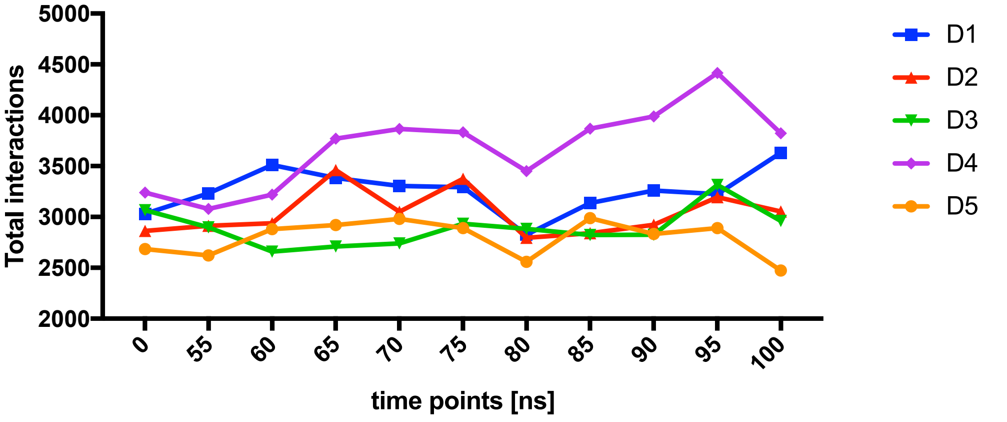

Figure S8 - Total interactions counted for each DR over time points [ns]. The interactions were summarized for all ligands and interaction types. The DR-subtypes are color-coded.

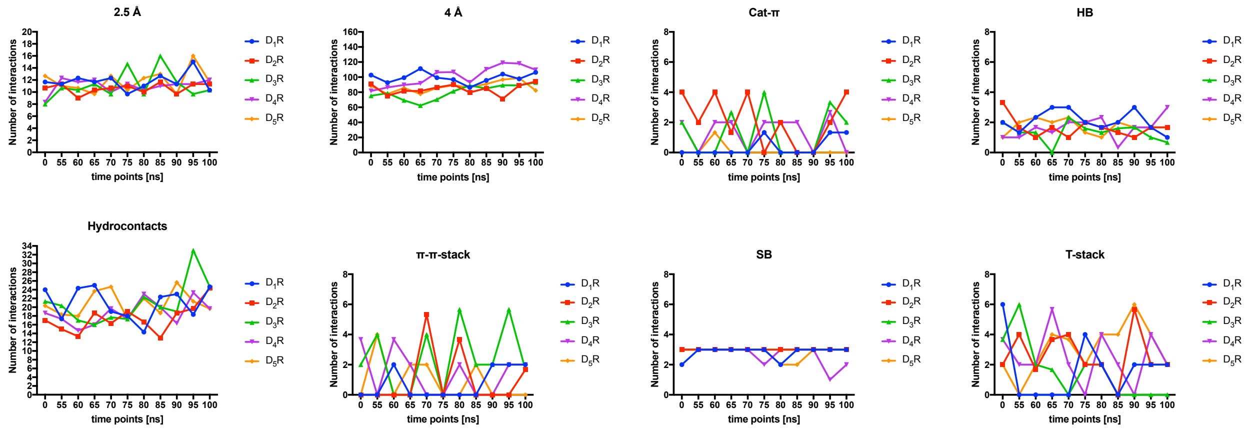

Figure S9 – Pairwise interactions results for dopamine. Each type of interaction is displayed over all time points for each DR. The DR-subtypes are color-coded. Abbreviations: 2.5 Å – interactions within 2.5 Å; 4 Å – interactions within 4 Å; cat-π – cation-π-interactions; HB – hydrogen bonds; π-π-stack – interactions involving π-π-stacking; SB – salt-bridge; T-stack – aromatic superposition (edge-face-interactions).

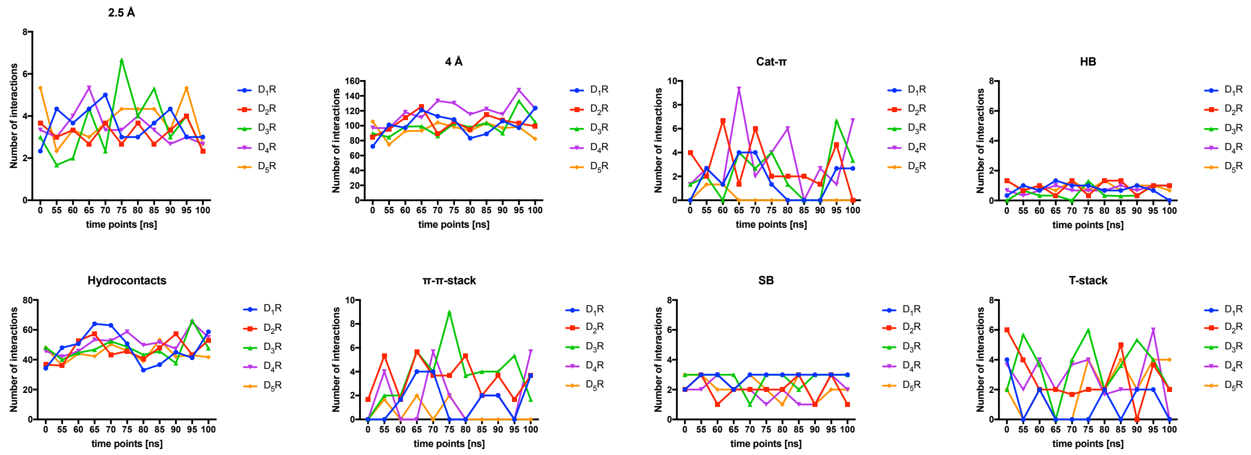

Figure S10 - Pairwise interactions results for 7-OH-DPAT. Each type of interaction is displayed over all time points for each DR. The DR-subtypes are color-coded. Abbreviations: 2.5 Å – interactions within 2.5 Å; 4 Å – interactions within 4 Å; cat-π – cationic π-interactions; HB – hydrogen bonds; π-π-stack – interactions involving π-π-stacking; SB – salt-bridge; T-stack – aromatic superposition (edge-face-interactions).

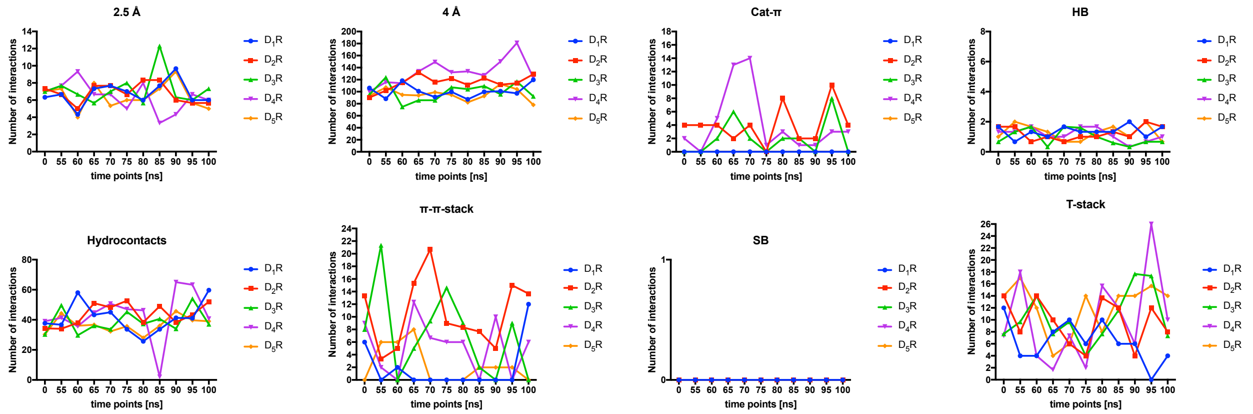

Figure S11 - Pairwise interactions results for apomorphine. Each type of interaction is displayed over all time points for each DR. The DR-subtypes are color-coded. Abbreviations: 2.5 Å – interactions within 2.5 Å; 4 Å – interactions within 4 Å; cat-π – cationic π-interactions; HB – hydrogen bonds; π-π-stack – interactions involving π-π-stacking; SB – salt-bridge; T-stack – aromatic superposition (edge-face-interactions).

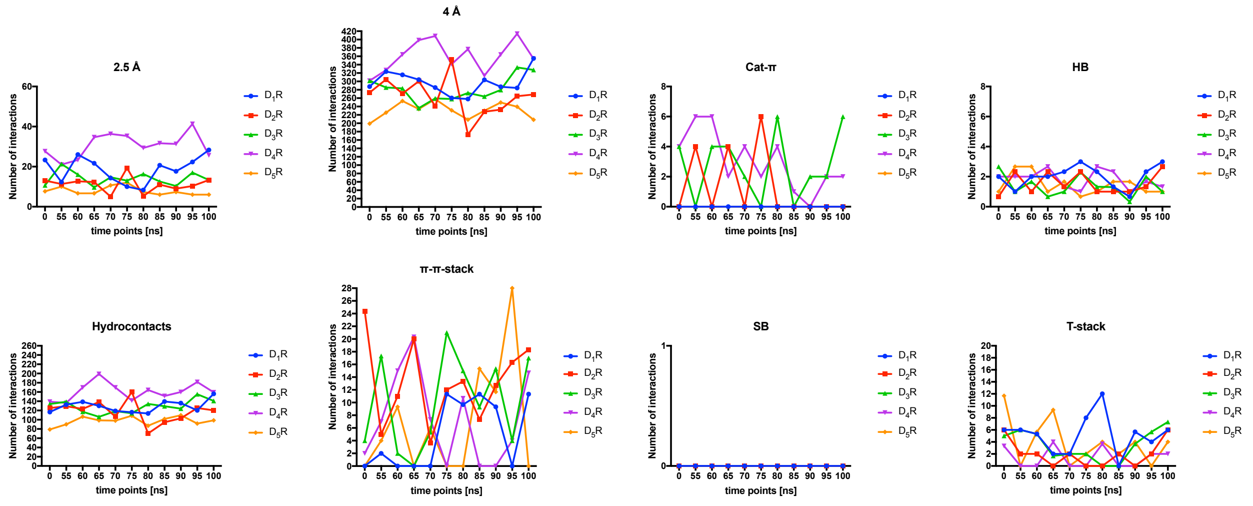

Figure S12 - Pairwise interactions results for bromocriptine. Each type of interaction is displayed over all time points for each DR. The DR-subtypes are color-coded. Abbreviations: 2.5 Å – interactions within 2.5 Å; 4 Å – interactions within 4 Å; cat-π – cationic π-interactions; HB – hydrogen bonds; π-π-stack – interactions involving π-π-stacking; SB – salt-bridge; T-stack – aromatic superposition (edge-face-interactions).

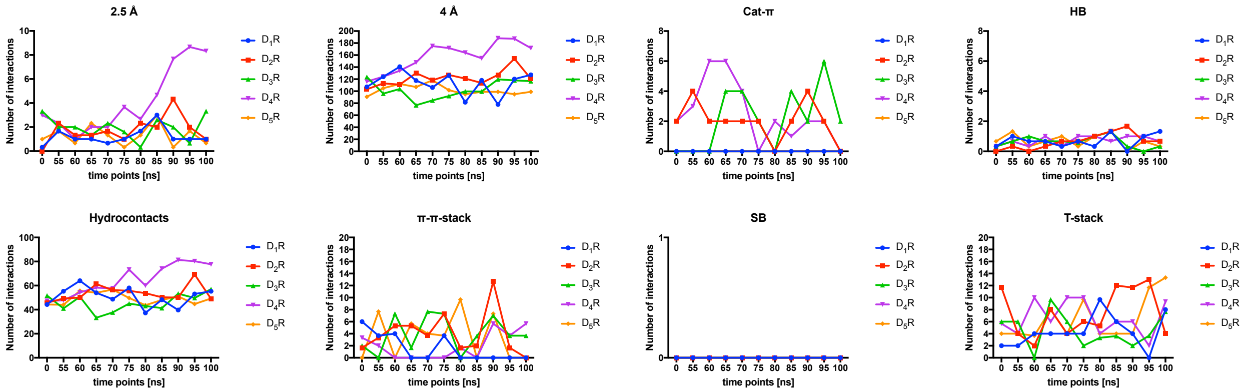

Figure S13 - Pairwise interactions results for clozapine. Each type of interaction is displayed over all time points for each DR. The DR-subtypes are color-coded. Abbreviations: 2.5 Å – interactions within 2.5 Å; 4 Å – interactions within 4 Å; cat-π – cationic π-interactions; HB – hydrogen bonds; π-π-stack – interactions involving π-π-stacking; SB – salt-bridge; T-stack – aromatic superposition (edge-face-interactions).

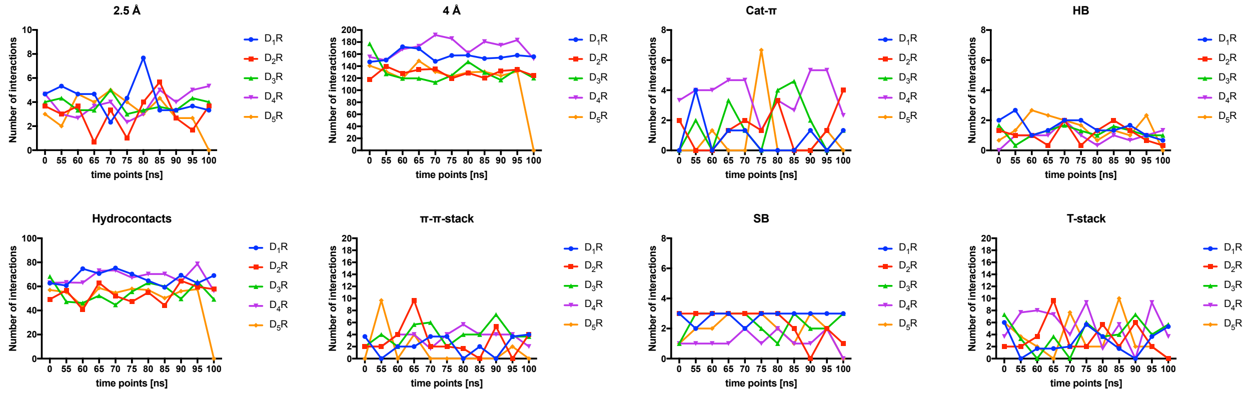

Figure S14 - Pairwise interactions results for nemonapride. Each type of interaction is displayed over all time points for each DR. The DR-subtypes are color-coded. Abbreviations: 2.5 Å – interactions within 2.5 Å; 4 Å – interactions within 4 Å; cat-π – cationic π-interactions; HB – hydrogen bonds; π-π-stack – interactions involving π-π-stacking; SB – salt-bridge; T-stack – aromatic superposition (edge-face-interactions).

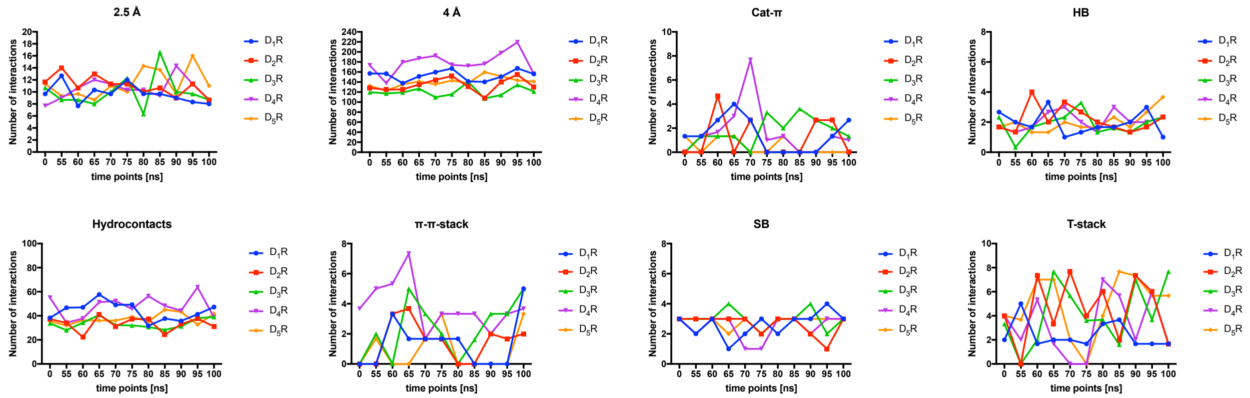

Figure S15 - Pairwise interactions results for sulpiride. Each type of interaction is displayed over all time points for each DR. The DR-subtypes are color-coded. Abbreviations: 2.5 Å – interactions within 2.5 Å; 4 Å – interactions within 4 Å; cat-π – cationic π-interactions; HB – hydrogen bonds; π-π-stack – interactions involving π-π-stacking; SB – salt-bridge; T-stack – aromatic superposition (edge-face-interactions).

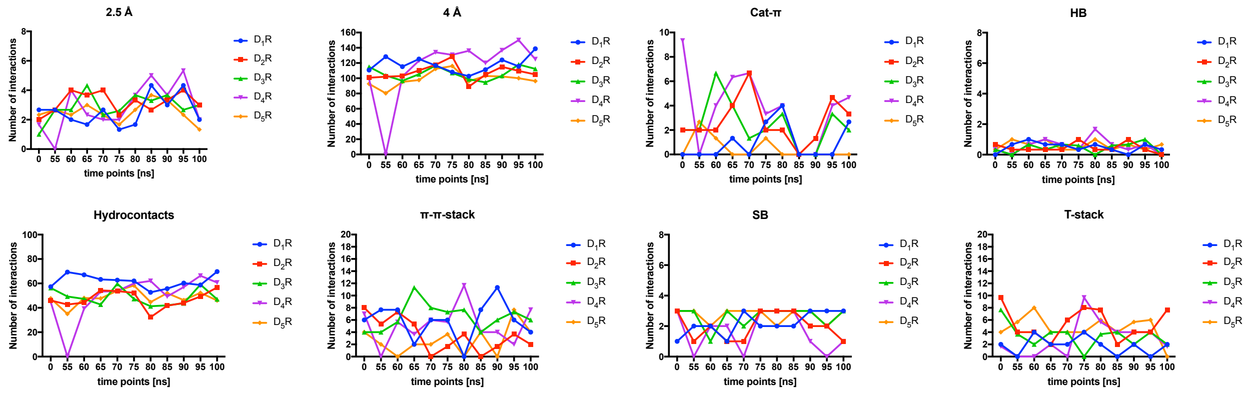

Figure S16 - Pairwise interactions results for SCH23390. Each type of interaction is displayed over all time points for each DR. The DR-subtypes are color-coded. Abbreviations: 2.5 Å – interactions within 2.5 Å; 4 Å – interactions within 4 Å; cat-π – cationic π-interactions; HB – hydrogen bonds; π-π-stack – interactions involving π-π-stacking; SB – salt-bridge; T-stack – aromatic superposition (edge-face-interactions).

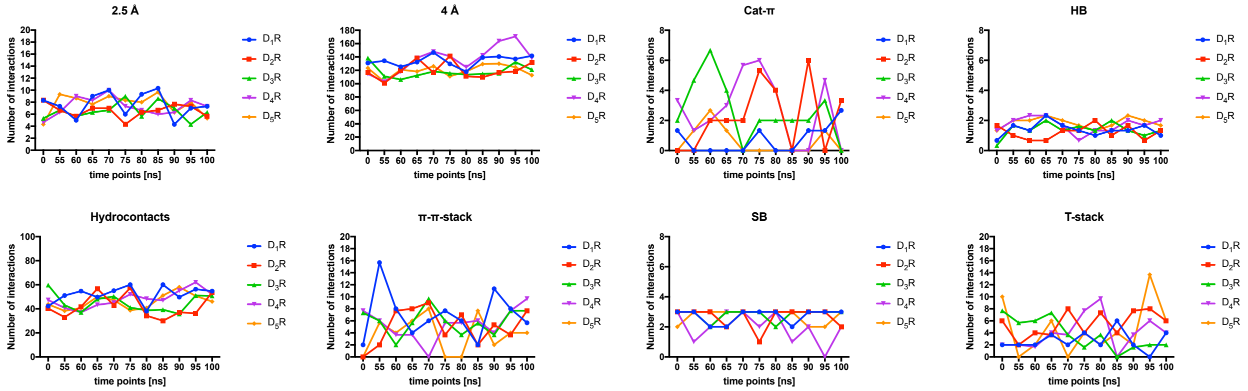

Figure S17 - Pairwise interactions results for SKF38393. Each type of interaction is displayed over all time points for each DR. The DR-subtypes are color-coded. Abbreviations: 2.5 Å – interactions within 2.5 Å; 4 Å – interactions within 4 Å; cat-π – cationic π-interactions; HB – hydrogen bonds; π-π-stack – interactions involving π-π-stacking; SB – salt-bridge; T-stack – aromatic superposition (edge-face-interactions).

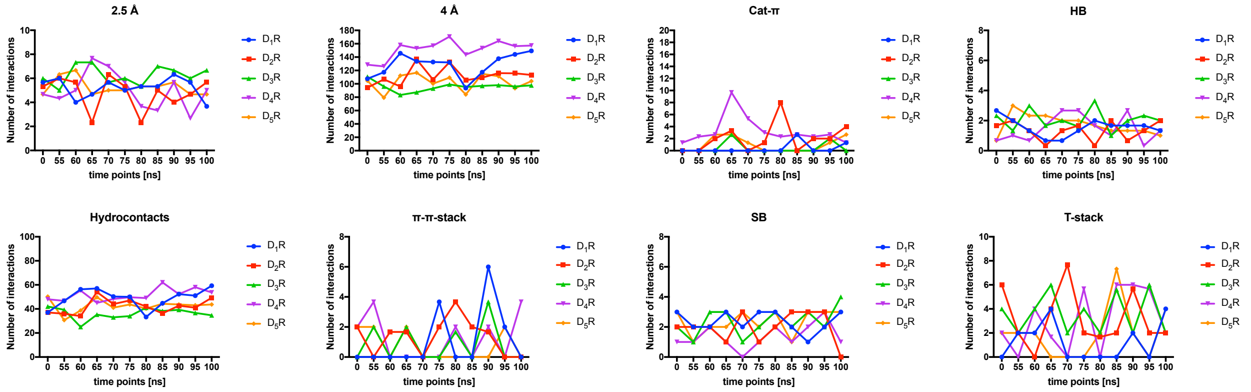

Figure S18 - Pairwise interactions results for eticlopride. Each type of interaction is displayed over all time points for each DR. The DR-subtypes are color-coded. Abbreviations: 2.5 Å – interactions within 2.5 Å; 4 Å – interactions within 4 Å; cat-π – cationic π-interactions; HB – hydrogen bonds; π-π-stack – interactions involving π-π-stacking; SB – salt-bridge; T-stack – aromatic superposition (edge-face-interactions).

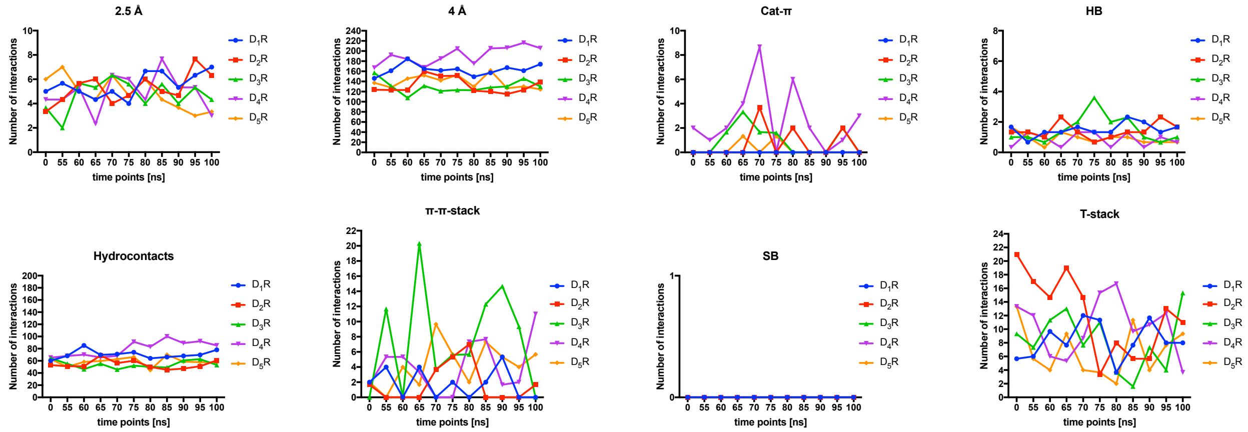

Figure S19 - Pairwise interactions results for risperidone. Each type of interaction is displayed over all time points for each DR. The DR-subtypes are color-coded. Abbreviations: 2.5 Å – interactions within 2.5 Å; 4 Å – interactions within 4 Å; cat-π – cationic π-interactions; HB – hydrogen bonds; π-π-stack – interactions involving π-π-stacking; SB – salt-bridge; T-stack – aromatic superposition (edge-face-interactions).

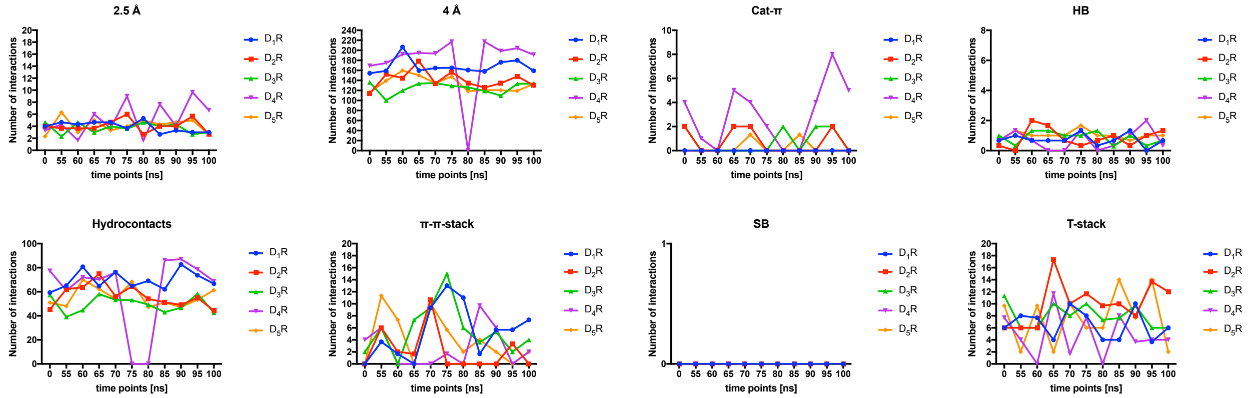

Figure S20 - Pairwise interactions results for aripiprazole. Each type of interaction is displayed over all time points for each DR. The DR-subtypes are color-coded. Abbreviations: 2.5 Å – interactions within 2.5 Å; 4 Å – interactions within 4 Å; cat-π – cationic π-interactions; HB – hydrogen bonds; π-π-stack – interactions involving π-π-stacking; SB – salt-bridge; T-stack – aromatic superposition (edge-face-interactions).

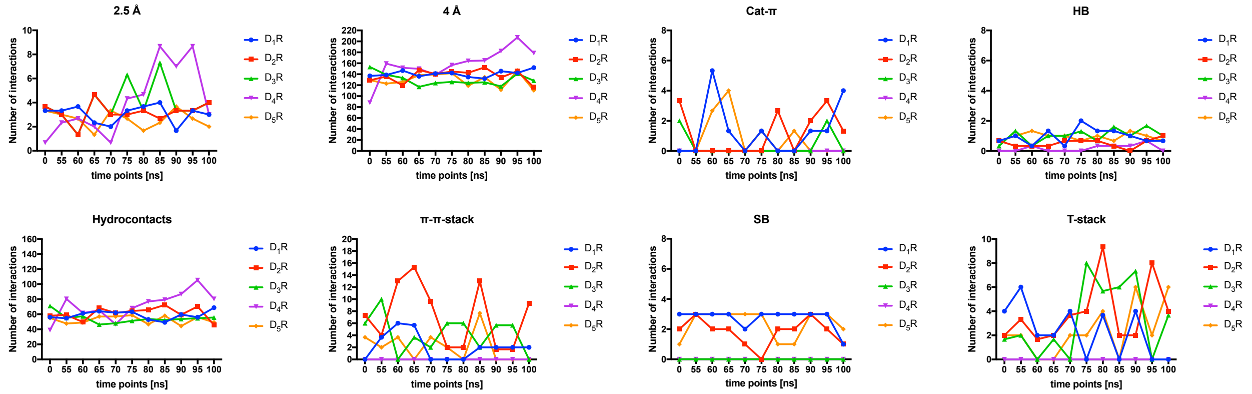

Figure S21 - Pairwise interactions results for haloperidole. Each type of interaction is displayed over all time points for each DR. The DR-subtypes are color-coded. Abbreviations: 2.5 Å – interactions within 2.5 Å; 4 Å – interactions within 4 Å; cat-π – cationic π-interactions; HB – hydrogen bonds; π-π-stack – interactions involving π-π-stacking; SB – salt-bridge; T-stack – aromatic superposition (edge-face-interactions).

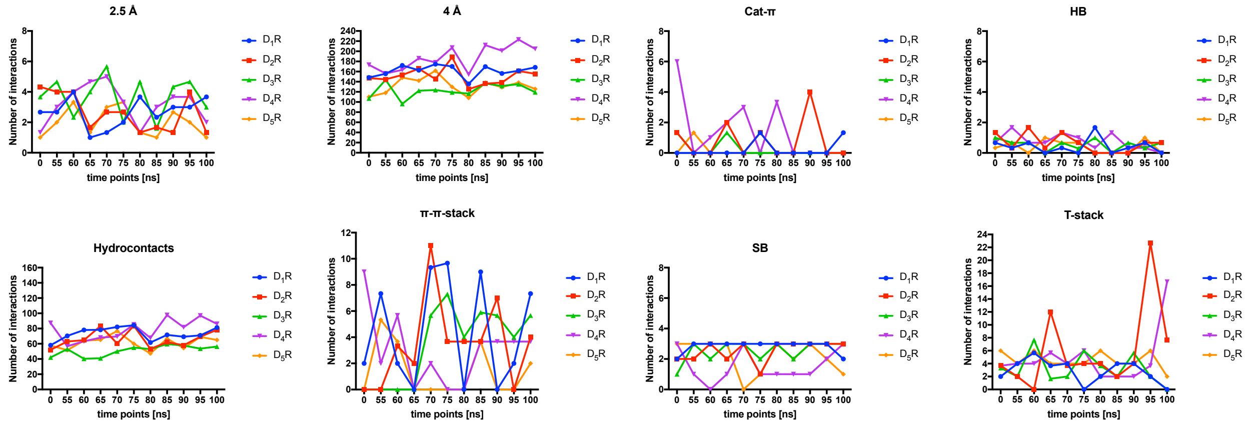

Figure S22 - Pairwise interactions results for spiperone. Each type of interaction is displayed over all time points for each DR. The DR-subtypes are color-coded. Abbreviations: 2.5 Å – interactions within 2.5 Å; 4 Å – interactions within 4 Å; cat-π – cationic π-interactions; HB – hydrogen bonds; π-π-stack – interactions involving π-π-stacking; SB – salt-bridge; T-stack – aromatic superposition (edge-face-interactions).

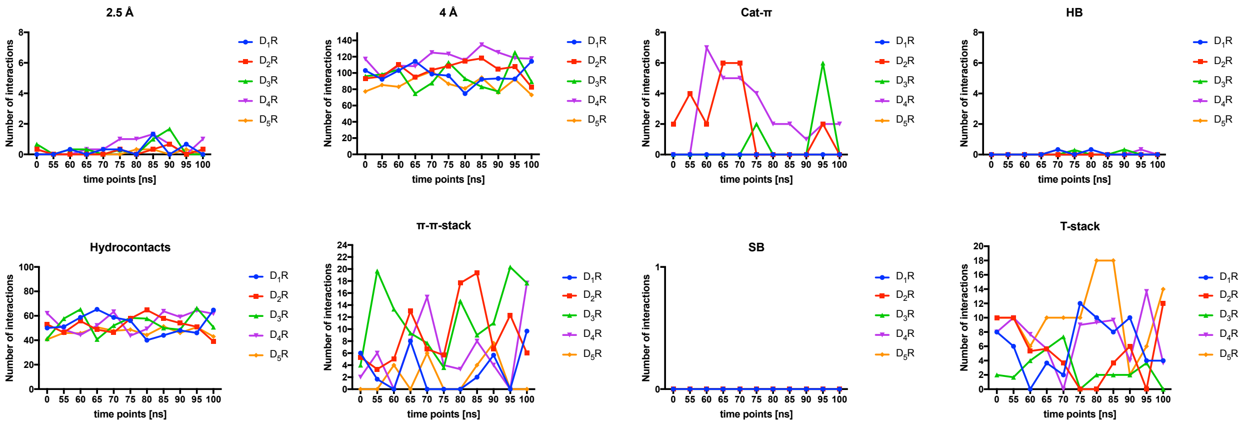

Figure S23 - Pairwise interactions results for chlorpromazine. Each type of interaction is displayed over all time points for each DR. The DR-subtypes are color-coded. Abbreviations: 2.5 Å – interactions within 2.5 Å; 4 Å – interactions within 4 Å; cat-π – cationic π-interactions; HB – hydrogen bonds; π-π-stack – interactions involving π-π-stacking; SB – salt-bridge; T-stack – aromatic superposition (edge-face-interactions).

TABLES

Table S1 - Comparison between the total and transmembrane specific identity [%] of the DR model with their crystal structure templates calculated with Clustal Omega.

	D_1R	D_2R	D_3R	D_4R	D_5R
Total similarity with the template [%]	39.5	100.0	99.3	100.0	39.1
TM1	44.0	100.0	100.0	100.0	21.4
TM2	60.0	100.0	100.0	100.0	54.8
TM3	51.5	96.7	96.7	100.0	52.9
TM4	23.1	100.0	100.0	100.0	26.1
TM5	36.1	92.3	100.0	97.6	29.7
TM6	34.5	94.6	100.0	100.0	29.7
TM7	37.5	100.0	100.0	100.0	40.0

Table S2 – Averages RMSD values for Cα atoms of TM throughout the simulations. Structure was fitted to the TM domains in study.

	TM1	TM2	TM3	TM4	TM5	TM6	TM7
D_1R	0.08 ± 0.01	0.09 ± 0.01	0.11 ± 0.01	0.07 ± 0.01	0.07 ± 0.01	0.07 ± 0.01	0.08 ± 0.01
D_2R	0.11 ± 0.02	0.10 ± 0.01	0.10 ± 0.01	0.10 ± 0.01	0.11 ± 0.01	0.14 ± 0.02	0.12 ± 0.01
D_3R	0.06 ± 0.01	0.05 ± 0.01	0.07 ± 0.01	0.08 ± 0.01	0.08 ± 0.01	0.08 ± 0.02	0.07 ± 0.01
D_4R	0.09 ± 0.02	0.07 ± 0.01	0.05 ± 0.01	0.07 ± 0.02	0.06 ± 0.01	0.05 ± 0.01	0.08 ± 0.02
D_5R	0.05 ± 0.01	0.07 ± 0.01	0.09 ± 0.01	0.10 ± 0.02	0.13 ± 0.02	0.10 ± 0.02	0.13 ± 0.03

Table S3 - Summary of the structures used in literature for defining the binding pocket for the D2R and source (experimental and computational) [2,39,44,47,49,51,99].

Reference	Ligands		Binding pocket (Experimental data)	Flexible residues (Computational data)
Tschammer *et al.*	Dopamine	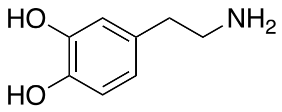	Asp86, Val87, Ser165, Ser169, Trp236, Phe239, Phe 240, His243	
	Haloperidole	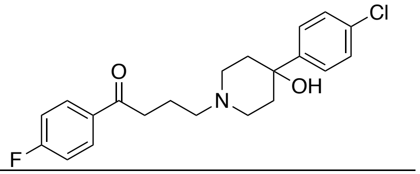		
	Nemonapride	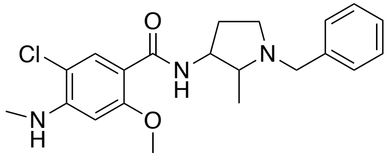		
	Spiperone	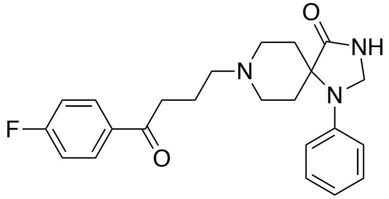		
	Quinpirole	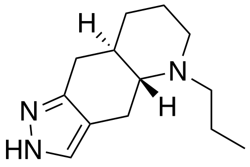		

	7-Hydroxy-*N*,*N*-dipropyl-2-aminotetralin (7-OH-DPAT)	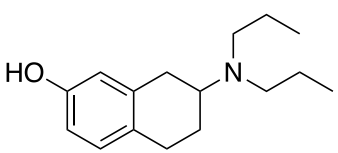	
	FAUC185	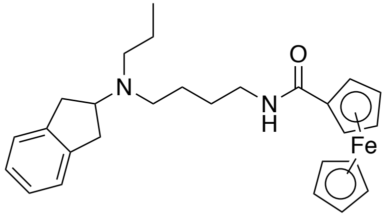	
	Dopamine	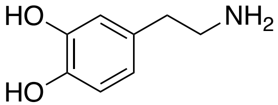	
	N-Dimethyldopamine	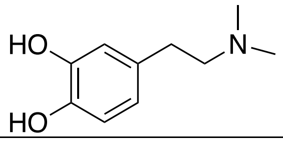	
Floresca *et al.*	N-Trimethyldopamine	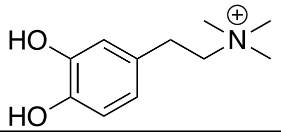	Ser165, Ser166, Ser169, Trp236, Phe239, Phe240, His243
	N-Dimethylselenodopamine	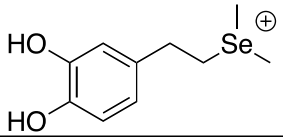	
	N-Dimethylsulfodopamine	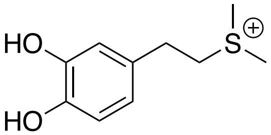	
	Monomethylsulfoniumdopamine	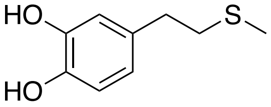	

Monomethylselemoniumdopamine	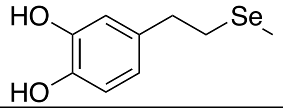
Monomethylsulfoxidedopamine	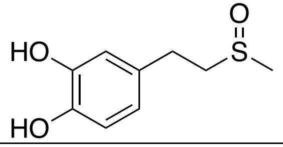
Metoclopramine (met-N(et)$_2$)	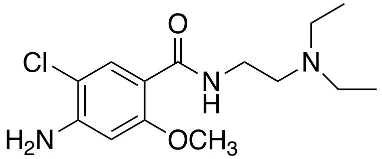
Met-N(me)$_2$	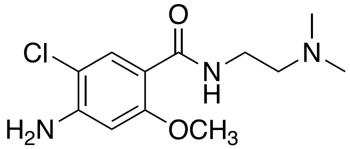
Met-N+(me)$_3$	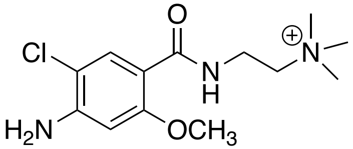
Met-N+(me)$_2$(et)	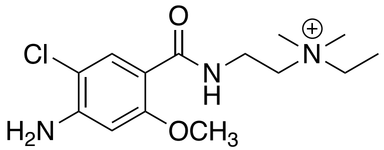
Met-N+(me)(et)$_2$	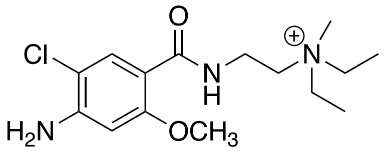

Met-S+(me)2	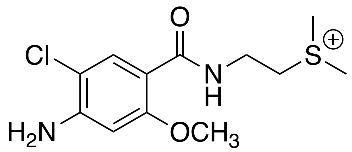
Sulpiride N-et	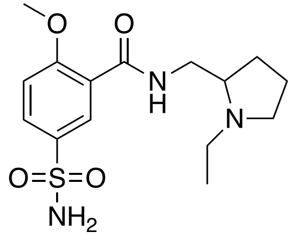
Sulpiride N+(et)(me)	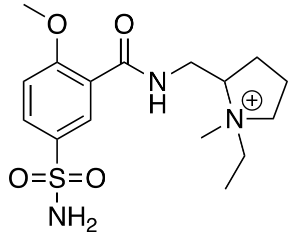
Sulpiride N+(et)2	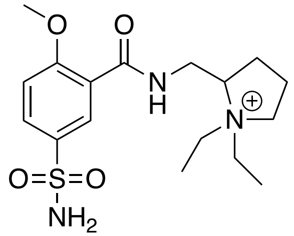
Sulpiride S+(et)	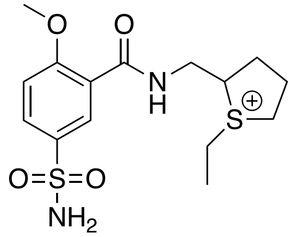

Sulpiride S	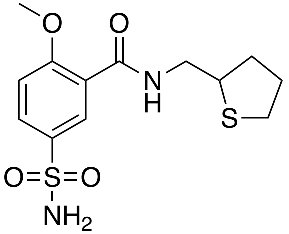
Chlorpromazine (CPZ-N(Me)$_2$)	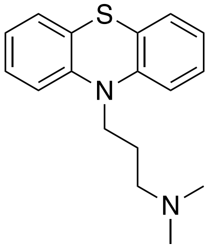
CPZ-N+(Me)$_3$	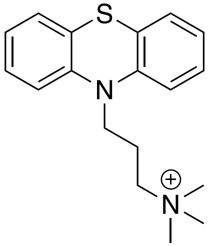
CPZ-S+(Me)$_2$	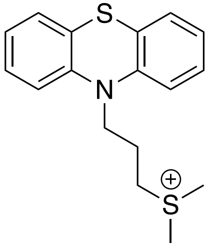
Beta-Phenylethylamine (PEA)	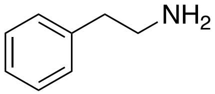

Meta-Tyramine	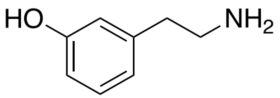
Para-Tyramine	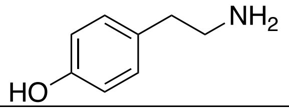
Apomorphine	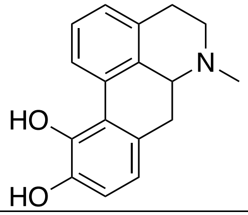
Norpropylapomorphine/ N-Proplapomorphine	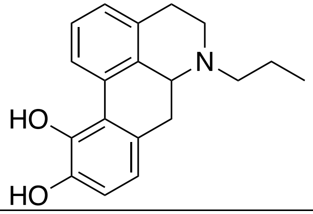
6,7-ADTN	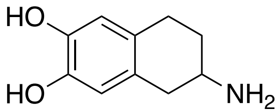
5,6-ADTN	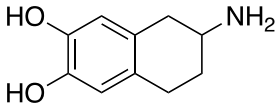
Quinpirole	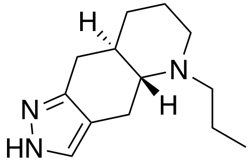

7-OH-DPAT	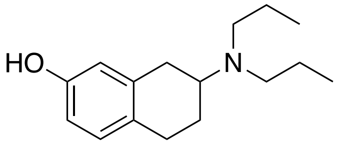
Spiperidone	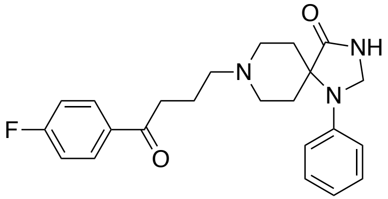
SKF38393	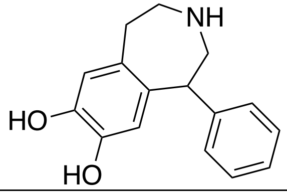
SKF82958	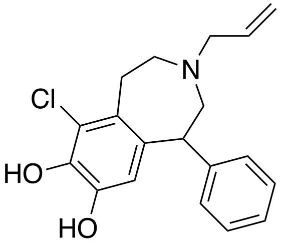
SCH23390	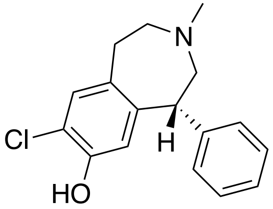

SCH23388	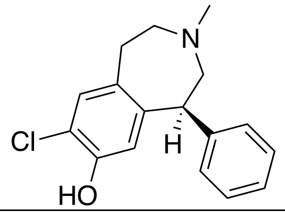
Haloperidole	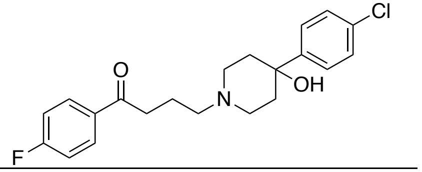
Remoxipride	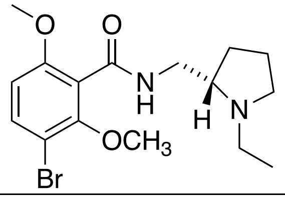
Epidipride	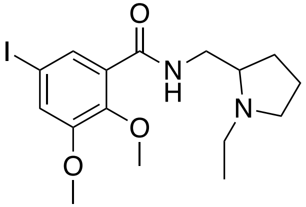
Raclopride	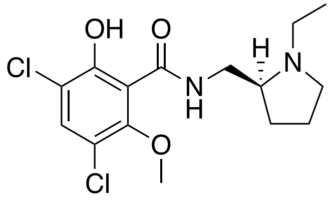

Domperidone	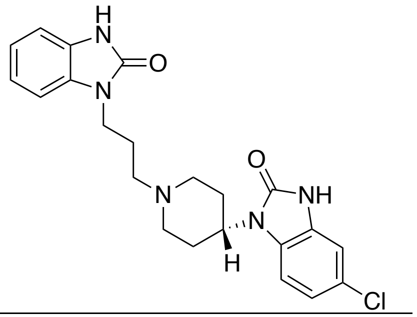
(+)-Butaclamol	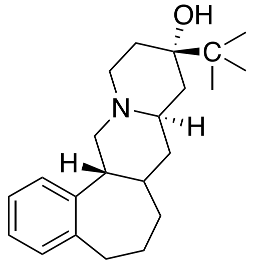
Clozapine	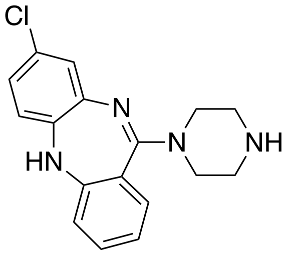
Olanzapine	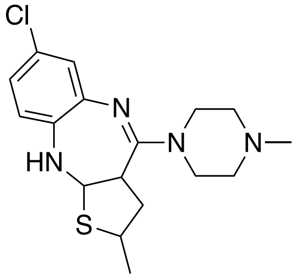

	Quetiapine	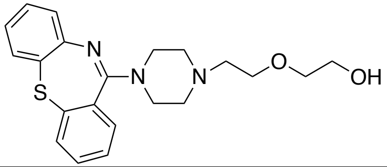		
	Ziprasidone	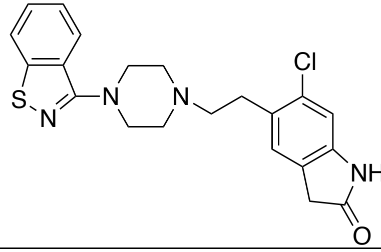		
	Risperidone	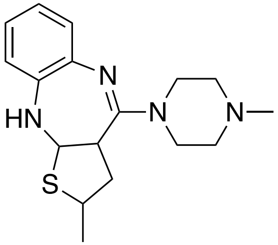		
Durdagi *et al.*	Dopamine	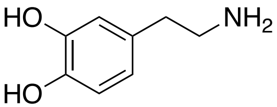	Asp86, Phe239, Phe240, Ser165, Ser166, Ser169, His243, Ile156, Trp236, Tyr258, Tyr266	<u>Glide/IFD:</u> Asp86, Trp236, Phe240, Tyr266<br><br><u>GOLD:</u><br>Asp86, Cys90, Ser165, Ser166, Ser169, Phe170, His243, Tyr258, Thr262, Tyr266
	Apomorphine	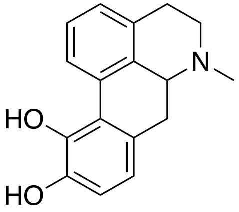		

Männel *et al.*	1,4 DAPs for compounds representing the "fragment"	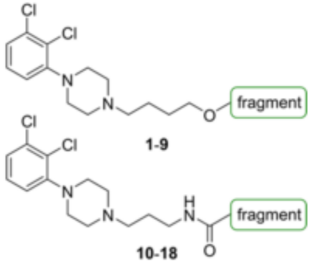	- Asp86 forming a salt-bridge<br>- Secondary binding pocket: Glu67, Ser259, Tyr258, Thr262, Tyr266
Kalani *et al.*	Apomorphine	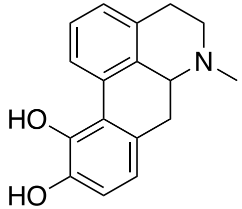	Asp86, Ser165, Ser169, Phe82, Met89, Cys90, Phe144, Phe170, Val172, Trp236, Phe240, His243
	Bromocriptine	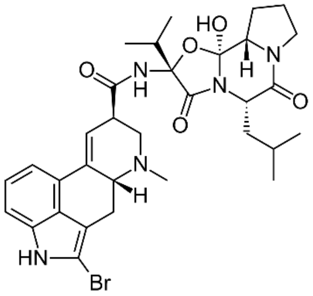	

Clozapine	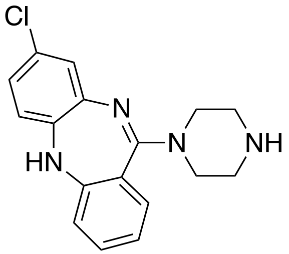
Domperidone	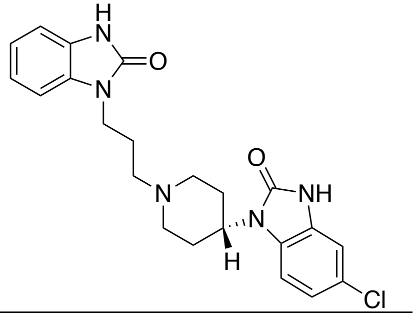
Dopamine	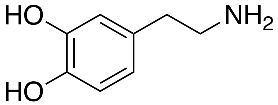
Haloperidole	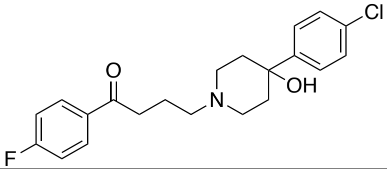
Raclopride	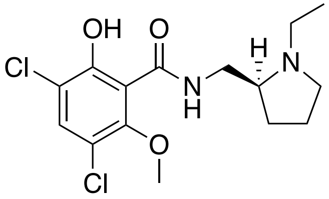

	7-OH-DPAT	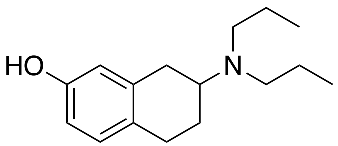		
	Spiperone	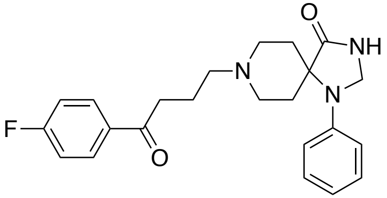		
	Sulpiride	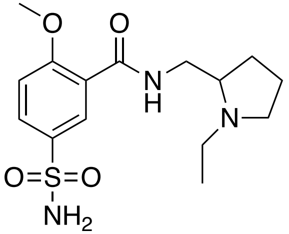		
Sukalovic *et al.*	Arylpiperazines<br>for compounds representing the residues R_1, R_2 and R_3	1) To construct the model<br>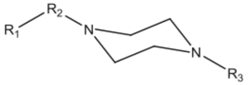<br>2) To test the model<br>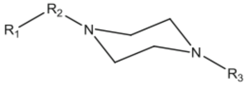<br>3) To probe the ECL2 area of the model<br>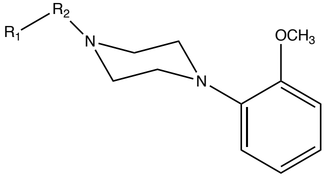	Asp86, Ser165, Ser169, Phe82, Met89, Cys90, Phe144, Phe170, Val172, Trp236, Phe240, His243	<u>Glide:</u><br>Asp86, Ser139, Ser165, Ser169, Phe232, Trp236, Tyr266

Table S4 - Docking results for the D1R. The lowest binding energy ΔGbind [kcal/mol] of every run was measured using AutoDock4.2. Calculated conformations were clustered and ranked by energy level. A populated cluster indicates, that the position docked into the receptor is more likely to depict the conformational binding position of the ligand. The three best clusters were chosen and analyzed. NA – Not Available, cases were the number of clusters was lower than 3.

Receptor state of the MD simulation [ns]	Top	0	55	60	65	70	75	80	85	90	95	100
Dopamine	Lowest binding energy [kcal/mol]											
	1	-10.71	-10.19	-9.29	-10.65	-9.92	-11.50	-10.67	-10.31	-10.96	-10.73	-9.35
	2	-9.56	-9.63	-8.64	-9.45	-9.42	-10.67	-9.81	-8.93	-10.80	-9.91	-9.21
	3	-8.61	-9.16	-7.48	-8.70	-8.18	-9.00	-7.82	-8.85	-10.04	-9.56	-7.97
	Number of conformations in cluster											
	1	64	13	12	18	49	45	68	74	61	19	15
	2	14	17	8	32	29	31	21	7	19	11	27
	3	1	4	5	11	4	5	1	4	2	43	1
7-OH-DPAT	Lowest binding energy [kcal/mol]											
	1	-9.33	-10.16	-8.39	-10.09	-9.08	-9.85	-9.27	-9.62	-9.98	-9.04	-9.78
	2	-8.19	-9.68	-8.33	-9.18	-8.38	-9.23	-9.00	-7.82	-9.46	-8.73	-9.07
	3	-8.09	-8.66	-7.97	-8.77	-7.79	-8.45	-7.59	-7.65	-8.91	-8.57	-8.25
	Number of conformations in cluster											
	1	46	13	10	2	13	24	37	55	44	13	10
	2	9	12	14	12	13	14	29	2	10	7	9
	3	18	5	18	2	3	8	5	6	3	9	2
Apomorphine	Lowest binding energy [kcal/mol]											
	1	-10.39	-10.47	-9.93	-10.79	-10.11	-11.39	-10.79	-10.09	-10.77	-10.44	-9.93
	2	-9.33	-8.87	-9.58	-10.08	-9.41	-11.17	-10.53	-9.99	-9.97	-9.88	-8.80
	3	-8.96	-8.82	-8.86	-9.00	-9.24	-9.60	-9.21	-8.64	-9.34	-8.95	-8.49
	Number of conformations in cluster											
	1	64	25	40	33	12	27	29	36	22	44	31
	2	12	11	26	21	37	32	34	20	16	14	6
	3	17	15	6	7	7	5	15	25	6	5	3
Bromocriptine	Lowest binding energy [kcal/mol]											

	1	+7.85	+2.32	+35.03	+15.82	+2.66	-5.33	-5.78	+26.43	+15.05	+35.22	+31.70
	2	+47.13	+10.47	+72.18	+16.34	+5.87	-4.82	-1.29	+49.89	+29.78	+40.32	+53.05
	3	+59.56	+22.09	+74.60	+40.56	+9.03	+1.52	-0.56	+54.28	+35.11	+52.86	+73.04
		Number of conformations in cluster										
	1	7	51	37	49	33	25	27	10	5	58	25
	2	21	28	22	22	35	28	12	5	46	37	35
	3	34	2	16	5	19	44	7	3	3	3	28
		Lowest binding energy [kcal/mol]										
	1	-8.09	-9.32	-9.47	-9.43	-8.49	-9.52	-8.89	-9.14	-9.05	-8.76	-8.33
	2	-7.23	-9.14	-8.87	-8.84	-8.33	-9.00	-8.41	-8.91	-8.39	-8.56	-7.18
Clozapine	3	-7.12	-8.94	-8.63	-8.24	-7.22	-8.28	-7.99	-8.09	NA	-8.48	-6.28
		Number of conformations in cluster										
	1	43	36	31	39	80	45	74	14	88	38	84
	2	54	1	50	52	18	2	9	38	12	17	14
	3	2	4	12	9	1	9	13	4	NA	40	1
		Lowest binding energy [kcal/mol]										
	1	-10.01	-10.84	-9.81	-11.15	-10.76	-11.02	-10.80	-10.73	-11.82	-11.08	-11.52
	2	-9.91	-10.24	-9.54	-10.90	-9.83	-10.77	-10.39	-10.26	-11.37	-10.79	-9,81
Nemonapride	3	-9.90	-10.00	-9.23	-10.79	-9.25	-10.70	-10.24	-10.00	-11.30	-10.66	-9.51
								Number of conformations in cluster				
	1	15	2	24	5	20	7	6	24	16	13	4
	2	22	15	11	4	16	1	6	13	17	20	2
	3	12	4	13	3	3	3	25	13	17	2	15
		Lowest binding energy [kcal/mol]										
	1	-11.43	-11.73	-8.76	-10.55	-12.51	-11.38	-10.88	-10.87	-11.64	-10.43	-10.95
	2	-11.24	-11.25	-7.61	-10.22	-11.16	-11.07	-10.68	-10.70	-10.30	-10.43	-10.76
Sulpiride	3	-10.73	-10.67	-7.36	-9.98	-8.85	-9.06	-10.13	-10.15	-10.01	-9.29	-10.35
		Number of conformations in cluster										
	1	4	14	5	8	11	8	12	10	6	3	7
	2	3	3	16	14	2	1	4	3	2	3	2
	3	3	2	5	2	3	5	1	2	5	2	4

SCH23390	Lowest binding energy [kcal/mol]											
	1	-9.31	-9.86	-9.33	-9.49	-9.78	-10.01	-9.05	-9.94	-10.01	-10.59	-9.45
	2	-8.44	-9.62	-9.21	-9.27	-9.51	-9.86	-9.03	-9.72	-9.71	-9.62	-8.73
	3	-8.16	-9.15	-9.02	-9.23	-8.71	-9.84	-8.54	-9.09	-9.69	-9.17	-8.68
	Number of conformations in cluster											
	1	13	2	30	41	39	16	15	4	11	18	5
	2	3	6	29	5	6	5	17	4	5	6	3
	3	1	11	5	6	2	1	3	1	6	3	4
SKF38393	Lowest binding energy [kcal/mol]											
	1	-12.23	-11.73	-8.91	-10.11	-11.44	-13.06	-11.87	-12.18	-12.50	-12.00	-11.88
	2	-11.00	-9.15	-8.58	-9.45	-9.25	-11.90	-11.32	-10.37	-11.35	-11.09	-9.41
	3	-9.40	-9.13	-8.36	-9.05	-9.05	-8.74	-9.55	-8.97	-9.45	-10.99	-9.26
	Number of conformations in cluster											
	1	38	3	8	6	36	56	32	29	50	16	26
	2	22	6	7	6	1	8	19	17	3	30	4
	3	4	3	9	12	5	3	1	1	9	2	20
Eticlopride	Lowest binding energy [kcal/mol]											
	1	-9.65	-10.53	-9.39	-10.99	-9.85	-10.47	-10.06	-10.61	-9.67	-10.15	-9.75
	2	-9.57	-9.56	-8.69	-10.52	-9.26	-10.34	-8.88	-9.61	-9.61	-9.82	-9.66
	3	-8.43	-9.37	-8.44	-9.73	-9.19	-10.04	-8.82	-9.51	-9.14	-9.18	-9.29
	Number of conformations in cluster											
	1	12	13	10	7	12	2	6	16	16	25	4
	2	2	7	14	29	9	6	3	14	3	23	4
	3	7	8	19	7	1	14	25	3	2	12	14
Risperidone	Lowest binding energy [kcal/mol]											
	1	-9.17	-10.19	-7.51	-9.12	-8.50	-9.96	-9.60	-10.35	-9.72	-11.60	-9.66
	2	-8.29	-9.42	-6.36	-8.82	-8.16	-8.98	-8.62	-9.11	-9.40	-10.48	-8.71
	3	-7.62	-8.90	-5.11	-7.91	-7.95	-8.02	-8.40	-7.08	-8.78	-9.31	-8.70
	Number of conformations in cluster											
	1	9	31	6	30	32	32	40	42	31	10	8
	2	27	20	25	19	4	18	3	29	8	28	24

	3	6	5	15	3	7	8	4	3	4	7	6
Aripiprazole	Lowest binding energy [kcal/mol]											
	1	-10.26	-11.59	-10.11	-11.79	-10.73	-9.99	-10.51	-10.29	-11.12	-9.85	-10.68
	2	-9.74	-11.24	-8.93	-10.64	-10.02	-9.31	-9.86	-10.06	-10.08	-8.72	-9.95
	3	-9.64	-10.28	-8.17	-9.43	-9.37	-9.20	-9.63	-9.67	-9.96	-8.43	-9.94
	Number of conformations in cluster											
	1	8	7	10	5	14	12	6	19	16	29	5
	2	1	3	5	18	1	6	8	1	9	14	9
	3	8	11	11	1	2	2	1	7	3	4	16
Haloperidole	Lowest binding energy [kcal/mol]											
	1	-9.84	-11.35	-10.73	-11.68	-10.58	-10.65	-10.55	-9.87	-11.19	-10,93	-10.47
	2	-9.81	-10.99	-9.68	-10.87	-10.21	-10.50	-10.49	-9.52	-10.08	-10.58	-10.15
	3	-9.33	-10.91	-9.64	-10.61	-10.10	-10.50	-9.73	-9.51	-9.90	-10.51	-9.60
	Number of conformations in cluster											
	1	28	20	42	4	37	4	9	27	32	4	7
	2	6	22	7	31	2	9	5	4	13	5	5
	3	1	9	10	4	8	16	7	3	9	9	7
Spiperone	Lowest binding energy [kcal/mol]											
	1	-9.70	-11.12	-9.30	-10.93	-11.09	-10.37	-10.76	-10.56	-10.76	-10.81	-10.35
	2	-9.31	-10.95	-9.23	-10.46	-9.99	-10.03	-9.97	-10.33	-10.71	-10.10	-9.93
	3	-9.02	-10.56	-8.99	-10.05	-9.81	-9.75	-9.81	-9.50	-10.48	-9.77	-9.63
	Number of conformations in cluster											
	1	29	9	8	13	2	1	4	2	11	26	7
	2	14	11	10	7	4	3	8	35	8	4	7
	3	5	4	17	13	9	12	3	10	16	6	10
Chlorpromazine	Lowest binding energy [kcal/mol]											
	1	-8.10	-8.70	-9.19	-9.04	-8.92	-8.93	-8.02	-8.06	-8.68	-8.91	-8.82
	2	-7.98	-8.55	-9.00	-8.87	-8.39	-8.70	-7.94	-8.01	-8.67	-8.75	-8.51
	3	-7.92	-8.52	-8.95	-8.79	-8.35	-8.59	-7.92	-8.00	-8.63	-8.72	-8.43
	Number of conformations in cluster											
	1	16	24	14	17	25	24	12	13	5	23	1

	2	9	30	22	2	13	24	22	17	4	26	17
	3	21	4	12	2	4	9	17	8	35	9	19

Table S5 - Docking results for the D$_2$R. The lowest binding energy ΔG_{bind} [kcal/mol] of every run was measured using AutoDock4.2. Calculated conformations were clustered and ranked by energy level. A populated cluster indicates, that the position docked into the receptor is more likely to depict the conformational binding position of the ligand. The three best clusters were chosen and analyzed.

Receptor state of the MD simulation [ns]	Top	0	55	60	65	70	75	80	85	90	95	100
Dopamine		Lowest binding energy [kcal/mol]										
	1	-9.72	-9.76	-9.69	-9.29	-9.11	-9.57	-9.37	-8.93	-9.15	-10.31	-9.78
	2	-9.71	-9.15	-9.27	-9.02	-9.02	-9.05	-8.98	-8.82	-8.89	-9.91	-9.04
	3	-8.61	-8.64	-8.30	-8.44	-8.98	-8.91	-8.73	-8.71	-8.76	-8.91	-8.60
		Number of conformations in cluster										
	1	32	24	19	16	24	23	33	3	15	6	12
	2	29	17	19	32	4	18	20	31	8	26	19
	3	6	20	15	11	8	4	10	10	22	29	13
7-OH-DPAT		Lowest binding energy [kcal/mol]										
	1	-9.26	-8.16	-8.30	-9.02	-8.46	-9.26	-8.59	-8.08	-8.92	-8.37	-9.30
	2	-8.28	-7.99	-7.85	-8.72	-8.34	-8.34	-8.31	-7.33	-7.76	-8.25	-8.31
	3	-8.06	-7.98	-7.73	-8.59	-8.25	-7.93	-7.89	-7.06	-7.50	-7.86	-8.21
		Number of conformations in cluster										
	1	26	8	11	2	17	43	20	8	19	20	21
	2	17	17	10	14	15	6	5	41	13	4	2
	3	14	28	1	6	6	9	5	4	15	16	2
Apomorphine		Lowest binding energy [kcal/mol]										
	1	-10.05	-10.21	-10.28	-11.38	-11.09	-11.10	-10.03	-10.31	-10.61	-10.82	-10.44
	2	-9.62	-10.04	-9.88	-11.31	-10.03	-10.22	-9.97	-9.71	-10.35	-9.69	-10.10
	3	-9.60	-9.76	-9.41	-10.72	-9.68	-9.78	-9.36	-9.15	-9.51	-9.51	-9.82

	1	34	16	36	2	5	45	31	14	19	14	12
	2	22	11	10	13	21	15	21	3	4	16	25
	3	25	21	4	7	17	1	7	23	3	23	3
Bromocriptine	Lowest binding energy [kcal/mol]											
	1	-3.47	-7.61	+2.18	+14.69	-2.36	+20.66	-5.43	-6.76	-6.36	-0.24	-3.38
	2	+5.75	-1.33	+4.49	+15.35	+2.84	+23.11	-5.26	-5.82	-5.19	+1.49	+6.48
	3	+6.87	+5.01	+13.20	+17.48	+3.32	+32.17	-4.99	-4.88	+0.45	+2.79	+6.62
	Number of conformations in cluster											
	1	13	79	9	14	21	24	7	42	24	25	36
	2	12	2	19	22	16	3	24	7	7	12	2
	3	6	1	8	42	22	1	25	13	3	3	29
Clozapine	Lowest binding energy [kcal/mol]											
	1	-8.10	-9.20	-8.50	-9.39	-8.66	-8.54	-8.71	-7.44	-8.88	-8.75	-8.19
	2	-7.59	-8.12	-8.14	-8.81	-8.56	-8.54	-8.68	-6.70	-8.28	-8.45	-7.96
	3	-7.42	-8.09	-7.47	-8.34	-8.34	-8.44	-8.24	-6.00	-8.25	-8.16	-7.37
	Number of conformations in cluster											
	1	21	66	18	19	26	41	14	27	3	2	1
	2	2	9	40	56	7	4	13	7	2	1	55
	3	55	1	3	3	11	45	18	6	7	5	13
Nemonapride	Lowest binding energy [kcal/mol]											
	1	-10.10	-10.56	-11.39	-10.08	-10.58	-10.32	-11.11	-10.72	-10.23	-10.45	-11.86
	2	-9.43	-9.92	-10.04	-9.79	-10.30	-9.88	-10.64	-10.60	-10.00	-10.35	-11.17
	3	-9.34	-9.60	-9.79	-9.73	-10.30	-9.80	-9.98	-10.43	-9.97	-10.08	-10.62
	Number of conformations in cluster											
	1	9	3	5	7	6	9	7	5	2	17	5
	2	7	5	2	18	3	8	15	28	3	5	1
	3	7	19	16	7	20	3	2	3	6	1	4
Sulpiride	Lowest binding energy [kcal/mol]											
	1	-11.39	-11.20	-11.35	-12.39	-11.39	-11.63	-11.90	-9.41	-10.98	-11.51	-10.44
	2	-10.43	-10.74	-10.95	-12.10	-10.25	-11.22	-11.20	-9.09	-10.18	-11.48	-10.16
	3	-10.23	-10.44	-10.41	-11.72	-10.11	-10.90	-11.07	-9.06	-10.14	-10.94	-10.11

	Number of conformations in cluster											
	1	7	1	2	4	1	5	3	4	3	1	9
	2	7	5	2	2	1	1	2	1	3	2	1
	3	2	10	1	4	5	3	2	7	2	1	8
SCH23390	Lowest binding energy [kcal/mol]											
	1	-9.60	-9.06	-10.05	-9.93	-9.91	-9.49	-8.83	-8.50	-9.98	-9.87	-10.17
	2	-9.36	-8.89	-9.31	-9.81	-9.89	-9.39	-8.75	-8.34	-8.89	-9.74	-9.86
	3	-8.81	-8.50	-8.80	-9.07	-9.76	-9.28	-8.58	-8.17	-8.77	-9.39	-9.73
	Number of conformations in cluster											
	1	10	10	1	24	1	10	16	53	4	4	8
	2	4	4	17	3	12	12	11	5	5	2	18
	3	4	2	3	6	31	2	19	1	10	6	1
SKF38393	Lowest binding energy [kcal/mol]											
	1	-11.35	-11.98	-10.73	-12.33	-12.41	-10.38	-10.35	-9.36	-11.13	-11.02	-10.81
	2	-10.04	-10.48	-10.07	-11.26	-10.12	-9.74	-10.18	-8.89	-9.99	-10.34	-10.79
	3	-9.85	-10.29	-10.06	-9.92	-9.90	-9.62	-9.75	-8.63	-9.96	-10.04	-10.00
	Number of conformations in cluster											
	1	15	14	16	5	55	45	25	29	23	19	8
	2	25	24	14	6	1	4	6	2	11	6	2
	3	1	17	6	4	7	1	27	42	9	4	2
Eticlopride	Lowest binding energy [kcal/mol]											
	1	-9.33	-9.52	-9.37	-9.87	-10.44	-10.08	-9.19	-8.67	-10.61	-9.88	-9.36
	2	-9.22	-9.31	-9.22	-9.62	-9.95	-9.93	-9.04	-8.66	-9.61	-9.01	-9.20
	3	-9.10	-9.30	-9.02	-9.54	-9.53	-8.89	-9.00	-8.04	-8.59	-8.82	-8.88
	Number of conformations in cluster											
	1	12	15	10	18	6	1	6	2	12	4	1
	2	6	9	5	1	10	22	3	10	5	4	9
	3	2	2	1	4	11	11	6	23	3	6	15
Risperidone	Lowest binding energy [kcal/mol]											
	1	-10.94	-11.24	-11.72	-14.83	-11.91	-11.78	-11.83	-10.98	-11.10	-10.54	-12.61
	2	-10.66	-10.59	-10.69	-12.46	-11.74	-10.83	-11.07	-10.73	-10.65	-10.39	-11.20

	3	-10.63	-9.80	-10.59	-11.56	-11.71	-10.66	-10.52	-10.63	-10.16	-10.03	-10.86
	Number of conformations in cluster											
	1	9	9	18	8	6	14	11	14	10	1	3
	2	2	38	4	17	1	7	2	6	7	2	6
	3	11	10	11	13	8	2	8	2	2	2	2
Aripiprazole	Lowest binding energy [kcal/mol]											
	1	-10.77	-11.08	-10.87	-10.87	-11.17	-10.54	-10.27	-9.34	-9.96	-10.95	-10.74
	2	-10.60	-10.51	-9.85	-10.76	-10.70	-10.10	-10.13	-8.29	-9.50	-9.31	-10.05
	3	-10.30	-10.50	-9.48	-10.59	-10.66	-9.68	-9.65	-8.12	-9.25	-9.01	-9.52
	Number of conformations in cluster											
	1	12	7	1	5	4	24	2	1	5	3	3
	2	3	8	3	2	2	3	3	6	3	2	18
	3	3	2	1	6	3	3	3	1	1	2	2
Haloperidole	Lowest binding energy [kcal/mol]											
	1	-10.66	-10.84	-10.84	-12.69	-11.13	-12.52	-12.06	-10.62	-10.23	-11.73	-12.02
	2	-10.41	-10.03	-10.06	-12.09	-11.10	-11.23	-11.21	-10.19	-10.23	-11.45	-11.69
	3	-10.39	-9.98	-9.95	-11.82	-10.53	-10.75	-11.08	-10.12	-10.18	-11.32	-10.52
	Number of conformations in cluster											
	1	6	20	7	5	7	14	7	8	7	8	11
	2	3	1	7	6	3	6	1	3	1	4	6
	3	8	1	2	11	6	3	4	2	2	2	2
Spiperone	Lowest binding energy [kcal/mol]											
	1	-10.39	-11.13	-10.43	-11.75	-10.41	-10.56	-10.61	-8.66	-10.86	-10.09	-10.86
	2	-10.17	-10.30	-10.17	-10.95	-10.37	-10.54	-10.41	-8.45	-9.61	-10.01	-10.06
	3	-10.02	-9.85	-10.00	-10.37	-10.22	-10.25	-9.80	-8.35	-9.55	-9.81	-9.83
	Number of conformations in cluster											
	1	23	1	3	4	4	2	11	1	4	3	8
	2	7	2	5	6	1	22	4	9	12	4	5
	3	7	1	5	6	1	1	4	6	2	3	1
Chlorpromazine	Lowest binding energy [kcal/mol]											
	1	-8.01	-8.13	-8.28	-8.91	-8.93	-9.00	-9.34	-8.91	-8.28	-8.44	-8.39

	2	-7.91	-7.96	-8.03	-8.88	-8.40	-8.97	-8.42	-8.14	-8.19	-8.35	-8.38
	3	-7.76	-7.93	-7.97	-8.77	-8.39	-8.87	-8.01	-7.86	-8.08	-8.17	-8.32
		Number of conformations in cluster										
	1	2	16	9	6	13	20	2	1	1	1	8
	2	24	16	2	7	2	25	1	3	14	1	8
	3	3	1	11	24	8	2	2	1	2	2	9

Table S6 - Docking results for the D3R. The lowest binding energy ΔG_{bind} [kcal/mol] of every run was measured using AutoDock4.2. Calculated conformations were clustered and ranked by energy level. A populated cluster indicates, that the position docked into the receptor is more likely to depict the conformational binding position of the ligand. The three best clusters were chosen and analyzed.

Receptor state of the MD simulation [ns]	Top	0	55	60	65	70	75	80	85	90	95	100
Dopamine		Lowest binding energy [kcal/mol]										
	1	-9.76	-9.63	-8.92	-9.34	-8.97	-8.75	-9.88	-9.87	-9.77	-9.51	-8.86
	2	-9.55	-9.48	-8.67	-8.79	-8.95	-8.72	-9.59	-8.81	-9.00	-8.79	-8.72
	3	-8.64	-8.64	-8.49	-8.34	-8.27	-8.18	-8.82	-8.26	-8.18	-7.96	-8.07
		Number of conformations in cluster										
	1	17	42	14	34	23	35	69	56	40	43	8
	2	47	17	22	11	27	12	5	6	7	11	29
	3	18	16	2	15	10	11	12	6	12	6	12
7-OH-DPAT		Lowest binding energy [kcal/mol]										
	1	-9.11	-8.35	-8.43	-9.43	-8.37	-9.47	-9.75	-8.20	-8.36	-9.38	-8.57
	2	-8.89	-7.99	-7.56	-7.67	-7.70	-8.53	-8.93	-8.02	-8.07	-8.91	-8.21
	3	-8.52	-7.60	-7.53	-7.61	-7.54	-8.29	-7.92	-7.55	-7.87	-7.83	-7.85
		Number of conformations in cluster										
	1	5	46	35	29	39	20	28	35	41	13	8
	2	51	4	34	3	12	7	20	12	5	3	9
	3	1	12	1	5	4	5	3	1	3	4	8
Apomorphine		Lowest binding energy [kcal/mol]										
	1	-9.89	-10.19	-9.40	-10.18	-9.28	-9.51	-10.52	-9.91	-10.11	-9.40	-9.87

	2	-9.81	-9.46	-9.02	-9.34	-8.93	-9.33	-9.64	-9.46	-9.68	-9.17	-9.28
	3	-9.81	-8.51	-8.45	-8.75	-8.75	-8.97	-9.42	-9.26	-9.18	-9.12	-9.13
	Number of conformations in cluster											
	1	36	2	28	22	18	1	3	9	27	10	23
	2	22	38	25	33	22	11	31	22	13	31	19
	3	14	21	10	2	2	15	16	3	23	24	2
Bromocriptine	Lowest binding energy [kcal/mol]											
	1	-2.28	+29.67	+33.62	+10.12	+23.21	+10.12	+19.76	+1.48	+11.26	+42.39	+4.31
	2	+13.56	+81.44	+34.66	+11.36	+24.67	+10.32	+34.46	+10.88	+20.50	+61.90	+23.94
	3	+16.35	+100.91	+36.66	+22.89	+28.29	+15.51	+52.78	+26.06	+23.50	+80.64	+43.10
	Number of conformations in cluster											
	1	73	5	3	18	2	13	2	27	17	19	34
	2	4	8	4	12	5	22	2	27	6	9	2
	3	1	1	2	7	11	3	27	1	7	20	3
Clozapine	Lowest binding energy [kcal/mol]											
	1	-8.48	-7.16	-8.72	-8.12	-8.36	-8.16	-9.15	-8.33	-8.49	-8.37	-8.51
	2	-8.43	-7.12	-7.23	-7.27	-7.47	-7.69	-7.90	-7.29	-8.08	-8.37	-8.46
	3	-8.20	-6.95	-7.22	-7.26	-7.38	-7.65	-7.85	-7.24	-7.85	-8.19	-7.90
	Number of conformations in cluster											
	1	13	6	17	14	25	9	40	25	2	49	21
	2	7	38	11	24	14	1	12	31	6	17	7
	3	8	49	8	32	12	7	7	4	36	17	3
Nemonapride	Lowest binding energy [kcal/mol]											
	1	-10.97	-10.63	-9.12	-10.00	-9.91	-9.85	-10.17	-9.89	-9.94	-10.09	-10.01
	2	-10.86	-10.23	-8.92	-9.85	-9.48	-9.53	-9.82	-9.66	-9.59	-10.00	-9.71
	3	-10.26	-9.96	-8.68	-9.40	-9.12	-9.45	-9.71	-9.59	-9.32	-9.90	-9.55
	Number of conformations in cluster											
	1	2	4	1	1	12	1	2	8	5	1	5
	2	3	1	4	3	3	5	2	6	2	19	2
	3	1	2	2	2	1	2	2	1	4	3	6
Sulpiride	Lowest binding energy [kcal/mol]											

	1	-10.55	-9.69	-9.77	-8.93	-9.30	-9.94	-10.24	-10.01	-9.73	-9.99	-10.55
	2	-10.25	-8.90	-9.41	-8.91	-8.86	-9.84	-9.63	-9.78	-8.85	-9.46	-9.81
	3	-9.59	-8.89	-8.44	-8.85	-8.70	-9.68	-9.34	-9.71	-8.55	-9.17	-9.79
		Number of conformations in cluster										
	1	12	1	1	2	3	2	4	2	4	1	3
	2	6	7	1	1	3	2	4	2	2	19	1
	3	7	1	4	2	3	3	3	2	1	7	12
SCH23390		Lowest binding energy [kcal/mol]										
	1	-10.59	-9.38	-8.64	-9.96	-10.13	-9.76	-10.34	-9.59	-10.39	-10.71	-9.72
	2	-9.69	-9.07	-8.33	-9.65	-9.72	-9.19	-9.64	-9.16	-9.81	-10.00	-9.68
	3	-9.56	-8.70	-8.21	-8.98	-9.47	-9.03	-9.44	-8.64	-8.71	-9.71	-9.41
		Number of conformations in cluster										
	1	11	30	10	3	31	1	11	5	9	28	2
	2	2	12	1	9	5	8	30	20	30	9	23
	3	6	10	28	30	2	5	19	39	36	2	2
SKF38393		Lowest binding energy [kcal/mol]										
	1	-11.68	-10.68	-9.36	-11.04	-11.53	-10.47	-11.15	-10.41	-10.85	-10.69	-10.08
	2	-11.34	-10.28	-9.33	-10.88	-10.24	-10.30	-10.69	-10.35	-10.69	-10.24	-9.74
	3	-11.08	-10.23	-9.25	-10.83	-10.15	-9.66	-9.95	-9.87	-10.07	-9.78	-9.63
		Number of conformations in cluster										
	1	1	20	30	21	12	23	16	58	31	9	26
	2	36	9	6	5	8	22	56	5	20	1	2
	3	2	32	4	23	8	1	2	2	8	7	2
Eticlopride		Lowest binding energy [kcal/mol]										
	1	-9.98	-8.71	-7.99	-9.04	-9.45	-9.36	-9.58	-9.19	-9.41	-9.85	-9.23
	2	-9.34	-8.69	-7.95	-8.90	-9.02	-8.81	-9.10	-8.99	-9.37	-9.47	-9.17
	3	-9.07	-8.64	-7.88	-8.80	-8.76	-8.79	-8.83	-8.92	-9.20	-9.43	-9.03
		Number of conformations in cluster										
	1	14	31	7	9	7	5	1	9	2	7	6
	2	2	5	2	12	8	5	10	12	15	27	5
	3	13	2	2	12	2	17	2	9	8	17	3

Risperidone	Lowest binding energy [kcal/mol]										
	1	-8.87	-8.09	-7.91	-8.88	-8.67	-9.59	-9.44	-9.46	-9.51	-9.11	-9.72
	2	-8.23	-7.76	-7.77	-8.71	-7.89	-9.43	-9.14	-9.06	-9.48	-9.05	-9.47
	3	-8.17	-7.73	-7.73	-8.61	-7.87	-9.07	-9.09	-8.93	-9.37	-9.03	-8.86
	Number of conformations in cluster										
	1	16	1	6	2	12	7	2	15	12	7	5
	2	5	23	3	1	4	12	4	1	1	5	4
	3	2	2	3	4	1	4	9	15	7	10	11
Aripiprazole	Lowest binding energy [kcal/mol]										
	1	-10.40	-8.59	-8.89	-8.97	-9.79	-10.13	-10.37	-9.24	-9.74	-10.34	-10.44
	2	-10.27	-8.24	-8.66	-8.96	-9.25	-9.33	-10.30	-8.95	-9.45	-10.22	-9.61
	3	-9.99	-8.23	-8.45	-8.86	-8.97	-9.10	-10.19	-8.94	-9.27	-10.16	-9.59
	Number of conformations in cluster										
	1	3	3	1	1	3	2	5	12	5	4	8
	2	4	3	1	1	1	1	3	3	1	12	2
	3	2	2	1	1	2	2	5	3	1	4	3
Haloperidole	Lowest binding energy [kcal/mol]										
	1	-11.07	-11.00	-10.36	-10.89	-9.68	-10.26	-11.88	-10.84	-10.31	-10.67	-10.43
	2	-10.63	-10.79	-9.44	-9.45	-9.50	-10.21	-11.20	-10.35	-10.28	-10.50	-10.30
	3	-10.24	-10.01	-9.21	-9.42	-9.30	-10.18	-10.57	-10.34	-10.23	-10.38	-10.12
	Number of conformations in cluster										
	1	3	1	1	1	1	2	1	1	3	2	18
	2	2	1	1	3	5	3	5	5	3	6	1
	3	2	1	3	2	5	3	1	1	1	5	5
Spiperone	Lowest binding energy [kcal/mol]										
	1	-9.29	-9.25	-8.75	-8.88	-9.38	-9.50	-9.50	-8.75	-9.12	-9.38	-9.50
	2	-8.67	-9.00	-7.88	-8.75	-9.00	-8.75	-9.38	-8.62	-9.00	-9.38	-9.25
	3	-8.58	-8.75	-7.88	-8.50	-8.88	-8.62	-9.38	-8.62	-8.75	-9.12	-9.12
	Number of conformations in cluster										
	1	4	2	3	3	1	1	5	2	1	15	2
	2	3	3	1	1	1	2	1	2	3	1	9

	3	2	2	3	1	1	1	7	4	1	14	1
Chlorpromazine						Lowest binding energy [kcal/mol]						
	1	-8.29	-7.88	-8.03	-7.79	-7.89	-8.91	-8.63	-7.61	-8.06	-8.56	-8.74
	2	-8.28	-7.81	-7.69	-7.71	-7.64	-8.33	-8.38	-7.30	-7.60	-8.17	-7.95
	3	-8.27	-7.81	-7.51	-7.63	-7.49	-8.06	-7.98	-7.13	-7.56	-8.16	-7.81
						Number of conformations in cluster						
	1	26	2	1	5	1	1	5	5	7	23	2
	2	10	11	14	30	7	2	18	5	7	9	5
	3	14	1	1	24	2	21	4	3	18	3	10

Table S7 - Docking results for the D$_4$R. The lowest binding energy ΔG_{bind} [kcal/mol] of every run was measured using AutoDock4.2. Calculated conformations were clustered and ranked by energy level. A populated cluster indicates, that the position docked into the receptor is more likely to depict the conformational binding position of the ligand. The three best clusters were chosen and analyzed. NA – Not Available, cases were the number of clusters was lower than 3.

**Receptor state of the MD simulation [ns]**	**Top**	**0**	**55**	**60**	**65**	**70**	**75**	**80**	**85**	**90**	**95**	**100**
Dopamine						Lowest binding energy [kcal/mol]						
	1	-10.04	-9.84	-9.60	-9.99	-10.09	-9.78	-9.41	-8.53	-7.52	-9.87	-9.29
	2	-9.54	-8,68	-8.96	-9.60	-9.45	-8.16	-9.23	-7.81	-7.45	-7.41	-9.11
	3	-9.40	-7.75	-8.68	-8.36	-8.80	-7.71	-9.06	-6.95	-7.16	-6.99	-7.79
						Number of conformations in cluster						
	1	69	62	60	39	62	68	41	39	30	86	13
	2	15	28	19	21	10	6	17	13	30	5	55
	3	8	1	3	8	9	4	7	2	8	2	8
7-OH-DPAT						Lowest binding energy [kcal/mol]						
	1	-9.52	-7.75	-8.28	-8.32	-8.74	-9.25	-8.98	-7.46	-7.56	-9.62	-8.83
	2	-8.44	-7.68	-7.97	-7.82	-8.25	-8.70	-8.45	-7.05	-7.60	-9.41	-8.17
	3	-7.8	-7.52	-7.71	-7.71	-8.17	-8.46	-7.54	-6.53	-7.50	-7.38	-8.04
						Number of conformations in cluster						
	1	30	11	13	5	13	14	6	42	11	15	23

	2	19	20	3	1	10	10	13	9	48	3	1
	3	17	20	1	27	1	7	26	4	16	55	2
Apomorphine		Lowest binding energy [kcal/mol]										
	1	-9.24	-10.33	-9.48	-10.11	-10.19	-9.92	-10.08	-8.41	-9.26	-9.91	-10.10
	2	-9.05	-9.69	-9.09	-9.59	-9.50	-9.83	-9.72	-8.09	-8.98	-9.86	-10.01
	3	-8.94	-9.51	-8.25	-9.27	-9.32	-8.77	-9.56	7-82	-8.72	-7.77	-9.30
		Number of conformations in cluster										
	1	34	44	13	27	28	2	2	12	7	8	10
	2	16	19	39	7	40	51	5	1	21	7	19
	3	9	31	1	42	10	35	13	41	4	51	2
Bromocriptine		Lowest binding energy [kcal/mol]										
	1	+103.73	+34.39	+43.40	+138.76	+154.29	+65.12	+35.85	+87.57	+133.51	+238.63	+57.61
	2	+142.07	+39.22	+73.40	+139.02	+162.10	+127.27	+79.19	+129.02	+156.83	+240.75	+69.87
	3	+198.99	+49.74	+88.72	+169.12	+176.68	+141.46	+95.36	+161.26	+158.24	+250.54	+113.81
		Number of conformations in cluster										
	1	12	38	45	57	4	36	22	26	23	5	58
	2	18	32	15	10	51	1	2	1	18	11	10
	3	3	3	21	8	2	6	9	54	8	8	4
Clozapine		Lowest binding energy [kcal/mol]										
	1	-7.73	-7.05	-7.12	-7.95	-7.64	-6.04	-8.96	-4.70	-5.20	-1.03	-6.09
	2	-7.44	-7.05	-7.06	-7.17	-6.25	-4.27	-6.58	-2.40	-3.52	-0.72	-3.17
	3	-6.75	-6.96	-6.47	-6.76	-6.04	-1.84	-4.58	-0.68	-3.46	+1.59	-2.93
		Number of conformations in cluster										
	1	59	50	8	11	38	49	65	27	15	8	58
	2	2	6	13	22	2	27	13	40	14	44	1
	3	13	17	21	45	31	7	7	3	5	4	4
Nemonapride		Lowest binding energy [kcal/mol]										
	1	-10.46	-9.12	-10.45	-10.09	-10.68	-10.89	-9.92	-9.84	-10.48	-9.59	-10.62
	2	-9.85	-9.07	-9.31	-9.98	-9.50	-10.41	-9.12	-8.77	-10.15	-9.53	-10.26
	3	-9.50	-9.07	-9.08	-9.55	-9.45	-10.29	-8,.93	-7.71	-9.53	-8.34	-9.86
		Number of conformations in cluster										

	1	10	15	1	1	5	20	23	12	25	19	7
	2	3	14	7	1	1	17	15	16	25	16	8
	3	15	12	3	3	4	1	7	6	18	12	1
Sulpiride	Lowest binding energy [kcal/mol]											
	1	-10.62	-10.20	-11.12	-10.65	-12.10	-11.36	-9.20	-8.98	-10.71	-10.53	-12.14
	2	-10.43	-9.83	-10.71	-10.23	-11.06	-11.08	-8.39	-8.63	-10.49	-10.48	-11.29
	3	-9.59	-9.52	-10.25	-10.08	-11.06	-10.91	-8.35	-8.33	-10.28	-9.97	-10.26
	Number of conformations in cluster											
	1	14	4	6	6	1	7	2	2	2	6	1
	2	1	5	5	8	4	4	7	5	2	6	4
	3	2	7	1	1	2	4	10	1	6	5	3
SCH23390	Lowest binding energy [kcal/mol]											
	1	-9.69	-7.15	-9.50	-9.45	-9.71	-10.84	-8.93	-9.93	-8.92	-8.52	-9.60
	2	-9.38	-6.89	-9.37	-9.43	-8.85	-9.47	-8.50	-8.98	-8.75	-8.25	-8.81
	3	-9.22	-6.74	-9.04	-9.02	-8.42	-9.20	-8.50	-8.41	-8.50	-8.23	-8.64
	Number of conformations in cluster											
	1	45	20	11	11	27	28	6	27	71	33	7
	2	17	4	4	6	46	2	9	10	2	22	2
	3	26	5	14	18	8	3	12	5	22	11	18
SKF38393	Lowest binding energy [kcal/mol]											
	1	-10.94	-9.93	-11.02	-10.82	-11.87	-10.18	-10.17	-9.16	-10.16	-9.62	-10.67
	2	-10.19	-9.70	-9.87	-10.72	-9.90	-9.63	-10.10	-8.99	-9.66	-9.01	-10.58
	3	-9.48	-9.60	-9.76	-10.36	-9.17	-9.38	-9.35	-8.60	-8.94	-8.68	-10.28
	Number of conformations in cluster											
	1	14	29	4	42	19	13	5	45	1	31	3
	2	9	14	21	3	17	17	3	16	30	30	12
	3	3	1	7	9	2	3	7	8	3	4	2
Eticlopride	Lowest binding energy [kcal/mol]											
	1	-9.60	-7.73	-8.55	-9.53	-10.50	-9.78	-9.57	-9.10	-9.58	-8.34	-9.02
	2	-9.38	-7.70	-8.48	-9.23	-9.73	-9.53	-7.77	-7.24	-9.48	-8.09	-8.71
	3	-8.64	-7.68	-8.36	-9.20	-9.42	-9.44	-7.49	-6.86	-8.90	-8.08	-8.63

		Number of conformations in cluster										
	1	20	9	11	2	3	32	46	24	6	10	7
	2	8	8	9	11	1	6	5	13	23	2	2
	3	13	1	4	9	7	10	4	6	29	28	4
Risperidone		Lowest binding energy [kcal/mol]										
	1	-8.30	-9.60	-9.16	-7.80	-8.25	-4.64	-6.17	-1.21	-5.83	-4.65	-7.74
	2	-8.27	-9.45	-9.04	-6.79	-8.04	-2.42	-6.13	+0.50	-5.57	-4.63	-7.20
	3	-7.08	-8.23	-8.93	-5.45	-7.86	-2.31	-3.76	+0.51	-5.42	-2.60	-6.97
		Number of conformations in cluster										
	1	12	37	1	28	10	14	9	12	20	14	2
	2	18	1	6	14	14	3	17	5	12	6	6
	3	11	2	6	3	2	1	17	10	2	8	10
Aripiprazole		Lowest binding energy [kcal/mol]										
	1	-8.92	-11.08	-8.05	-11.43	-12.23	-7.46	-4.70	-1.97	-7.60	-8.30	-9.26
	2	-8.86	-9.85	-7.53	-10.35	-9.26	-4.85	-4.05	-1.45	-7.05	-7.83	-8.92
	3	-8.10	-9.44	-7.28	-10.34	-9.08	-4.65	-3.83	-0.97	-6.73	-6.55	-7.75
		Number of conformations in cluster										
	1	2	6	2	6	12	10	7	14	9	6	2
	2	11	1	9	33	2	2	2	4	3	5	20
	3	6	3	2	3	4	3	4	5	2	11	8
Haloperidole		Lowest binding energy [kcal/mol]										
	1	-9.41	-0.40	-10.32	-9.16	-10.42	-6.29	-1.94	+5.37	-0.35	-4.67	-9.18
	2	-8.26	+0.88	-9.66	-8.32	-9.36	-6.08	-1.34	+5.41	+0.12	-1.78	-8.73
	3	NA	+2.57	-9.36	-8.08	-8.98	-5.94	-0.80	+6.89	+0.56	-1.14	-8.55
		Number of conformations in cluster										
	1	33	69	2	58	58	6	30	29	9	35	21
	2	67	13	7	16	13	18	25	1	20	20	7
	3	NA	6	60	15	21	13	2	6	6	2	28
Spiperone		Lowest binding energy [kcal/mol]										

	1	-9.02	-9.64	-10.16	-10.23	-9.16	-9.99	-8.91	-7.42	-10.27	-9.93	-11.67
	2	-8.79	-9.02	-9.36	-9.72	-9.05	-8.42	-8.29	-6.50	-10.17	-9.05	-11.37
	3	-8.45	-9.01	-9.11	-9.40	-8.59	-7.20	-8.05	-6.26	-9,85	-8.49	-11.14
						Number of conformations in cluster						
	1	1	9	24	17	5	4	6	5	19	14	10
	2	16	10	1	16	2	16	12	9	35	3	20
	3	1	15	12	3	26	11	9	6	4	9	18
Chlorpromazine						Lowest binding energy [kcal/mol]						
	1	-8.55	-7.88	-8.24	-8.60	-9.09	-8.91	-8.08	-8.20	-8.44	-8.24	-9.16
	2	-8.42	-7.82	-8.09	-8.40	-9.00	-8.66	-8.00	-7.78	-8.35	-7.80	-8.64
	3	-7.80	-7.75	-8.02	-8.36	-8.84	-8.42	-7.53	-7.56	-8.35	-7.26	-8.30
						Number of conformations in cluster						
	1	5	24	8	11	10	6	3	1	3	30	1
	2	12	14	22	36	12	8	35	25	11	6	5
	3	16	3	22	7	10	7	14	11	5	9	6

Table S8 - Docking results for the D_5R. The lowest binding energy ΔG_{bind} [kcal/mol] of every run was measured using AutoDock4.2. Calculated conformations were clustered and ranked by energy level. A populated cluster indicates, that the position docked into the receptor is more likely to depict the conformational binding position of the ligand. The three best clusters were chosen and analyzed.

**Receptor state of the MD simulation [ns]**	**Top**	**0**	**55**	**60**	**65**	**70**	**75**	**80**	**85**	**90**	**95**	**100**
Dopamine						Lowest binding energy [kcal/mol]						
	1	-10.86	-10.36	-9.72	-9.53	-10.86	-10.45	-10.38	-10.59	-10.55	-10.14	-10.16
	2	-9.67	-9.59	-9.20	-9.29	-10.28	-9.14	-8.68	-9.47	-8.54	-10.09	-9.77
	3	-8.26	-8.02	-8.42	-7.86	-9.66	-7.22	-7.93	-8.27	-8.21	-9.84	-7.83
						Number of conformations in cluster						
	1	29	37	30	32	45	30	45	58	38	23	32
	2	37	12	30	10	14	17	9	10	10	29	24
	3	7	9	9	7	20	15	2	6	8	12	1

7-OH-DPAT		Lowest binding energy [kcal/mol]										
	1	-8.85	-8.33	-9.32	-9.10	-9.34	-9.77	-9.44	-10.09	-9.46	-9.93	-8.79
	2	-8.66	-7.83	-8.68	-8.84	-9.05	-8.80	-7.89	-8.46	-7.88	-8.14	-8.42
	3	-8.50	-7.55	-8.57	-8.35	-8.21	-8.30	-6.98	-8.26	-7.63	-8.12	-6.77
		Number of conformations in cluster										
	1	28	36	27	25	14	18	26	24	38	36	2
	2	4	19	2	18	32	6	15	14	9	9	3
	3	1	4	12	15	8	18	10	1	4	4	1
Apomorphine		Lowest binding energy [kcal/mol]										
	1	-10.92	-10.35	-10.11	-10.99	-10.30	-10.61	-10.39	-11.41	-10.73	-10.41	-10.06
	2	-10.37	-10.31	-9.26	-10.52	-9.12	-9.14	-8.73	-10.27	-10.69	-9.53	-9.01
	3	-9.14	-8.32	-8.78	-10,15	-9.02	-10.48	-8.69	-8.89	-9.07	-8.96	-8.91
		Number of conformations in cluster										
	1	47	31	50	24	65	34	38	45	34	39	49
	2	18	9	17	4	2	8	3	11	19	7	3
	3	14	2	3	7	12	20	24	17	9	40	37
Bromocriptine		Lowest binding energy [kcal/mol]										
	1	-5.09	-9.65	-7.33	-9.33	-7.25	-2.14	-8.74	-9.59	-9.15	-12.23	-5.89
	2	-4.79	-7.87	-7.05	-1.84	+0.25	+2.71	-7.79	-4.62	-5.91	-10.85	-0.24
	3	-3.19	-7.20	-5.01	+2.31	+4.64	+5.97	-6.76	-3.99	+2.43	-10.62	+5.64
		Number of conformations in cluster										
	1	22	16	33	11	9	3	2	15	17	17	24
	2	7	1	25	1	1	23	5	4	6	12	8
	3	3	8	6	3	11	6	20	5	10	1	11
Clozapine		Lowest binding energy [kcal/mol]										
	1	-8.11	-7.80	-8.78	-8.88	-8.52	-8.33	-7.68	-8.73	-8.02	-8.53	-8.24
	2	-7.79	-7.68	-7.93	-8.45	-8.06	-8.07	-7.53	-7.94	-7.46	-8.19	-8.09
	3	-7.58	-7.57	-7.70	-8.31	-7.95	-7.49	-7.36	-7.88	-7.11	-7.47	-7.53
		Number of conformations in cluster										
	1	30	2	45	8	53	62	51	58	45	15	25

	2	15	15	4	16	15	3	8	8	11	15	40
	3	13	12	9	32	17	2	1	2	6	17	5
Nemonapride	Lowest binding energy [kcal/mol]											
	1	-9.84	-10.45	-10.98	-11.11	-10.78	-10.68	-10.86	-10.97	-10.09	-10.58	-4.42
	2	-9.70	-10.41	-10.39	-10.71	-10.74	-10.48	-10.58	-10.06	-9.79	-10.06	-4.08
	3	-9.66	-8.93	-10.28	-10.43	-9.87	-9.92	-9.61	-10.03	-9.70	-9.52	-4.07
	Number of conformations in cluster											
	1	15	5	13	12	11	5	5	17	15	11	1
	2	9	2	6	9	10	5	20	7	14	2	1
	3	2	1	7	4	5	7	6	14	13	8	1
Sulpiride	Lowest binding energy [kcal/mol]											
	1	-10.25	-11.82	-10.13	-11.08	-10.68	-10.42	-19.97	-11.73	-11.40	-12.53	-11.56
	2	-10.10	-9.31	-9.74	-10.99	-10.03	-10.33	-10.53	-11.62	-10.39	-11.56	-10.62
	3	-10.08	-9.10	-9.48	-10.51	-10.01	-10.32	-10.47	-11.47	-9.56	-11.46	-10.11
	Number of conformations in cluster											
	1	5	3	1	2	2	6	4	5	7	2	6
	2	3	2	2	2	5	3	3	4	6	6	4
	3	2	1	1	2	3	3	3	3	1	5	3
SCH23390	Lowest binding energy [kcal/mol]											
	1	-10.05	-9.95	-9.75	-10.53	-10.62	-9.97	-9.32	-9.02	-8.11	-9.88	-10.16
	2	-10.00	-9.44	-9.70	-9.13	-9.25	-9.68	-8.31	-8.94	-8.08	-9.67	-10.00
	3	-9.31	-9.12	-9.53	-8.96	-8.89	-9.53	-8.04	-8.55	-8.01	-9.16	-8.79
	Number of conformations in cluster											
	1	3	32	13	16	9	4	9	2	1	4	3
	2	8	9	6	2	2	5	1	3	4	22	9
	3	2	4	7	10	2	2	3	12	13	5	16
SKF38393	Lowest binding energy [kcal/mol]											
	1	-11.18	-12.56	-11.58	-12.16	-12.88	-11.10	-11.81	-13.61	-10.12	-12.19	–.01
	2	-9.80	-10.78	-10.70	-11.53	-11.25	-10.74	-9.98	-11.02	-9.04	-10.29	-10.55
	3	-9.75	-10.48	-10.54	-11.30	-10.90	-9.00	-9.51	-9.89	-9.03	-10.11	-9.68
	Number of conformations in cluster											

	1	5	30	9	10	6	27	11	14	2	24	6
	2	18	13	12	14	14	13	5	6	3	3	20
	3	9	3	9	14	26	1	2	6	12	1	2
Eticlopride	Lowest binding energy [kcal/mol]											
	1	-8.86	-10.12	-10.59	-10.00	-9.34	-9.39	-8.89	-9.87	-10.29	-9.86	-8.89
	2	-8.80	-8.55	-9.99	-9.78	-9.17	-9.34	-8.62	-8.70	-9.79	-9.82	-8.82
	3	-8.77	-8.10	-9.71	-9.64	-8.89	-9.27	-8.48	-8.53	-9.20	-8.56	-8.51
	Number of conformations in cluster											
	1	6	2	5	5	6	3	2	9	4	5	28
	2	2	10	7	4	3	7	9	2	9	5	2
	3	12	3	18	17	10	2	14	4	10	12	6
Risperidone	Lowest binding energy [kcal/mol]											
	1	-9.98	-9.76	-12.22	-11.40	-9.02	-9.77	-8.85	-10.46	-8.50	-10.74	-10.96
	2	-8.97	-8.63	-11.92	-10.55	-8.94	-9.49	-8.84	-9.72	-8.15	-10.71	-10.70
	3	-8.91	-8.34	-10.94	-10.29	-8.79	-8.52	-8.80	-8.59	-7.54	-10.39	-10.50
	Number of conformations in cluster											
	1	2	5	7	2	4	3	4	5	2	5	17
	2	9	6	11	13	14	1	8	1	30	3	8
	3	2	2	10	17	1	13	3	9	15	6	10
Aripiprazole	Lowest binding energy [kcal/mol]											
	1	-10.87	-10.91	-10.59	-11.52	-12.00	-10.81	-11.04	-10.15	-10.95	-11.08	-10.55
	2	-10.78	-9.84	-9.74	-10.08	-9.97	-9.14	-10.08	-10.07	-9.73	-9.84	-10.39
	3	-10.20	-9.64	-9.67	-9.71	-9.68	-8.93	-9.97	-9.45	-9.44	-9.64	-9.90
	Number of conformations in cluster											
	1	9	2	2	7	2	3	4	3	10	5	1
	2	2	2	6	11	3	2	1	3	3	6	3
	3	10	3	1	5	2	5	8	3	3	6	7
Haloperidole	Lowest binding energy [kcal/mol]											
	1	-9.88	-10.35	-10.36	-10.26	-9.98	-10.38	-9.94	-10.92	-10.28	-10.36	-10.13
	2	-9.54	-9.40	-9.94	-10.23	-9.96	-10.04	-9.67	-10.71	-9.57	-9.92	-10.12
	3	-9.54	-9.31	-9.91	-9.83	-9.80	-9.90	-9.62	-10.32	-9.53	-9.87	-10.05

	Number of conformations in cluster										
	1	8	2	1	9	1	17	2	3	14	3	11
	2	4	4	3	7	5	1	8	9	15	9	16
	3	10	2	2	15	1	5	5	1	3	3	3
Spiperone	Lowest binding energy [kcal/mol]										
	1	-10.57	-10.14	-10.45	-10.17	-10.29	-10.45	-9.39	-10.60	-9.53	-11.51	-10.54
	2	-9.80	-9.87	-10.20	-9.55	-10.21	-10.12	-10.53	-10.38	-9.42	-10.73	-10.51
	3	-9.38	-9.25	-9.88	-9.53	-9.94	-10.11	-10.47	-10.14	-9.42	-10.58	-10.20
	Number of conformations in cluster										
	1	21	1	4	5	2	3	4	1	13	2	1
	2	9	4	2	4	2	20	9	7	2	1	2
	3	5	1	3	4	3	5	9	2	2	9	1
Chlorpromazine	Lowest binding energy [kcal/mol]										
	1	-8.06	-7.69	-8.22	-8.77	-7.98	-8.60	-7.46	-8.16	-7.60	-8.02	-8.11
	2	-7.78	-7.65	-7.96	-8.60	-7.90	-8.51	-7.12	-8.08	-7.28	-7.74	-7.89
	3	-7.56	-7.45	7.94	-7.87	-7.64	-7.99	-7.10	-7.99	-7.28	-7.69	-7.88
	Number of conformations in cluster										
	1	20	25	7	15	8	12	7	13	6	13	17
	2	22	23	8	17	16	23	13	13	2	10	4
	3	3	10	8	3	14	6	14	4	20	15	14

Table S9 - Docking results for the crystal structure templates of D_2R (PDBid: 6CM4), D_3R (PDBid: 3PBL) and D_4R (PDBid: 5WIU) docked with their co-crystalized ligands. The lowest binding energy ΔG_{bind} [kcal/mol] of every run was measured using AutoDock4.2. Calculated conformations were clustered and ranked by energy level. A populated cluster indicates, that the position docked into the receptor is more likely to depict the conformational binding position of the ligand. The three best clusters were chosen and analyzed. As proof of concept the outcome was compared to the corresponding DR-models docked to risperidone, eticlopride and nemonapride at time point 0 ns using the ligand-coordinates used for all dockings.

**Crystal structure template**		**D_2R (6CM4)**		**D_3R (3PBL)**		**D_4R (5WIU)**	
**Co-crystalized ligand**		**Risperidone**		**Eticlopride**		**Nemonapride**	
		Lowest binding energy [kcal/mol]	Number of conformations in cluster	Lowest binding energy [kcal/mol]	Number of conformations in cluster	Lowest binding energy [kcal/mol]	Number of conformations in cluster
Structure from PDB file	1	-13.31	36	-9.31	37	-10.99	5
	2	-13.01	19	-8.97	15	-10.45	1
	3	-12.98	1	-8.94	1	-10.18	32
Corresponding DR models	1	-10.94	9	-9.98	14	-10.46	10
	2	-10.66	2	-9.34	3	-9.85	3
	3	-10.63	11	-9.07	14	-9.50	15

Table S10 - D$_1$R residues with Ballesteros & Weinstein-numbering [4] **participating in different interaction types sorted by ligands.** Data was summarized for all time points. Abbreviations: Hydrocontacts – hydrophobic contacts; SB – salt-bridges; 2.5 Å – 2.5 Å-interactions; HB – hydrogen bonds; cat-π – cat-π-interactions, T-stack – T-stacking-interactions; π-π-stack – π-π-stacking-interactions. Duplicate residues per interaction type are colored red, while unique residues per interaction type are colored green.

Interaction type	Hydrocontacts	SB	2.5 Å	HB	Cat-π	T-stack	π-π-stack
Dopamine	156Asp	3.32Asp	157Gly	74Pro	6.42Gly	6.43Val	
	160Thr	74Pro	160Thr	6.46Cys			
	169Cys		161Ser	3.32Asp			
	172Ser		169Cys	172Ser			
	274Cys		172Leu	172Leu			
	3.29Val		172Ser	169Cys			
	3.32Asp		3.32Asp	157Gly			
	3.33Ile		3.36Ser				
	3.36Ser		5.37Thr				
	5.37Thr		6.46Cys				
	6.30Glu		74Pro				
	6.31Thr						
	6.34Leu						
	6.39Val						
	6.42Gly						
	6.43Val						
	6.46Cys						
	71Gly						
	74Pro						
	75Phe						
7-OH-DPAT	164Glu	3.32Asp	160Thr	156Asp	6.42Gly	6.43Val	
	169Cys		169Cys	169Cys	6.31Thr		
	172Ser		172Leu	172Leu	6.30Glu		
	173Leu		172Ser	172Ser	231Phe		
	2.53Val		3.37Thr	3.37Thr			
	2.57Val		5.37Thr	6.34Leu			
	231Phe		6.34Leu	6.46Cys			
	264Phe		6.46Cys				
	265Cys						
	266Gly						
	271Gln						
	274Cys						
	275Ile						
	3.23Phe						

	3.28Trp						
	3.29Val						
	3.32Asp						
	3.33Ile						
	3.36Ser						
	5.37THR						
	6.30Glu						
	6.31Thr						
	6.34Leu						
	6.39Val						
	6.42Gly						
	6.43Val						
	6.46Cys						
	7.33Asn						
	71Gly						
	74Pro						
	75Phe						
Apomorphine	156Asp		160Thr	169Cys		6.42Gly	274Cys
	169Cys		169Cys	172Leu		6.43Val	
	172Ser		172Ser	6.34Leu		6.58Leu	
	173Leu		173Leu			274Cys	
	2.57Val		6.34Leu				
	274Cys		6.46Cys				
	275Ile						
	3.24Phe						
	3.28Trp						
	3.29Val						
	3.32Asp						
	3.33Ile						
	3.36Ser						
	5.37THR						
	6.34Leu						
	6.39Val						
	6.42Gly						
	6.43Val						
	6.46Cys						
	6.48Trp						
	6.58Leu						
	6.59Pro						
	7.33Asn						
	7.34Thr						

	71Gly						
	74Pro						
	75Phe						
Bromocriptine	1.31Val		1.31Val	157Gly		274Cys	6.39Val
	1.45Ser		1.59Arg	169Cys		6.42Gly	
	1.55Ala		132Leu	172Ser		6.43Val	
	1.59Arg		157Gly	2.50Asp		6.58Leu	
	132Leu		169Cys	3.28Trp		7.34Thr	
	157Gly		172Ser	3.32Asp			
	160thr		2.37val	3.36Ser			
	161ser		2.50asp	3.42Phe			
	168Asn		2.53val	6.39Val			
	169Cys		231Phe	6.46Cys			
	172Ser		266Gly	7.33Asn			
	1Arg		269Glu	7.36Asp			
	2.37val		270Thr				
	2.41phe		274Cys				
	2.45ser		274Ile				
	2.50asp		3.24phe				
	2.53val		3.28Trp				
	2.57val		3.32asp				
	2.58met		3.33Ile				
	231Phe		3.37Thr				
	264Phe		3.39Ser				
	266Gly		3.40Ile				
	267Ser		336Ser				
	269Glu		36His				
	270Thr		37leu				
	274Cys		5.37Thr				
	274Ile		6.29Arg				
	3.24phe		6.31Thr				
	3.29val		6.39Val				
	3.32asp		6.42Gly				
	3.33Ile		6.43Val				
	3.35Cys		6.46Cys				
	3.37Thr		6.59Pro				
	3.39Ser		7.33Asn				
	3.40Ile		7.36Asp				
	336Ser		7.37Val				
	34Phe		75phe				
	36His						

	37leu						
	5.37Ser						
	5.38Tyr						
	6.29Arg						
	6.30Glu						
	6.31Thr						
	6.34Leu						
	6.35Lys						
	6.38Ser						
	6.39Val						
	6.42Gly						
	6.43Val						
	6.46Cys						
	6.58Leu						
	6.59Pro						
	7.33Asn						
	7.34Thr						
	7.36Asp						
	7.37Val						
	74pro						
	75phe						
Clozapine	172Ser		3.28Trp	3.28Trp		274Cys	6.39Val
	2.41Phe		3.32Asp	3.32Asp		6.39Val	274Cys
	2.53Val		3.36Ser	3.36Ser		6.42Gly	
	2.57Val		3.42Phe	3.42Phe		6.43Val	
	231Phe		6.39Val	6.39Val		7.34Thr	
	266Gly		6.46Cys				
	267Ser						
	269Glu						
	271Gln						
	274Cys						
	274Ile						
	3.23Ser						
	3.27Ile						
	3.28Trp						
	3.29Val						
	3.32Asp						
	3.33Ile						
	3.35Cys						
	3.36Ser						
	3.42Phe						

	5.37Thr						
	6.30Glu						
	6.34Leu						
	6.39Val						
	6.42Gly						
	6.43Val						
	6.46Cys						
	6.59Pro						
	7.33Asn						
	7.34Thr						
	7.36Asp						
	7.37Val						
	71Gly						
	74Pro						
	75Phe						
Nemonapride	169Cys	3.32Asp	172Ser	172Leu	6.30Glu	3.28Trp	7.34Thr
	172Leu	74Pro	2.45Ser	172Ser	6.39Val	6.42Gly	227Phe
	172Ser		2.50Asp	2.45Ser	6.42Gly	6.43Val	
	2.41Phe		2.67Ala	2.61Lys	7.34Thr	7.34Thr	
	2.45Ser		266Gly	2.67Ala			
	2.46Leu		3.28Trp	3.28Trp			
	2.49Ser		3.32Asp	3.32Asp			
	2.53Val		3.36Ser	6.46Cys			
	2.57Val		6.39Val	7.33Asn			
	2.61Lys		7.33Asn	74Pro			
	2.67Ala		7.34Thr				
	231Phe		74Pro				
	264Phe						
	265Cys						
	266Gly						
	267Ser						
	269Glu						
	270Thr						
	271Gln						
	274Cys						
	274Ile						
	3.23Ser						
	3.28Trp						
	3.29Val						
	3.32Asp						
	3.33Ile						

	3.35Cys						
	3.36Ser						
	3.39Ser						
	3.40Ile						
	3.42Phe						
	5.37Thr						
	6.30Glu						
	6.35Lys						
	6.39Val						
	6.42Gly						
	6.43Val						
	6.46Cys						
	6.59Pro						
	7.32Ser						
	7.33Asn						
	7.34Thr						
	7.36Asp						
	74Pro						
Sulpiride	1.36Ala	3.32Asp	156Asp	156Asp	6.42Gly	3.28Trp	
	1.43Ile	74Pro	160Thr	169Cys	274Cys	6.39Val	
	1.46Thr		169Cys	172Leu		6.42Gly	
	1.47Leu		172Leu	3.32Asp		7.34Thr	
	1.51Thr		172Ser	3.36Ser			
	2.41Phe		3.28Trp	6.34Leu			
	2.45Ser		3.32Asp	6.46Cys			
	2.53Val		3.36Ser				
	2.57Val		3.42Phe				
	231Phe		5.37Thr				
	264Phe		6.29Arg				
	266Gly		6.34Leu				
	267Ser		6.39Val				
	274Cys		6.46Cys				
	274Ile		7.33Asn				
	3.23Ser						
	3.28Trp						
	3.29Val						
	3.32Asp						
	3.33Ile						
	3.35Cys						
	3.36Ser						
	6.29Arg						

	6.30Glu						
	6.34Leu						
	6.39Val						
	6.42Gly						
	6.43Val						
	6.46Cys						
	6.58Leu						
	6.59Pro						
	7.33Asn						
	7.34Thr						
	71Gly						
	74Pro						
	75Phe						
SCH23390	169Cys	3.32Asp	169Cys	172Ser	6.30Glu	6.39Val	6.39Val
	172Leu		172Leu	3.32Asp	6.39Val	6.42Gly	6.42Gly
	2.41Phe		172Ser	6.39Val	6.42Gly		6.43Val
	2.45Ser		266Gly	6.46Cys			
	2.53Val		3.32Asp	74Pro			
	231Phe		3.36Ser				
	264Phe		5.37Thr				
	265Cys		6.39Val				
	266Gly		6.46Cys				
	274Cys		74Pro				
	274Ile						
	3.23Ser						
	3.29Val						
	3.32Asp						
	3.33Ile						
	3.35Cys						
	3.36Ser						
	3.40Ile						
	3.42Phe						
	6.30Glu						
	6.39Val						
	6.42Gly						
	6.43Val						
	6.46Cys						
	6.58Leu						
	6.59Pro						
	7.32Ser						
	7.33Asn						

	7.34Thr<br>74Pro						
SKF38393	160Thr<br>172Leu<br>172Ser<br>231Phe<br>274Cys<br>3.29Val<br>3.32Asp<br>3.33Ile<br>3.36Ser<br>3.42Phe<br>6.30Glu<br>6.31Thr<br>6.34Leu<br>6.39Val<br>6.42Gly<br>6.43Val<br>6.46Cys<br>6.58Leu<br>7.33Asn<br>71Gly<br>74Pro<br>75Phe	3.32Asp<br>74Pro	157Gly<br>160Thr<br>161Ser<br>169Cys<br>172Leu<br>172Ser<br>3.32Asp<br>5.37Thr<br>6.34Leu<br>6.39Val<br>6.46Cys<br>71Gly	161Ser<br>169Cys<br>172Leu<br>172Ser<br>3.32Asp<br>6.34Leu<br>6.46Cys		6.43Val	274Cys<br>6.30Glu<br>6.39Val<br>6.42Gly<br>6.43Val
Eticlopride	169Cys<br>172Leu<br>172Ser<br>2.41Phe<br>2.45Ser<br>2.46Leu<br>2.53Val<br>2.57Val<br>2.67Ala<br>231Phe<br>264Phe<br>266Gly<br>267Ser<br>271Gln<br>274Cys<br>274Ile<br>3.28Trp	3.32Asp<br>74Pro	267Ser<br>3.28Trp<br>3.32Asp<br>3.36Ser<br>6.39Val<br>6.46Cys<br>7.33Asn<br>74Pro	3.32Asp<br>3.36Ser<br>6.39Val<br>6.46Cys<br>7.33Asn<br>74Pro	6.42Gly	274Cys<br>6.42Gly	6.39Val<br>6.42Gly

	3.29Val						
	3.32Asp						
	3.33Ile						
	3.35Cys						
	3.36Ser						
	3.42Phe						
	5.37Thr						
	6.30Glu						
	6.39Val						
	6.42Gly						
	6.43Val						
	6.46Cys						
	6.48Trp						
	6.59Pro						
	7.32Ser						
	7.33Asn						
	7.34Thr						
	74Pro						
Risperidone	160Thr		266Gly	266Gly		6.58Leu	264Phe
	168Asn		274Cys	3.28Trp		6.43Val	6.30Glu
	169Cys		3.28Trp	3.32Asp		6.42Gly	
	172Leu		3.32Asp	6.46Cys		274Cys	
	172Ser		5.37Thr	7.36Asp		231Phe	
	2.45Ser		6.34Leu				
	2.49Ser		6.39Val				
	2.50Asp		6.46Cys				
	2.53Val		6.58Leu				
	2.57Val		7.36Asp				
	2.58Met						
	231Phe						
	264Phe						
	265Cys						
	266Gly						
	267Ser						
	274Cys						
	274Ile						
	3.28Trp						
	3.29Val						
	3.32Asp						
	3.33Ile						
	3.35Cys						

	3.36Ser<br>3.39Ser<br>3.40Ile<br>3.42Phe<br>5.37Thr<br>6.30Glu<br>6.31Thr<br>6.34Leu<br>6.35Lys<br>6.39Val<br>6.42Gly<br>6.43Val<br>6.46Cys<br>6.58Leu<br>6.59Pro<br>7.32Ser<br>7.33Asn<br>7.34Thr<br>7.36Asp<br>74Pro<br>75Phe						
Aripiprazole	157Gly<br>168Asn<br>169Cys<br>172Ser<br>2.53Val<br>2.57Val<br>264PHE<br>266Gly<br>274Cys<br>275Ile<br>3.24Phe<br>3.28Trp<br>3.29Val<br>3.32Asp<br>3.33Ile<br>3.35Cys<br>3.36Ser<br>3.39Ser<br>3.40Ile<br>5.37THR		157Gly<br>160Thr<br>161Ser<br>169Cys<br>172Leu<br>172Ser<br>2.50Asp<br>3.28Trp<br>3.36Ser<br>3.39Ser<br>5.37Thr<br>6.34Leu<br>6.46Cys<br>7.33Asn<br>7.34Thr<br>7.36Asp	157Gly<br>169Cys<br>172Leu<br>3.39Ser<br>6.46Cys<br>7.36Asp		274Cys<br>6.42GLY<br>6.43VAL<br>6.58Leu<br>7.34Thr	7.34Thr

	6.30Glu						
	6.34Leu						
	6.39Val						
	6.42Gly						
	6.43Val						
	6.46Cys						
	6.58LEU						
	6.59PRO						
	7.33Asn						
	7.34Thr						
	7.36Asp						
	71Gly						
	74Pro						
	75Phe						
Haloperidole	172Leu	3.32Asp	3.28Trp	3.28Trp	274Cys	6.42Gly	231Phe
	172Ser	74Pro	3.32Asp	3.32Asp	6.42Gly	6.43Val	6.31Thr
	2.45Ser		6.39Val	7.33Asn			6.43Val
	2.53Val		6.46Cys	74Pro			
	2.57Val		7.33Asn				
	2.61Lys		74Pro				
	231Phe						
	264Phe						
	265Cys						
	266Gly						
	267Ser						
	271Gln						
	274Cys						
	274Ile						
	3.28Trp						
	3.29Val						
	3.32Asp						
	3.33Ile						
	3.35Cys						
	3.36Ser						
	3.42Phe						
	5.37Thr						
	6.30Glu						
	6.39Val						
	6.42Gly						
	6.43Val						
	6.46Cys						

	6.58Leu<br>6.59Pro<br>7.33Asn<br>7.34Thr<br>7.36Asp<br>71Gly<br>74Pro							
Spiperone	168Asn<br>169Cys<br>172Ser<br>2.45Ser<br>2.53Val<br>2.57Val<br>2.61Lys<br>2.67Ala<br>231Phe<br>264Phe<br>266Gly<br>267Ser<br>271Gln<br>274Cys<br>274Ile<br>3.28Trp<br>3.29Val<br>3.32Asp<br>3.33Ile<br>3.35Cys<br>3.36Ser<br>3.40Ile<br>5.37Thr<br>6.30Glu<br>6.31Thr<br>6.34Leu<br>6.35Lys<br>6.39Val<br>6.42Gly<br>6.43Val<br>6.46Cys<br>6.48Trp<br>6.55Asn<br>6.58Leu	3.32Asp<br>74Pro	172Leu<br>172Ser<br>2.61Lys<br>2.67Ala<br>274Cys<br>274Ile<br>3.28Trp<br>3.37Thr<br>5.37Thr<br>6.34Leu<br>6.39Val<br>6.46Cys<br>7.33Asn<br>7.34Thr	2.61Lys<br>3.28Trp<br>6.46Cys		274Cys<br>6.42Gly<br>6.43Val	3.28Trp<br>6.39Val<br>6.42Gly<br>7.34Thr

	6.59Pro						
	7.32Ser						
	7.33Asn						
	7.34Thr						
	7.36Asp						
	71Gly						
	74Pro						
	75Phe						
Chlorpromazine	2.41Phe		6.39Val	3.28Trp		274Cys	264Phe
	2.45Ser		7.33Asn			6.30Glu	274Cys
	2.53Val					6.39Val	3.28Trp
	2.57Val					6.42Gly	6.39Val
	231Phe						6.42Gly
	264Phe						
	266Gly						
	267Ser						
	271Gln						
	274Cys						
	274Ile						
	3.28Trp						
	3.29Val						
	3.32Asp						
	3.33Ile						
	3.35Cys						
	3.36Ser						
	3.42Phe						
	5.37Thr						
	6.30Glu						
	6.31Thr						
	6.34Leu						
	6.39Val						
	6.42Gly						
	6.43Val						
	6.46Cys						
	6.58Leu						
	6.59Pro						
	7.32Ser						
	7.33Asn						
	7.34Thr						
	74Pro						
	75Phe						

Table S11 - D2R residues with Ballesteros & Weinstein-numbering [4] **participating in different interaction types sorted by ligands.** Data was summarized for all time points. Abbreviations: Hydrocontacts – hydrophobic contacts; SB – salt-bridges; 2.5 Å – 2.5 Å-interactions; HB – hydrogen bonds; cat-π – cat-π-interactions, T-stack – T-stacking-interactions; π-π-stack – π-π-stacking-interactions. Duplicate residues per interaction type are colored red, while unique residues per interaction type are colored green.

Interaction type	Hydrocontacts	SB	2.5 Å	HB	Cat-π	T-stack	π-π-stack
Dopamine	156Ile	3.32Asp	3.32Asp	3.32Asp	6.55His	6.51Phe	6.48Trp
	3.32Asp		5.39Val	5.39Val			6.55His
	3.33Val		5.42Ser	5.42Ser			
	3.36Cys		5.43Ser	5.43Ser			
	5.38Phe		5.46Ser	5.46Ser			
	5.39Val		6.48Trp	7.43Tyr			
	5.42Ser		6.55His				
	5.43Ser		7.35Tyr				
	5.46Ser		7.43Tyr				
	5.47Phe						
	6.48Trp						
	6.51Phe						
	6.52Phe						
	6.55His						
	7.39Thr						
	7.43Tyr						
7-OH-DPAT	156Ile	3.32Asp	5.39Val	5.39Val	6.48Trp	6.51Phe	5.47Phe
	3.28Phe		5.42Ser	5.43Ser	6.55His	6.55His	6.48Trp
	3.29Val		5.43Ser	6.55His			6.52Phe
	3.32Asp		6.45Ile				6.55His
	3.33Val		6.55His				
	3.36Cys						
	3.37Thr						
	5.38Phe						
	5.39Val						
	5.42Ser						
	5.43Ser						
	5.45Val						
	5.46Ser						
	5.47Phe						
	5.48Tyr						
	6.44Phe						
	6.45Ile						
	6.48Trp						

	6.51Phe						
	6.52Phe						
	6.55His						
	7.35Tyr						
	7.39Thr						
	7.42Gly						
	7.43Tyr						
	72Trp						
Apomorphine	156Ile		3.32Asp	3.32Asp	6.55His	6.48Trp	5.38Phe
	3.32Asp		3.36Cys	5.39Val		6.51Phe	6.48Trp
	3.33Val		5.39Val	5.42Ser		7.35Tyr	6.51Phe
	3.36Cys		5.42Ser	5.46Ser			6.55His
	5.38Phe		5.43Ser	6.55His			
	5.39Val		5.46Ser				
	5.42Ser		6.48Trp				
	5.43Ser		6.55His				
	5.46Ser						
	5.47Phe						
	6.48Trp						
	6.51Phe						
	6.52Phe						
	6.55His						
	7.39Thr						
	7.42Gly						
	7.43Tyr						
	72Trp						
Bromocriptine	156Ile		156Ile	3.32Asp	6.55His		6.48Trp
	2.53Val		2.53Val	3.36Cys			6.51Phe
	2.57Val		3.29Val	5.38Phe			6.55His
	3.28Phe		3.32Asp	5.42Ser			
	3.29Val		3.36Cys	5.46Ser			
	3.32Asp		3.40Ile	5.48Tyr			
	3.36Cys		5.38Phe	6.45Ile			
	3.39Ser		5.39Val	6.48Trp			
	3.40Ile		5.43Ser	7.35Tyr			
	5.38Phe		5.46Ser	7.43Tyr			
	5.39Val		5.47Phe				
	5.42Ser		5.48Tyr				
	5.43Ser		6.44Phe				
	5.46Ser		6.48Trp				
	5.47Phe		6.52Phe				

	5.48Tyr		6.55His				
	6.44Phe		6.58Asn				
	6.45Ile		7.35Tyr				
	6.48Trp		7.43Tyr				
	6.49Leu						
	6.51Phe						
	6.52Phe						
	6.55His						
	6.58Asn						
	7.35Tyr						
	7.39Thr						
	7.43Tyr						
	72Trp						
Clozapine	156Ile		3.32Asp	3.32Asp	6.55His	5.38Phe	3.28Phe
	2.53Val		3.36Cys	5.46Ser		6.48Trp	6.48Trp
	2.57Val		5.42Ser	6.55His		6.51Phe	6.55His
	3.28Phe		5.46Ser	6.58Asn		6.55His	
	3.29Val		6.55His			7.35Tyr	
	3.32Asp		6.58Asn			7.43Tyr	
	3.33Val						
	3.36Cys						
	3.39Ser						
	5.38Phe						
	5.39Val						
	5.42Ser						
	5.43Ser						
	5.45Val						
	5.46Ser						
	5.47Phe						
	6.44Phe						
	6.48Trp						
	6.51Phe						
	6.52Phe						
	6.54Thr						
	6.55His						
	6.58Asn						
	7.35Tyr						
	7.39Thr						
	7.42Gly						
	7.43Tyr						
	7.45Asn						

	72Trp						
Nemonapride	1.44Ala	1.53Val	1.44Ala	3.32Asp	3.28Phe	3.28Phe	3.28Phe
	1.45Val	3.32Asp	3.32Asp	3.36Cys	6.55His	6.48Trp	5.38Phe
	1.48Phe		5.43Ser	5.43Ser		6.52Phe	
	1.49Gly		5.46Ser	5.46Ser		72Trp	
	1.50Asn		6.48Trp	6.48Trp			
	1.53Val		6.52Phe	7.43Tyr			
	1.54Cys		6.55His				
	155Ile		7.35Tyr				
	156Ile		7.43Tyr				
	2.57Val						
	2.61Val						
	2.64Leu						
	3.28Phe						
	3.29Val						
	3.32Asp						
	3.33Val						
	3.36Cys						
Sulpiride	3.43Leu						
	3.48Ile						
	3.52Thr						
	3Arg						
	4Pro						
	5.38Phe						
	5.39Val						
	5.42Ser						
	5.43Ser						
	5.46Ser						
	5.47Phe						
	6.48Trp						
	6.51Phe						
	6.52Phe						
	6.55His						
	7.35Tyr						
	7.36Ser						
	7.39Thr						
	7.42Gly						
	7.43Tyr						
	72Trp						
SCH23390	156Ile	3.32Asp	3.32Asp	3.32Asp	6.55His	3.28Phe	6.51Phe
	2.53Val		5.39Val	5.39Val	only at	5.38Phe	6.55His

					timetpoint 70 ns		
	2.61Val<br>2.64Leu<br>3.28Phe<br>3.29Val<br>3.32Asp<br>3.33Val<br>3.36Cys<br>5.38Phe<br>5.39Val<br>5.42Ser<br>5.46Ser<br>6.48Trp<br>6.51Phe<br>6.52Phe<br>6.55His<br>7.35Tyr<br>7.36Ser<br>7.39Thr<br>7.40Trp<br>7.42Gly<br>7.43Tyr<br>72Trp		5.42Ser<br>5.43Ser<br>5.46Ser<br>6.48Trp<br>6.51Phe<br>6.55His<br>7.35Tyr<br>7.39Thr<br>7.40Trp<br>7.43Tyr	5.42Ser<br>5.43Ser<br>5.46Ser<br>6.48Trp<br>7.35Tyr<br>7.43Tyr		6.48Trp<br>6.51Phe	
SKF38393	156Ile<br>3.29Val<br>3.32Asp<br>3.33Val<br>3.36Cys<br>3.39Ser<br>5.38Phe<br>5.39Val<br>5.42Ser<br>5.43Ser<br>5.46Ser<br>5.47Phe<br>6.44Phe<br>6.48Trp<br>6.51Phe<br>6.52Phe<br>6.55His	3.32Asp	5.39Val<br>5.42Ser<br>5.43Ser<br>5.46Ser<br>6.55His<br>7.35Tyr<br>7.43Tyr	5.39Val<br>5.42Ser<br>5.43Ser<br>6.55His	6.48Trp<br>6.51Phe<br>6.55His	5.38Phe<br>6.48Trp<br>6.51Phe<br>6.55His	5.47Phe<br>6.48Trp<br>6.51Phe<br>6.55His

	7.35Tyr<br>7.39Thr<br>7.42Gly<br>7.43Tyr						
Eticlopride	156Ile<br>3.32Asp<br>3.33Val<br>3.36Cys<br>5.38Phe<br>5.42Ser<br>5.43Ser<br>5.46Ser<br>6.48Trp<br>6.51Phe<br>6.52Phe<br>6.55His<br>7.35Tyr<br>7.39Thr<br>7.42Gly<br>7.43Tyr	3.32Asp	3.32Asp<br>5.39Val<br>5.42Ser<br>5.43Ser<br>5.46Ser<br>6.48Trp<br>6.55His<br>7.43Tyr	3.32Asp<br>3.33Val<br>5.39Val<br>5.42Ser<br>5.46Ser<br>6.55His<br>7.43Tyr	6.55His	6.48Trp<br>6.51Phe	6.48Trp<br>6.51Phe<br>6.52Phe<br>6.55His
Risperidone	156Ile<br>2.57Val<br>2.61Val<br>2.64Leu<br>3.28Phe<br>3.29Val<br>3.32Asp<br>3.33Val<br>3.36Cys<br>5.38Phe<br>5.39Val<br>5.42Ser<br>5.43Ser<br>5.46Ser<br>5.47Phe<br>6.44Phe<br>6.48Trp<br>6.51Phe<br>6.52Phe<br>6.55His<br>7.35Tyr		3.32Asp<br>3.36Cys<br>5.42Ser<br>5.46Ser<br>6.48Trp<br>7.35Tyr<br>7.40Trp<br>7.43Tyr	3.32Asp<br>5.46Ser<br>6.48Trp<br>7.35Tyr<br>7.43Tyr	6.55His	5.38Phe<br>6.48Trp<br>6.51Phe<br>6.55His<br>72Trp	6.48Trp<br>6.52Phe

	7.36Ser<br>7.39Thr<br>7.40Trp<br>7.42Gly<br>7.43Tyr<br>72Trp						
Aripiprazole	156Ile<br>2.57Val<br>2.61Val<br>2.64Leu<br>3.28Phe<br>3.29Val<br>3.32Asp<br>3.33Val<br>3.36Cys<br>5.38Phe<br>5.39Val<br>5.42Ser<br>5.43Ser<br>5.46Ser<br>5.47Phe<br>6.44Phe<br>6.48Trp<br>6.51Phe<br>6.52Phe<br>6.54Thr<br>6.55His<br>7.35Tyr<br>7.36Ser<br>7.39Thr<br>7.40Trp<br>7.42Gly<br>7.43Tyr<br>7.45Asn<br>72Trp		3.32Asp<br>5.38Phe<br>5.39Val<br>5.42Ser<br>5.43Ser<br>5.46Ser<br>6.48Trp<br>6.51Phe<br>6.55His<br>7.35Tyr<br>7.43Tyr	5.39Val<br>5.42Ser<br>5.43Ser<br>5.46Ser<br>6.51Phe<br>7.35Tyr	6.55His	3.28Phe<br>5.38Phe<br>6.48Trp<br>6.51Phe<br>6.55His<br>72Trp	6.48Trp<br>6.51Phe<br>6.55His<br>7.43Tyr
Haloperidole	156Ile<br>2.53Val<br>2.57Val<br>3.28Phe<br>3.29Val<br>3.32Asp	3.32Asp	3.32Asp<br>3.36Cys<br>3.40Ile<br>5.42Ser<br>5.46Ser<br>6.48Trp	3.32Asp<br>5.42Ser<br>5.46Ser<br>6.48Trp	6.48Trp<br>6.55His	6.52Phe<br>6.55His<br>7.43Tyr	3.28Phe<br>5.47Phe<br>6.48Trp<br>6.52Phe<br>6.55His

	3.33Val		7.43Tyr				
	3.36Cys						
	3.39Ser						
	3.40Ile						
	5.38Phe						
	5.39Val						
	5.42Ser						
	5.43Ser						
	5.46Ser						
	5.47Phe						
	6.44Phe						
	6.48Trp						
	6.51Phe						
	6.52Phe						
	6.55His						
	7.35Tyr						
	7.39Thr						
	7.42Gly						
	7.43Tyr						
	72Trp						
Spiperone	156Ile	3.32Asp	156Ile	6.48Trp	6.48Trp	3.28Phe	6.48Trp
	2.53Val		3.32Asp	6.55His	6.55His	6.48Trp	6.51Phe
	3.28Phe		3.33Val	7.35Tyr		6.51Phe	6.55His
	3.29Val		3.36Cys	7.43Tyr		6.55His	
	3.32Asp		6.48Trp			7.43Tyr	
	3.33Val		6.55His				
	3.35Met		7.35Tyr				
	3.36Cys		7.39Thr				
	3.39Ser		7.42Gly				
	3.40Ile		7.43Tyr				
	5.38Phe		7.46Ser				
	5.39Val						
	5.42Ser						
	5.43Ser						
	5.46Ser						
	5.47Phe						
	6.44Phe						
	6.48Trp						
	6.51Phe						
	6.52Phe						
	6.55His						

	7.35Tyr<br>7.38Phe<br>7.39Thr<br>7.42Gly<br>7.43Tyr<br>72Trp						
Chlorpromazine	156Ile<br>2.53Val<br>2.57Val<br>3.29Val<br>3.32Asp<br>3.33Val<br>3.36Cys<br>5.38Phe<br>5.39Val<br>5.42Ser<br>5.43Ser<br>5.46Ser<br>5.47Phe<br>6.44Phe<br>6.48Trp<br>6.51Phe<br>6.52Phe<br>6.55His<br>7.39Thr<br>7.42Gly<br>7.43Tyr		6.55His<br>only at timepoint 85		6.55His	5.38Phe<br>6.48Trp<br>6.51Phe	6.48Trp<br>6.51Phe<br>6.55His

Table S12 - D_3R residues with Ballesteros & Weinstein-numbering [4] **participating in different interaction types sorted by ligands.** Data was summarized for all time points. Abbreviations: Hydrocontacts – hydrophobic contacts; SB – salt-bridges; 2.5 Å – 2.5 Å-interactions; HB – hydrogen bonds; cat-π – cat-π-interactions, T-stack – T-stacking-interactions; π-π-stack – π-π-stacking-interactions. Duplicate residues per interaction type are colored red, while unique residues per interaction type are colored green.

Interaction type	Hydrocontacts	SB	2.5 Å	HB	Cat-π	T-stack	π-π-stack
Dopamine	159Ile	3.32Asp	3.32Asp	3.32Asp	2.48Val	6.51Phe	2.48Val
	2.42Leu		3.33Val	5.39Val	6.55His	6.52Phe	35Ala
	2.44Val		5.38Phe	5.42Ser		6.55His	38Thr
	2.45Ser		5.39Val	5.43Ser			6.52Phe
	2.46Leu		5.42Ser	6.51Phe			
	2.47Ala		5.43Ser				
	2.48Val		5.46Ser				
	3.32Asp		6.52Phe				
	3.33Val		74Phe				
	3.36Cys						
	34Arg						
	35Ala						
	38Thr						
	5.38Phe						
	5.39Val						
	5.42Ser						
	6.48Trp						
	6.51Phe						
	6.52Phe						
	6.55His						
7-OH-DPAT	1.58Leu	3.32Asp	1.58Leu	5.39Val	2.14Tyr	2.46Leu	2.48Val
	159Ile		3.36Cys	5.43Ser	2.42Leu	2.47Ala	2.49Ala
	2.41Tyr		5.39Val		2.48Val	2.50Asp	35Ala
	2.42Leu		5.42Ser		2.49Ala	5.38Phe	6.52Phe
	2.43Val		5.43Ser		34Arg	6.52Phe	6.55His
	2.44Val		5.46Ser		6.48Trp	6.55His	
	2.45Ser				6.55His		
	2.46Leu						
	2.47Ala						
	2.48Val						
	2.49Ala						
	2.50Asp						
	3.29Val						
	3.32Asp						

	3.33Val						
	3.36Cys						
	34Arg						
	35Ala						
	38Thr						
	5.38Phe						
	5.39Val						
	5.42Ser						
	5.46Ser						
	6.48Trp						
	6.51Phe						
	6.52Phe						
	6.55His						
	69Gly						
	7.39Thr						
	7.42Gly						
	7.43Tyr						
	70Gly						
	73Asn						
Apomorphine	159Ile		2.14Tyr	3.32Asp	2.48Val	2.41Tyr	2.44Val
	2.41Tyr		2.47Ala	5.39Val	38Thr	2.42Leu	2.48Val
	2.42Leu		3.32Asp	5.42Ser	6.55His	2.43Val	34Arg
	2.44Val		3.36Cys	5.43Ser		2.45Ser	38Thr
	2.45Ser		5.39Val	5.46Ser		2.46Leu	6.48Trp
	2.46Leu		5.42Ser			38Thr	6.52Phe
	2.47Ala		5.43Ser			6.48Trp	6.55His
	2.48Val		5.46Ser			6.51Phe	
	2.50Asp		6.52Phe			6.52Phe	
	2.57Val		6.55His			6.55His	
	3.29Val						
	3.32Asp						
	3.33Val						
	3.35Met						
	3.36Cys						
	34Arg						
	35Ala						
	38Thr						
	39Thr						
	5.38Phe						
	5.39Val						
	5.42Ser						

	5.46Ser						
	6.44Phe						
	6.47Cys						
	6.48Trp						
	6.51Phe						
	6.52Phe						
	6.55His						
	7.35Tyr						
	7.38Thr						
	7.39Thr						
	7.42Gly						
	7.43Tyr						
	70Gly						
	74Phe						
	1.54Cys		1.58Leu	159Ile	38Thr	2.42Leu	2.42Leu
	1.57Val		157Cys	2.48Val	6.55His	2.43Val	2.43Val
	1.58Leu		159Ile	2.60Trp		2.45Ser	2.45Ser
	157Cys		2.39Thr	3.29Val		2.46Leu	2.46Leu
	158Ser		2.41Tyr	3.32Asp		34Arg	34Arg
	159Ile		2.43Val	3.35Met		38Thr	38Thr
	2.39Thr		2.44Val	3.36Cys		6.48Trp	6.48Trp
	2.41Tyr		2.48Val	5.39Val		6.51Phe	6.51Phe
	2.42Leu		2.60Trp	5.46Ser			
	2.43Val		2.61Val	6.55His			
	2.44Val		2.64Leu	7.35Tyr			
	2.45Ser		3.29Val	7.38Thr			
	2.46Leu		3.32Asp				
Bromocriptine	2.48Val		3.33Val				
	2.53Val		3.36Cys				
	2.57Val		3.40Ile				
	2.60Trp		34Arg				
	2.61Val		38Thr				
	2.64Leu		39Thr				
	2.65Glu		5.38Phe				
	2.66Val		5.39Val				
	3.28Phe		5.42Ser				
	3.29Val		5.46Ser				
	3.32Asp		5.47Phe				
	3.33Val		6.44Phe				
	3.35Met		6.48Trp				
	3.36Cys		6.51Phe				

	3.40Ile		6.52Phe				
	34Arg		6.55His				
	35Ala		69Gly				
	38Thr		7.35Tyr				
	39Thr		7.38Thr				
	5.38Phe		7.39Thr				
	5.39Val		7.42Gly				
	5.42Ser		7.43Tyr				
	5.43Ser		74Phe				
	5.46Ser						
	5.47Phe						
	6.44Phe						
	6.47Cys						
	6.48Trp						
	6.51Phe						
	6.52Phe						
	6.55His						
	69Gly						
	7.35Tyr						
	7.38Thr						
	7.39Thr						
	7.42Gly						
	7.43Tyr						
	70Gly						
	71Val						
	73Asn						
	74Phe						
	75Ser						
Clozapine	1.58Leu		2.46Leu	3.32Asp	2.48Val	2.45Ser	1.58Leu
	157Cys		3.32Asp	38Thr	38Thr	2.46Leu	2.43Val
	158Ser		3.36Cys	6.48Trp	6.55His	34Arg	2.49Ala
	159Ile		35Ala	6.55His		6.51Phe	3.28Phe
	2.41Tyr		38Thr				6.48Trp
	2.42Leu		6.48Trp				
	2.43Val		6.52Phe				
	2.44Val		6.55His				
	2.45Ser		71Val				
	2.46Leu						
	2.48Val						
	2.49Ala						
	2.53Val						

	2.57Val<br>3.28Phe<br>3.29Val<br>3.32Asp<br>3.33Val<br>3.36Cys<br>34Arg<br>35Ala<br>38Thr<br>39Thr<br>5.38Phe<br>5.39Val<br>5.42Ser<br>5.43Ser<br>6.48Trp<br>6.51Phe<br>6.52Phe<br>6.55His<br>6.56Val<br>7.39Thr<br>7.42Gly<br>7.43Tyr<br>71Val<br>74Phe<br>75Ser						
Nemonapride	1.58Leu<br>159Ile<br>2.41Tyr<br>2.42Leu<br>2.43Val<br>2.44Val<br>2.45Ser<br>2.46Leu<br>2.47Ala<br>2.48Val<br>2.50Asp<br>2.53Val<br>2.57Val<br>2.61Val<br>2.64Leu<br>3.28Phe	3.32Asp	2.53Val<br>2.61Val<br>3.32Asp<br>3.36Cys<br>5.42Ser<br>5.46Ser<br>6.48Trp<br>6.55His<br>7.42Gly<br>7.43Tyr<br>75Ser	2.61Val<br>3.32Asp<br>3.36Cys<br>5.42Ser<br>6.48Trp<br>7.43Tyr<br>75Ser	2.42Leu<br>38Thr<br>6.55His	2.41Tyr<br>2.43Val<br>6.48Trp<br>6.51Phe<br>7.43Tyr<br>74Phe	1.58Leu<br>2.42Leu<br>2.50Asp<br>6.48Trp

	3.29Val						
	3.32Asp						
	3.33Val						
	3.35Met						
	3.36Cys						
	3.39Ser						
	34Arg						
	35Ala						
	38Thr						
	5.38Phe						
	5.39Val						
	5.42Ser						
	5.46Ser						
	6.44Phe						
	6.48Trp						
	6.51Phe						
	6.52Phe						
	6.55His						
	7.38Thr						
	7.39Thr						
	7.42Gly						
	7.43Tyr						
	70Gly						
	73Asn						
	75Ser						
Sulpiride	1.58Leu	3.32Asp	1.58Leu	2.45Ser	1.39Tyr	2.45Ser	2.43Val
	158Ser		2.45Ser	2.49Ala	2.48Val	2.50Asp	2.44Val
	159Ile		2.48Val	3.32Asp	38Thr	3.28Phe	2.48Val
	2.41Tyr		2.49Ala	38Thr	5.38Phe	38Thr	38Thr
	2.42Leu		3.32Asp	5.39Val	6.48Trp	6.48Trp	5.38Phe
	2.44Val		3.36Cys	5.42Ser	6.55His	6.51Phe	6.51Phe
	2.45Ser		38Thr	5.46Ser		6.55His	6.52Phe
	2.46Leu		5.39Val	6.48Trp			
	2.47Ala		5.42Ser	7.43Tyr			
	2.48Val		5.43Ser	74Phe			
	2.49Ala		5.46Ser				
	2.50Asp		6.48Trp				
	2.53Val		6.55His				
	2.57Val		7.39Thr				
	2.61Val		7.43Tyr				
	3.28Phe						

	3.29Val						
	3.32Asp						
	3.33Val						
	3.35Met						
	3.36Cys						
	34Arg						
	35Ala						
	38Thr						
	5.38Phe						
	5.39Val						
	5.42Ser						
	6.44Phe						
	6.48Trp						
	6.51Phe						
	6.52Phe						
	6.55His						
	69Gly						
	7.35Tyr						
	7.36Ser						
	7.39Thr						
	7.43Tyr						
	70Gly						
	73Asn						
	74Phe						
SCH23390	1.58Leu	3.32Asp	1.58Leu	2.48Val	2.48Val	2.44Val	2.42Leu
	159Ile		2.42Leu	5.38Phe	34Arg	34Arg	2.45Ser
	2.42Leu		2.48Val	5.43Ser	6.55His	5.38Phe	2.47Ala
	2.43Val		3.36Cys			6.51Phe	2.48Val
	2.44Val		5.39Val			6.55His	34Arg
	2.45Ser		5.42Ser				35Ala
	2.46Leu		5.43Ser				38Thr
	2.47Ala		6.48Trp				6.48Trp
	2.48Val		7.39Thr				6.51Phe
	2.53Val		7.43Tyr				6.52Phe
	2.57Val						6.55His
	3.29Val						
	3.32Asp						
	3.33Val						
	3.35Met						
	3.36Cys						
	34Arg						

	35Ala<br>38Thr<br>5.38Phe<br>5.39Val<br>5.42Ser<br>6.48Trp<br>6.51Phe<br>6.52Phe<br>6.55His<br>7.35Tyr<br>7.39Thr<br>7.43Tyr<br>70Gly<br>74Phe						
SKF38393	1.58Leu<br>159Ile<br>2.41Tyr<br>2.42Leu<br>2.43Val<br>2.44Val<br>2.45Ser<br>2.46Leu<br>2.47Ala<br>2.48Val<br>2.49Ala<br>2.50Asp<br>2.57Val<br>3.32Asp<br>3.33Val<br>3.36Cys<br>34Arg<br>35Ala<br>5.38Phe<br>5.39Val<br>5.42Ser<br>5.46Ser<br>5.47Phe<br>6.48Trp<br>6.51Phe<br>6.52Phe<br>6.55His	3.32Asp	159Ile<br>2.49Ala<br>2.50Asp<br>3.32Asp<br>5.39Val<br>5.42Ser<br>5.43Ser<br>5.46Ser<br>6.51Phe<br>6.55His	2.49Ala<br>3.32Asp<br>3.33Val<br>5.39Val<br>5.42Ser<br>5.43Ser<br>6.51Phe	2.48Val<br>6.51Phe<br>6.55His	2.44Val<br>35Ala<br>38Thr<br>5.38Phe<br>6.48Trp<br>6.51Phe<br>6.52Phe<br>6.55His	1.58Leu<br>2.45Ser<br>2.48Val<br>6.52Phe

	70Gly						
Eticlopride	1.54Cys<br>1.58Leu<br>157Cys<br>158Ser<br>159Ile<br>2.41Tyr<br>2.42Leu<br>2.43Val<br>2.44Val<br>2.45Ser<br>2.46Leu<br>2.47Ala<br>2.48Val<br>2.50Asp<br>2.53Val<br>2.57Val<br>2.61Val<br>2.65Glu<br>3.28Phe<br>3.29Val<br>3.32Asp<br>3.33Val<br>3.35Met<br>3.36Cys<br>34Arg<br>35Ala<br>38Thr<br>5.39Val<br>5.42Ser<br>5.46Ser<br>6.48Trp<br>6.51Phe<br>6.52Phe<br>6.55His<br>69Gly<br>7.39Thr<br>7.43Tyr<br>70Gly<br>73Asn<br>74Phe	3.32Asp	1.58Leu<br>2.42Leu<br>2.43Val<br>3.32Asp<br>3.36Cys<br>38Thr<br>6.48Trp<br>6.55His<br>7.43Tyr<br>74Phe	1.58Leu<br>3.32Asp<br>38Thr<br>6.55His<br>7.43Tyr<br>74Phe	2.48Val<br>6.52Phe<br>6.55His	2.45Ser<br>6.51Phe<br>6.52Phe	38Thr<br>7.35Tyr

	75Ser						
Risperidone	1.58Leu		159Ile	159Ile	2.48Val	2.42Leu	1.58Leu
	158Ser		2.43Val	2.43Val	6.55His	2.45Ser	35Ala
	159Ile		2.48Val	3.32Asp		2.46Leu	6.48Trp
	2.41Tyr		2.50Asp	38Thr		34Arg	
	2.42Leu		2.61Val	6.48Trp		6.44Phe	
	2.43Val		3.32Asp	6.55His		6.48Trp	
	2.44Val		3.36Cys	7.43Tyr		6.51Phe	
	2.45Ser		38Thr	74Phe		6.55His	
	2.46Leu		6.48Trp			7.43Tyr	
	2.48Val		6.51Phe				
	2.50Asp		6.55His				
	2.53Val		7.39Thr				
	2.57Val		7.43Tyr				
	2.61Val		74Phe				
	2.64Leu						
	2.65Glu						
	3.28Phe						
	3.29Val						
	3.32Asp						
	3.33Val						
	3.35Met						
	3.36Cys						
	34Arg						
	35Ala						
	38Thr						
	39Thr						
	5.38Phe						
	5.39Val						
	5.42Ser						
	5.46Ser						
	5.47Phe						
	6.44Phe						
	6.47Cys						
	6.48Trp						
	6.51Phe						
	6.52Phe						
	6.55His						
	69Gly						
	7.35Tyr						
	7.38Thr						

	7.39Thr						
	7.42Gly						
	7.43Tyr						
	71Val						
	75Ser						
Aripiprazole	1.39Tyr		159Ile	159Ile	2.48Val	2.46Leu	2.41Tyr
	1.58Leu		2.48Val	2.61Val	2.50Asp	2.50Asp	3.28Phe
	158Ser		2.49Ala	38Thr	38Thr	2.61Val	6.48Trp
	159Ile		2.61Val	5.39Val		34Arg	6.51Phe
	2.42Leu		3.32Asp	5.42Ser		6.51Phe	7.35Tyr
	2.43Val		3.37Thr	5.46Ser		6.52Phe	7.43Tyr
	2.44Val		38Thr	7.43Tyr		7.43Tyr	
	2.45Ser		5.39Val	75Ser		74Phe	
	2.46Leu		5.42Ser				
	2.47Ala		5.43Ser				
	2.48Val		5.46Ser				
	2.49Ala		6.47Cys				
	2.53Val		6.55His				
	2.57Val		7.43Tyr				
	2.61Val		75Ser				
	2.64Leu						
	2.65Glu						
	2.66Val						
	3.28Phe						
	3.29Val						
	3.32Asp						
	3.33Val						
	3.35Met						
	3.36Cys						
	34Arg						
	35Ala						
	5.38Phe						
	5.39Val						
	5.42Ser						
	5.46Ser						
	6.47Cys						
	6.48Trp						
	6.51Phe						
	6.52Phe						
	6.55His						
	7.32Pro						

	7.35Tyr						
	7.36Ser						
	7.38Thr						
	7.39Thr						
	7.42Gly						
	7.43Tyr						
	70Gly						
	71Val						
	74Phe						
	75Ser						
Haloperidole	1.58Leu		1.58Leu	159Ile	2.48Val	2.50Asp	2.48Val
	159Ile		159Ile	3.32Asp		6.48Trp	35Ala
	2.41Tyr		2.41Tyr	5.42Ser		6.51Phe	5.47Phe
	2.42Leu		3.29Val	5.46Ser		6.55His	6.52Phe
	2.43Val		3.32Asp	7.43Tyr			6.55His
	2.44Val		3.33Val	71Val			
	2.45Ser		3.36Cys				
	2.46Leu		35Ala				
	2.47Ala		5.42Ser				
	2.48Val		5.43Ser				
	2.50Asp		5.46Ser				
	2.53Val		6.48Trp				
	2.61Val		6.55His				
	2.65Glu		7.43Tyr				
	2.66Val		71Val				
	3.28Phe						
	3.29Val						
	3.32Asp						
	3.33Val						
	3.36Cys						
	34Arg						
	35Ala						
	38Thr						
	5.38Phe						
	5.39Val						
	5.42Ser						
	5.43Ser						
	5.46Ser						
	5.47Phe						
	6.48Trp						
	6.51Phe						

	6.52Phe<br>6.55His<br>7.35Tyr<br>7.39Thr<br>7.43Tyr<br>71Val<br>73Asn						
Spiperone	1.39Tyr<br>1.44Leu<br>1.58Leu<br>110Met<br>152Gly<br>155Thr<br>156Val<br>157Cys<br>159Ile<br>1Gln<br>2.42Leu<br>2.43Val<br>2.44Val<br>2.45Ser<br>2.46Leu<br>2.47Ala<br>2.48Val<br>2.50Asp<br>2.53Val<br>2.57Val<br>2.61Val<br>2.64Leu<br>2.65Glu<br>2Ala<br>3.23Ile<br>3.24Cys<br>3.27Val<br>3.28Phe<br>3.31Leu<br>3.32Asp<br>3.33Val<br>3.36Cys<br>3.52Thr<br>34Arg	1.44Leu<br>75Ser	1.43Ile<br>2.48Val<br>2.50Asp<br>3.24Cys<br>3.27Val<br>3.33Val<br>3.36Cys<br>3.52Thr<br>38Thr<br>3Arg<br>5.57Val<br>6.48Trp<br>6.55His<br>70Gly<br>72Trp	3.52Thr<br>38Thr<br>5.57Val<br>7.43Tyr<br>72Trp	152Gly	1.35Tyr<br>159Ile<br>2.61Val<br>6.51Phe	2.42Leu<br>2.64Leu<br>35Ala<br>6.48Trp<br>75Ser

	35Ala<br>38Thr<br>3Arg<br>5.38Phe<br>5.42Ser<br>5.53Val<br>5.56Leu<br>5.57Val<br>6.48Trp<br>6.51Phe<br>6.52Phe<br>7.38Thr<br>7.39Thr<br>7.42Gly<br>7.43Tyr<br>70Gly<br>71Val<br>72Trp<br>73Asn<br>74Phe<br>75Ser						
Chlorpromazine	1.54Cys<br>1.58Leu<br>158Ser<br>159Ile<br>2.39Thr<br>2.41Tyr<br>2.42Leu<br>2.43Val<br>2.44Val<br>2.45Ser<br>2.46Leu<br>2.47Ala<br>2.48Val<br>2.53Val<br>2.56Leu<br>2.57Val<br>2.61Val<br>3.28Phe<br>3.29Val<br>3.32Asp		6.48Trp<br>7.43Tyr<br>75Ser		2.48Val	2.14Tyr<br>34Arg<br>6.51Phe	1.58Leu<br>2.42Leu<br>2.43Val<br>2.44Val<br>2.45Ser<br>2.48Val<br>6.48Trp<br>6.51Phe

	3.33Val						
	3.35Met						
	3.36Cys						
	3.40Ile						
	34Arg						
	35Ala						
	38Thr						
	5.38Phe						
	5.39Val						
	5.42Ser						
	5.46Ser						
	5.47Phe						
	6.44Phe						
	6.48Trp						
	6.51Phe						
	6.52Phe						
	6.55His						
	7.38Thr						
	7.39Thr						
	7.42Gly						
	7.43Tyr						
	71Val						
	73Asn						
	74Phe						
	75Ser						

Table S13 - D₄R residues with Ballesteros & Weinstein-numbering [4] **participating in different interaction types sorted by ligands.** Data was summarized for all time points. Abbreviations: Hydrocontacts – hydrophobic contacts; SB – salt-bridges; 2.5 Å – 2.5 Å-interactions; HB – hydrogen bonds; cat-π – cat-π-interactions, T-stack – T-stacking-interactions; π-π-stack – π-π-stacking-interactions. Duplicate residues per interaction type are colored red, while unique residues per interaction type are colored green.

Interaction type	Hydrocontacts	SB	2.5 Å	HB	Cat-π	T-stack	π-π-stack
Dopamine	156Arg	3.32Asp	156Arg	3.32Asp	6.55His	6.52Phe	6.52Phe
	157Leu		3.32Asp	5.42Ser	only at timepoint 65 ns	6.55His	
	3.32Asp		5.39Val	5.43Ser			only at timepoint 65 ns

	3.33Val		5.42Ser	6.55His		only at timepoints 0 and 95 ns	
	3.36Cys		5.43Ser	7.43Tyr			
	3.37Thr		5.46Ser				
	5.39Val		6.55His				
	5.42Ser		7.43Tyr				
	5.43Ser						
	5.46Ser						
	6.48Trp						
	6.51Phe						
	6.52Phe						
	6.55His						
7-OH-DPAT	156Arg	3.32Asp	156Arg	156Arg	6.51Phe	6.52Phe	5.38Tyr
	157Leu		3.36Cys	5.38Tyr	6.48Trp	6.55His	6.51Phe
	2.53Leu		5.39Val	5.39Val	6.55His		6.52Phe
	3.29Met		5.42Ser	5.42Ser			6.55His
	3.32Asp		5.43Ser	5.43Ser			
	3.33Val		5.46Ser	5.46Ser			
	3.36Cys		6.48Trp	6.48Trp			
	5.39Val		6.55His	6.55His			
	5.42Ser						
	5.43Ser						
	5.46Ser						
	5.47Phe						
	6.48Trp						
	6.51Phe						
	6.52Phe						
	6.55His						
	7.39Thr						
	7.42Gly						
	7.43Tyr						
Apomorphine	156Arg		156Arg	156Arg	156Arg	6.48Trp	6.52Phe
	157Leu		3.36Cys	5.42Ser	6.55His	6.51Phe	6.55His
	3.29Met		3.37Thr	5.43Ser		6.52Phe	
	3.32Asp		5.39Val	5.46Ser		6.55His	
	3.33Val		5.42Ser	6.55His			
	3.36Cys		5.43Ser				
	3.37Thr		5.46Ser				
	3.40Ile		6.55His				
	4.56Val						

	5.39Val<br>5.42Ser<br>5.43Ser<br>5.46Ser<br>6.44Phe<br>6.48Trp<br>6.51Phe<br>6.52Phe<br>6.55His<br>7.35Val<br>7.38Val<br>7.39Thr<br>7.43Tyr						
Bromocriptine	156Arg<br>157Leu<br>2.53Leu<br>3.29Met<br>3.32Asp<br>3.33Val<br>3.36Cys<br>3.37Thr<br>3.39Ser<br>3.40Ile<br>3.41Phe<br>4.52Leu<br>4.53Ser<br>4.56Val<br>5.36Arg<br>5.38Tyr<br>5.39Val<br>5.40Val<br>5.41Tyr<br>5.42Ser<br>5.43Ser<br>5.45Cys<br>5.46Ser<br>5.47Phe<br>5.48Phe<br>6.44Phe<br>6.48Trp<br>6.51Phe		156Arg<br>157Leu<br>2.53Leu<br>3.29Met<br>3.32Asp<br>3.33Val<br>3.36Cys<br>3.37Thr<br>3.40Ile<br>3.41Phe<br>4.52Leu<br>4.56Val<br>5.36Arg<br>5.38Tyr<br>5.39Val<br>5.42Ser<br>5.43Ser<br>5.45Cys<br>5.46Ser<br>5.47Phe<br>6.44Phe<br>6.48Trp<br>6.51Phe<br>6.52Phe<br>6.55His<br>6.56Ile<br>7.35Val<br>7.38Val	156Arg<br>157Leu<br>3.32Asp<br>3.36Cys<br>4.56Val<br>5.36Arg<br>5.43Ser<br>6.48Trp<br>6.55His<br>7.38Val<br>7.39Thr<br>7.43Tyr	156Arg<br>6.55His	6.48Trp	6.48Trp<br>6.51Phe<br>6.52Phe<br>6.55His

	6.52Phe<br>6.54Val<br>6.55His<br>6.56Ile<br>7.35Val<br>7.36Ser<br>7.38Val<br>7.39Thr<br>7.42Gly<br>7.43Tyr		7.39Thr<br>7.42Gly<br>7.43Tyr				
Clozapine	155Cys<br>156Arg<br>157Leu<br>2.53Leu<br>3.29Met<br>3.32Asp<br>3.33Val<br>3.36Cys<br>3.37Thr<br>3.40Ile<br>4.56Val<br>5.38Tyr<br>5.39Val<br>5.42Ser<br>5.43Ser<br>5.46Ser<br>6.44Phe<br>6.47Cys<br>6.48Trp<br>6.51Phe<br>6.52Phe<br>6.55His<br>7.35Val<br>7.38Val<br>7.39Thr<br>7.42Gly<br>7.43Tyr		156Arg<br>157Leu<br>3.32Asp<br>3.33Val<br>3.36Cys<br>5.39Val<br>5.42Ser<br>5.46Ser<br>6.48Trp<br>6.51Phe<br>6.52Phe<br>6.55His<br>7.43Tyr	156Arg<br>3.33Val<br>5.42Ser<br>5.46Ser<br>6.55His<br>7.43Tyr	156Arg<br>6.55His	6.48Trp<br>6.51Phe<br>6.52Phe<br>7.43Tyr	6.48Trp<br>6.51Phe<br>6.55His
Nemonapride	154Val<br>155Cys<br>156Arg<br>157Leu	3.32Asp	156Arg<br>3.32Asp<br>3.36Cys<br>5.42Ser	156Arg<br>3.32Asp<br>5.42Ser<br>5.46Ser	6.51Phe<br>6.55His<br>7.43Tyr	6.48Trp<br>6.51Phe<br>6.52Phe<br>6.55His	6.48Trp<br>6.51Phe<br>7.43Tyr

	2.53Leu		5.46Ser	6.48Trp		7.43Tyr	
	2.57Val		6.48Trp	7.42Gly			
	2.58Leu		6.55His	7.43Tyr			
	2.61Phe		7.42Gly				
	3.28Leu		7.43Tyr				
	3.29Met		7.46Ser				
	3.32Asp						
	3.33Val						
	3.35Leu						
	3.36Cys						
	3.37Thr						
	3.39Ser						
	4.56Val						
	5.38Tyr						
	5.39Val						
	5.42Ser						
	5.43Ser						
	5.46Ser						
	5.47Phe						
	6.44Phe						
	6.48Trp						
	6.51Phe						
	6.52Phe						
	6.55His						
	7.39Thr						
	7.42Gly						
	7.43Tyr						
	7.45Asn						
	7.46Ser						
Sulpiride	154Val	3.32Asp	156Arg	156Arg	6.52Phe	6.48Trp	154Val
	155Cys		3.29Met	3.32Asp	6.55His	6.51Phe	155Cys
	156Arg		3.32Asp	5.42Ser		6.52Phe	156Arg
	157Leu		3.33Val	5.43Ser			157Leu
	2.53Leu		3.36Cys	5.46Ser			2.53Leu
	2.57Val		4.56Val	6.48Trp			2.57Val
	3.29Met		5.39Val	6.55His			3.29Met
	3.32Asp		5.42Ser	7.43Tyr			3.32Asp
	3.33Val		5.43Ser				3.33Val
	3.35Leu		5.46Ser				3.35Leu
	3.36Cys		6.48Trp				3.36Cys
	3.39Ser		6.51Phe				3.39Ser

	4.56Val<br>5.38Tyr<br>5.39Val<br>5.42Ser<br>5.46Ser<br>6.48Trp<br>6.51Phe<br>6.52Phe<br>6.55His<br>6.58Gln<br>7.35Val<br>7.38Val<br>7.39Thr<br>7.42Gly<br>7.43Tyr		6.52Phe<br>6.55His<br>7.43Tyr				4.56Val<br>5.38Tyr<br>5.39Val<br>5.42Ser<br>5.46Ser<br>6.48Trp<br>6.51Phe<br>6.52Phe<br>6.55His<br>6.58Gln<br>7.35Val<br>7.38Val<br>7.39Thr<br>7.42Gly<br>7.43Tyr
SCH23390	155Cys<br>156Arg<br>157Leu<br>3.29Met<br>3.32Asp<br>3.33Val<br>3.36Cys<br>5.38Tyr<br>5.39Val<br>5.42Ser<br>5.43Ser<br>5.46Ser<br>5.47Phe<br>6.48Trp<br>6.51Phe<br>6.52Phe<br>6.55His<br>7.39Thr<br>7.43Tyr	3.32Asp	156Arg<br>157Leu<br>3.32Asp<br>3.37Thr<br>5.39Val<br>5.42Ser<br>5.43Ser<br>5.46Ser<br>6.48Trp<br>6.55His<br>7.43Tyr	156Arg<br>3.32Asp<br>5.42Ser<br>6.55His	156Arg<br>6.51Phe<br>6.52Phe<br>6.55His	6.51Phe<br>6.52Phe<br>6.55His	6.51Phe<br>6.52Phe<br>6.55His
SKF38393	155Cys<br>156Arg<br>157Leu<br>2.53Leu<br>3.29Met<br>3.32Asp<br>3.33Val	3.32Asp	156Arg<br>3.32Asp<br>3.36Cys<br>5.39Val<br>5.42Ser<br>5.43Ser<br>5.46Ser	3.32Asp<br>5.39Val<br>5.42Ser<br>5.43Ser<br>5.46Ser<br>7.42Gly<br>7.43Tyr	156Arg<br>6.52Phe<br>6.55His	5.47Phe<br>6.51Phe<br>6.52Phe<br>6.55His<br>7.43Tyr	6.44Phe<br>6.48Trp<br>6.51Phe<br>6.52Phe

	3.36Cys<br>5.38Tyr<br>5.39Val<br>5.42Ser<br>5.43Ser<br>5.46Ser<br>6.48Trp<br>6.51Phe<br>6.52Phe<br>6.55His<br>7.38Val<br>7.39Thr<br>7.42Gly<br>7.43Tyr		6.55His<br>7.38Val<br>7.42Gly<br>7.43Tyr				
Eticlopride	154Val<br>155Cys<br>156Arg<br>157Leu<br>2.53Leu<br>2.61Phe<br>3.28Leu<br>3.29Met<br>3.32Asp<br>3.33Val<br>3.35Leu<br>3.36Cys<br>3.37Thr<br>3.39Ser<br>5.38Tyr<br>5.39Val<br>5.42Ser<br>5.43Ser<br>5.46Ser<br>6.48Trp<br>6.51Phe<br>6.52Phe<br>6.55His<br>7.39Thr<br>7.42Gly<br>7.43Tyr<br>7.46Ser	2.50Asp<br>3.32Asp	156Arg<br>157Leu<br>3.32Asp<br>3.33Val<br>3.36Cys<br>5.42Ser<br>6.55His<br>7.43Tyr	156Arg<br>3.32Asp<br>3.33Val<br>6.55His<br>7.43Tyr	156Arg<br>6.51Phe<br>6.55His	6.48Trp<br>6.51Phe<br>6.52Phe<br>6.55His	

Risperidone	154Val		156Arg	156Arg	156Arg	6.51Phe	5.38Tyr
	155Cys		157Leu	157Leu	6.55His	6.52Phe	6.48Trp
	156Arg		2.64Ser	3.32Asp		6.55His	6.55His
	157Leu		3.32Asp	6.48Trp		7.43Tyr	
	2.53Leu		3.36Cys	6.55His			
	2.61Phe		3.37Thr	7.43Tyr			
	2.64Ser		5.39Val				
	2.65Glu		5.42Ser				
	3.28Leu		5.43Ser				
	3.29Met		6.48Trp				
	3.32Asp		6.51Phe				
	3.33Val		6.52Phe				
	3.35Leu		6.55His				
	3.36Cys		7.43Tyr				
	3.37Thr						
	3.39Ser						
	4.56Val						
	5.38Tyr						
	5.39Val						
	5.42Ser						
	5.43Ser						
	5.46Ser						
	6.44Phe						
	6.48Trp						
	6.51Phe						
	6.52Phe						
	6.54Val						
	6.55His						
	69Gly						
	7.35Val						
	7.36Ser						
	7.39Thr						
	7.42Gly						
	7.43Tyr						
	7.45Asn						
	71Trp						
Aripiprazole	154Val		156Arg	156Arg	156Arg	6.48Trp	6.48Trp
	156Arg		1Ala	5.36Arg		6.51Phe	7.43Tyr
	157Leu		3.31Met	5.42Ser		6.52Phe	
	2.53Leu		3.32Asp	5.43Ser		7.43Tyr	
	2.57Val		3.36Cys	5.46Ser			

	2.61Phe		3.37Thr	6.48Trp			
	2.64Ser		5.36Arg				
	3.28Leu		5.39Val				
	3.29Met		5.42Ser				
	3.32Asp		5.43Ser				
	3.33Val		5.46Ser				
	3.36Cys		6.48Trp				
	3.37Thr		6.55His				
	4.56Val		7.43Tyr				
	5.39Val		70Ala				
	5.42Ser						
	5.43Ser						
	5.46Ser						
	6.48Trp						
	6.51Phe						
	6.52Phe						
	6.55His						
	7.38Val						
	7.39Thr						
	7.42Gly						
	7.43Tyr						
	7.46Ser						
	154Val		156Arg	156Arg			
	156Arg		157Leu				
	157Leu		2.64Ser				
	2.53Leu		3.32Asp				
	2.57Val		3.36Cys				
	2.60Leu		5.36Arg				
	2.61Phe		5.42Ser				
	2.64Ser		6.48Trp				
	3.28Leu		6.55His				
Haloperidole	3.29Met		7.42Gly				
	3.32Asp		7.43Tyr				
	3.33Val		7.46Ser				
	3.36Cys						
	3.37Thr						
	3.40Ile						
	4.56Val						
	5.38Tyr						
	5.39Val						
	5.41Tyr						

	5.42Ser						
	5.43Ser						
	5.45Cys						
	5.46Ser						
	5.47Phe						
	6.44Phe						
	6.48Trp						
	6.51Phe						
	6.52Phe						
	6.55His						
	7.36Ser						
	7.39Thr						
	7.42Gly						
	7.43Tyr						
	7.45Asn						
	7.46Ser						
Spiperone	154Val	3.32Asp	156Arg	156Arg	156Arg	6.48Trp	6.48Trp
	155Cys		2.53Leu	5.39Val		6.51Phe	6.51Phe
	156Arg		3.36Cys	6.48Trp	only at timepoint 60 and 70 ns	6.52Phe	6.55His
	157Leu		5.38Tyr	6.55His		6.55His	
	2.53Leu		5.39Val	7.43Tyr		7.43Tyr	
	2.61Phe		5.42Ser				
	3.28Leu		5.43Ser				
	3.29Met		5.46Ser				
	3.32Asp		6.48Trp				
	3.33Val		6.55His				
	3.35Leu		7.43Tyr				
	3.36Cys						
	3.37Thr						
	3.39Ser						
	3.40Ile						
	4.56Val						
	5.38Tyr						
	5.39Val						
	5.42Ser						
	5.43Ser						
	5.46Ser						
	6.44Phe						

	6.48Trp<br>6.51Phe<br>6.52Phe<br>6.54Val<br>6.55His<br>7.35Val<br>7.36Ser<br>7.38Val<br>7.39Thr<br>7.42Gly<br>7.43Tyr<br>7.45Asn						
Chlorpromazine	156Arg<br>157Leu<br>2.53Leu<br>3.29Met<br>3.32Asp<br>3.33Val<br>3.36Cys<br>3.37Thr<br>3.39Ser<br>3.40Ile<br>5.39Val<br>5.42Ser<br>5.43Ser<br>5.46Ser<br>5.47Phe<br>6.44Phe<br>6.47Cys<br>6.48Trp<br>6.51Phe<br>6.52Phe<br>6.55His<br>7.35Val<br>7.38Val<br>7.39Thr<br>7.42Gly<br>7.43Tyr		156Arg	6.48Trp	156Arg<br>6.55His	6.48Trp<br>6.51Phe<br>6.52Phe<br>6.55His<br>7.43Tyr	6.48Trp<br>6.51Phe<br>6.52Phe

Table S14 - D5R residues with Ballesteros & Weinstein-numbering [4] **participating in different interaction types sorted by ligands.** Data was summarized for all time points. Abbreviations: Hydrocontacts – hydrophobic contacts; SB – salt-bridges; 2.5 Å – 2.5 Å-interactions; HB – hydrogen bonds; cat-π – cat-π-interactions, T-stack – T-stacking-interactions; π-π-stack – π-π-stacking-interactions. Duplicate residues per interaction type are colored red, while unique residues per interaction type are colored green.

Interaction type	Hydrocontacts	SB	2.5 Å	HB	Cat-π	T-stack	π-π-stack
Dopamine	3.32Asp<br>3.33Ile<br>3.36Ser<br>5.42Ser<br>5.46Ser<br>6.48Trp<br>6.51Phe<br>6.52Phe<br>6.55Asn	3.32Asp	3.32Asp<br>5.39Ala<br>5.42Ser<br>5.43Ser<br>5.46Ser<br>6.55Asn	3.32Asp<br>5.38Tyr<br>5.42Ser<br>5.43Ser<br>6.55Asn		6.48Trp<br>6.51Phe<br>6.52Phe	6.52Phe<br>5.38Tyr
7-OH-DPAT	3.28Trp<br>3.29Val<br>3.32Asp<br>3.33Ile<br>3.36Ser<br>5.35Asn<br>5.39Ala<br>5.42Ser<br>5.43Ser<br>5.46Ser<br>6.48Trp<br>6.51Phe<br>6.52Phe<br>6.55Asn<br>7.35Phe<br>7.39Val<br>7.42Gly	3.32Asp	5.39Ala<br>5.42Ser<br>5.43Ser<br>5.46Ser<br>6.48Trp<br>6.55Asn	5.39Ala<br>5.42Ser<br>5.43Ser<br>5.46Ser<br>6.55Asn<br>only with No 11		6.52Phe	6.52Phe
Apomorphine	164Leu<br>3.28Trp<br>3.29Val<br>3.32Asp<br>3.33Ile		5.39Ala<br>5.42Ser<br>5.43Ser<br>5.46Ser<br>6.55Asn	5.39Ala<br>5.42Ser<br>5.43Ser<br>5.46Ser<br>6.55Asn		6.48Trp<br>6.51Phe<br>6.52Phe<br>7.35Phe	5.38Tyr<br>7.35Phe<br>only at timepoints 85, 90 ns

	3.36Ser						
	5.35Asn						
	5.39Ala						
	5.42Ser						
	5.43Ser						
	5.46Ser						
	6.48Trp						
	6.51Phe						
	6.52Phe						
	6.55Asn						
	7.35Phe						
Bromocriptine	164Leu		164Leu	3.32Asp		6.48Trp	6.48Trp
	2.53Val		2.57Val	3.39Ser		7.35Phe	6.52Phe
	2.57Val		3.28Trp	5.42Ser			
	3.28Trp		3.32Asp	5.43Ser			
	3.29Val		3.33Ile	6.55Asn			
	3.32Asp		3.36Ser	7.45Asn			
	3.33Ile		3.39Ser				
	3.35Cys		5.35Asn				
	3.36Ser		5.39Ala				
	3.40Ile		5.42Ser				
	5.35Asn		5.46Ser				
	5.38Tyr		6.48Trp				
	5.39Ala		6.52Phe				
	5.42Ser		6.55Asn				
	5.43Ser		7.35Phe				
	5.46Ser		7.39Val				
	5.47Phe		7.45Asn				
	6.44Phe		7.49Asn				
	6.47Cys						
	6.48Trp						
	6.51Phe						
	6.52Phe						
	6.55Asn						
	7.35Phe						
	7.38Phe						
	7.39Val						
	7.42Gly						
	7.45Asn						
Clozapine	164Leu		3.32Asp	3.32Asp		6.51Phe	5.38Tyr
	2.53Val		5.35Asn	5.42Ser		6.52Phe	6.48Trp

	2.57Val<br>3.28Trp<br>3.29Val<br>3.32Asp<br>3.33Ile<br>3.35Cys<br>3.36Ser<br>3.39Ser<br>5.38Tyr<br>5.39Ala<br>5.42Ser<br>5.43Ser<br>5.46Ser<br>6.44Phe<br>6.47Cys<br>6.48Trp<br>6.51Phe<br>6.52Phe<br>6.55Asn<br>7.35Phe<br>7.39Val<br>7.42Gly<br>7.45Asn		5.39Ala<br>5.42Ser<br>6.55Asn<br>7.45Asn	6.55Asn<br>7.45Asn		7.35Phe	6.52Phe
Nemonapride	2.53Val<br>2.57Val<br>2.58Met<br>2.61Lys<br>3.28Trp<br>3.29Val<br>3.32Asp<br>3.33Ile<br>3.36Ser<br>5.39Ala<br>5.42Ser<br>5.43Ser<br>5.46Ser<br>6.48Trp<br>6.51Phe<br>6.52Phe<br>6.55Asn<br>7.38Phe	3.32Asp	3.32Asp<br>3.36Ser<br>5.42Ser<br>5.46Ser<br>6.48Trp<br>6.55Asn	3.32Asp<br>5.42Ser<br>5.46Ser<br>6.48Trp<br>6.55Asn	3.28Trp	6.48Trp<br>6.51Phe<br>6.52Phe	6.48Trp<br>6.51Phe<br>6.52Phe

	7.39Val						
	7.42Gly						
	7.43Trp						
	7.46Ser						
Sulpiride	2.53Val	3.32Asp	2.61Lys	3.29Val	6.51Phe	6.48Trp	3.28Trp
	2.57Val		3.29Val	3.32Asp		6.51Phe	6.48Trp
	2.58Met		3.32Asp	3.36Ser		6.52Phe	
	2.61Lys		3.36Ser	5.39Ala			
	3.28Trp		5.39Ala	5.42Ser			
	3.29Val		5.42Ser	5.43Ser			
	3.32Asp		5.43Ser	6.48Trp			
	3.33Ile		5.46Ser	6.55Asn			
	3.36Ser		6.48Trp				
	5.39Ala		6.52Phe				
	5.42Ser		6.55Asn				
	6.48Trp						
	6.51Phe						
	6.52Phe						
	6.55Asn						
	7.35Phe						
	7.38Phe						
	7.39Val						
	7.42Gly						
	7.43Trp						
SCH23390	2.53Val	3.32Asp	5.39Ala	5.39Ala		6.48Trp	6.48Trp
	3.28Trp		5.42Ser			6.51Phe	6.51Phe
	3.29Val		5.43Ser			6.52Phe	
	3.32Asp		5.46Ser				
	3.33Ile		6.48Trp				
	3.36Ser		6.51Phe				
	5.39Ala		6.55Asn				
	5.42Ser						
	5.46Ser						
	6.48Trp						
	6.51Phe						
	6.52Phe						
	6.55Asn						
	7.35Phe						
	7.39Val						
	7.42Gly						
SKF38393	3.28Trp	3.32Asp	3.32Asp	3.32Asp		6.51Phe	5.38Tyr

	3.29Val<br>3.32Asp<br>3.33Ile<br>3.36Ser<br>5.39Ala<br>5.42Ser<br>5.43Ser<br>6.48Trp<br>6.51Phe<br>6.52Phe<br>6.55Asn<br>7.35Phe<br>7.38Phe<br>7.39Val		5.39Ala<br>5.42Ser<br>5.43Ser<br>5.46Ser<br>6.48Trp<br>6.52Phe<br>6.55Asn	5.39Ala<br>5.42Ser<br>5.43Ser<br>5.46Ser<br>6.48Trp<br>6.55Asn		6.52Phe<br>7.35Phe	6.48Trp<br>6.51Phe<br>6.52Phe
Eticlopride	2.53Val<br>2.57Val<br>2.58Met<br>2.61Lys<br>3.28Trp<br>3.29Val<br>3.32Asp<br>3.33Ile<br>3.35Cys<br>3.36Ser<br>5.39Ala<br>5.42Ser<br>5.43Ser<br>5.46Ser<br>5.47Phe<br>6.48Trp<br>6.51Phe<br>6.52Phe<br>6.55Asn<br>7.35Phe<br>7.39Val<br>7.42Gly<br>7.43Trp		3.32Asp<br>3.36Ser<br>5.39Ala<br>5.43Ser<br>6.48Trp<br>6.55Asn	6.48Trp<br>6.51Phe<br>6.52Phe	6.48Trp<br>6.51Phe<br>6.52Phe	6.48Trp<br>6.51Phe	3.32Asp<br>6.51Phe
Risperidone	164Leu<br>2.53Val<br>2.57Val<br>2.58Met		2.57Val<br>3.28Trp<br>3.32Asp<br>3.35Cys	2.57Val<br>3.32Asp<br>6.48Trp<br>6.55Asn		6.48Trp<br>6.51Phe<br>6.52Phe	5.38Tyr<br>6.48Trp<br>6.51Phe<br>7.35Phe

	2.61Lys		5.35Asn				
	3.28Trp		5.42Ser				
	3.29Val		5.46Ser				
	3.32Asp		6.48Trp				
	3.33Ile		6.55Asn				
	3.36Ser						
	5.35Asn						
	5.38Tyr						
	5.39Ala						
	5.42Ser						
	5.43Ser						
	5.46Ser						
	6.48Trp						
	6.51Phe						
	6.52Phe						
	6.55Asn						
	7.35Phe						
	7.39Val						
	7.42Gly						
	7.43Trp						
	7.46Ser						
Aripiprazole	164Leu		164Leu	164Leu	2.61Lys	6.48Trp	5.38Tyr
	2.53Val		2.61Lys	2.61Lys		6.51Phe	6.48Trp
	2.57Val		3.28Trp	3.32Asp		6.52Phe	6.51Phe
	2.58Met		3.32Asp	5.35Asn			
	2.61Lys		5.39Ala	5.42Ser			
	3.28Trp		5.42Ser	5.46Ser			
	3.29Val		5.43Ser	6.48Trp			
	3.32Asp		5.46Ser	6.55Asn			
	3.33Ile		6.55Asn				
	3.35Cys						
	3.36Ser						
	5.35Asn						
	5.38Tyr						
	5.39Ala						
	5.42Ser						
	5.43Ser						
	5.46Ser						
	6.48Trp						
	6.51Phe						
	6.52Phe						

	6.55Asn<br>7.35Phe<br>7.39Val<br>7.42Gly<br>7.43Trp<br>7.46Ser						
Haloperidole	2.53Val<br>2.57Val<br>2.58Met<br>2.61Lys<br>3.28Trp<br>3.29Val<br>3.32Asp<br>3.33Ile<br>3.35Cys<br>3.36Ser<br>3.39Ser<br>5.39Ala<br>5.42Ser<br>5.43Ser<br>5.46Ser<br>5.47Phe<br>6.44Phe<br>6.47Cys<br>6.48Trp<br>6.51Phe<br>6.52Phe<br>6.55Asn<br>7.38Phe<br>7.39Val<br>7.41Phe<br>7.42Gly<br>7.43Trp<br>7.46Ser	3.32Asp	3.32Asp<br>3.35Cys<br>3.36Ser<br>5.46Ser<br>6.48Trp<br>6.55Asn<br>7.49Asn	3.32Asp	6.48Trp<br>6.51Phe	6.51Phe<br>6.52Phe	6.48Trp<br>6.51Phe<br>6.52Phe
Spiperone	2.53Val<br>2.57Val<br>2.58Met<br>2.61Lys<br>3.28Trp<br>3.29Val<br>3.32Asp	3.32Asp	3.28Trp<br>5.46Ser<br>6.48Trp<br>6.55Asn	3.28Trp<br>5.46Ser<br>6.48Trp	6.48Trp		6.48Trp

	3.33Ile<br>3.35Cys<br>3.36Ser<br>5.38Tyr<br>5.39Ala<br>5.42Ser<br>5.43Ser<br>5.46Ser<br>6.47Cys<br>6.48Trp<br>6.51Phe<br>6.52Phe<br>6.55Asn<br>7.35Phe<br>7.39Val<br>7.42Gly<br>7.43Trp<br>7.46Ser						
Chlorpromazine	3.28Trp<br>3.29Val<br>3.32Asp<br>3.33Ile<br>3.35Cys<br>3.36Ser<br>5.38Tyr<br>5.39Ala<br>5.42Ser<br>5.43Ser<br>5.46Ser<br>6.48Trp<br>6.51Phe<br>6.52Phe<br>6.55Asn<br>7.35Phe<br>7.39Val<br>7.42Gly					6.51Phe<br>6.52Phe	5.38Tyr

REFERENCES

[1] Wang, S.; Che, T.; Levit, A.; Shoichet, B. K.; Wacker, D.; Roth, B. L. Structure of the D2 dopamine receptor bound to the atypical antipsychotic drug risperidone. *Nat. 2018* **2018**, 1–24.

[2] Chien, E. Y. T.; Liu, W.; Zhao, Q.; Katritch, V.; Han, G. W.; Michael, a; Shi, L.; Newman, A. H.; Javitch, J. a; Cherezov, V.; Stevens, R. C. Structure of the human dopamine D3 receptor in complex with a D2/D3 selective antagonist. *Science (80-.).* **2011**, *330* (6007), 1091–1095.

[3] Wang, S.; Wacker, D.; Levit, A.; Che, T.; Betz, R. M.; Mccorvy, J. D.; Venkatakrishnan, A. J.; Huang, X.-P.; Dror, R. O.; Shoichet, B. K.; Roth, B. L. D 4 dopamine receptor high-resolution structures enable the discovery of selective agonists. **2017**.

[4] Ballesteros, J. A.; Weinstein, H. Integrated methods for the construction of three dimensional models and computational probing of structure-function relations in G-protein coupled receptors. *Methods Neurosci.* **1995**, *25*, 366–428.
